# Comparative evaluation of green-synthesized CeO_2_-rich and multi-rare-earth-containing oxide-rich nanoparticles prepared using *Moringa oleifera* seed extract: physicochemical characterization and preliminary cell-free screening

**DOI:** 10.1039/d6na00394j

**Published:** 2026-09-25

**Authors:** Nader Anis, Nada Emam, Mona M. Khalifa, A. I. L. Abd El Fatah

**Affiliations:** a Reactors Materials Department, Nuclear Materials Authority Cairo Egypt naderanis@science.zu.edu.eg anis.nader@gmail.com; b Department of Biochemistry, Faculty of Science, Cairo University Egypt

## Abstract

Fresh aqueous extracts were independently prepared from the same collected *Moringa oleifera* seed lot and used as biogenic media for the paired preparation of CeO_2_-rich nanoparticles (CeO_2_ NPs) and Ce-rich, multi-rare-earth-containing oxide-rich nanoparticles (multi-REO NPs). Three independent synthesis batches were prepared for each nanoparticle system under standardized extraction and harmonized reaction and post-synthesis conditions. The physicochemical properties of both nanoparticle systems were investigated using UV-vis spectroscopy, FTIR, XRD, SEM, EDS analysis/mapping, TEM/SAED, DLS, and zeta-potential measurements, while HPLC profiling was performed to characterize the phytochemical constituents of the *M. oleifera* seed extract. TEM analysis showed that CeO_2_ NPs were predominantly quasi-spherical, with projected particle dimensions of approximately 5–15 nm, whereas multi-REO NPs exhibited larger projected particle dimensions of approximately 20–60 nm. Multi-REO NPs also exhibited more numerous and comparatively better-resolved diffraction features based on XRD and TEM-SAED analyses. Zeta-potential measurements revealed stronger electrostatic stabilization for CeO_2_ NPs (−25.5 mV) than for multi-REO NPs (−5.09 mV). Multi-REO NPs generally produced greater apparent cell-free assay responses than CeO_2_ NPs at equivalent concentrations. Multi-REO NPs exhibited estimated IC_50_ values of 115.3 and 88.2 µg mL^−1^ for DPPH˙ and ABTS˙^+^ scavenging, respectively, compared with 71.4 and 55.5 µg mL^−1^ for ascorbic acid. The estimated IC_50_ values were 129.1 µg mL^−1^ for α-amylase inhibition, compared with 79.57 µg mL^−1^ for acarbose, and 159.6 µg mL^−1^ for BSA thermal-denaturation inhibition, compared with 121.5 µg mL^−1^ for diclofenac sodium. CeO_2_ NPs did not reach 50% response within the tested range for these endpoints, whereas neither nanoparticle system exhibited a monotonic concentration-dependent AChE-inhibitory response. Overall, the two compositionally distinct nanoparticle systems exhibited different physicochemical, colloidal, and assay-specific profiles, with multi-REO NPs generally showing greater cell-free responses. However, the findings do not establish rare-earth synergy, biological safety, or therapeutic efficacy, and require further mechanistic, interference-control, cytotoxicity, cellular, and *in vivo* evaluation.

## Abstract 2

*Moringa oleifera* seed extract produced CeO_2_-rich and multi-rare-earth oxide-rich nanoparticles with distinct colloidal and cell-free screening profiles.

## Graphical abstract

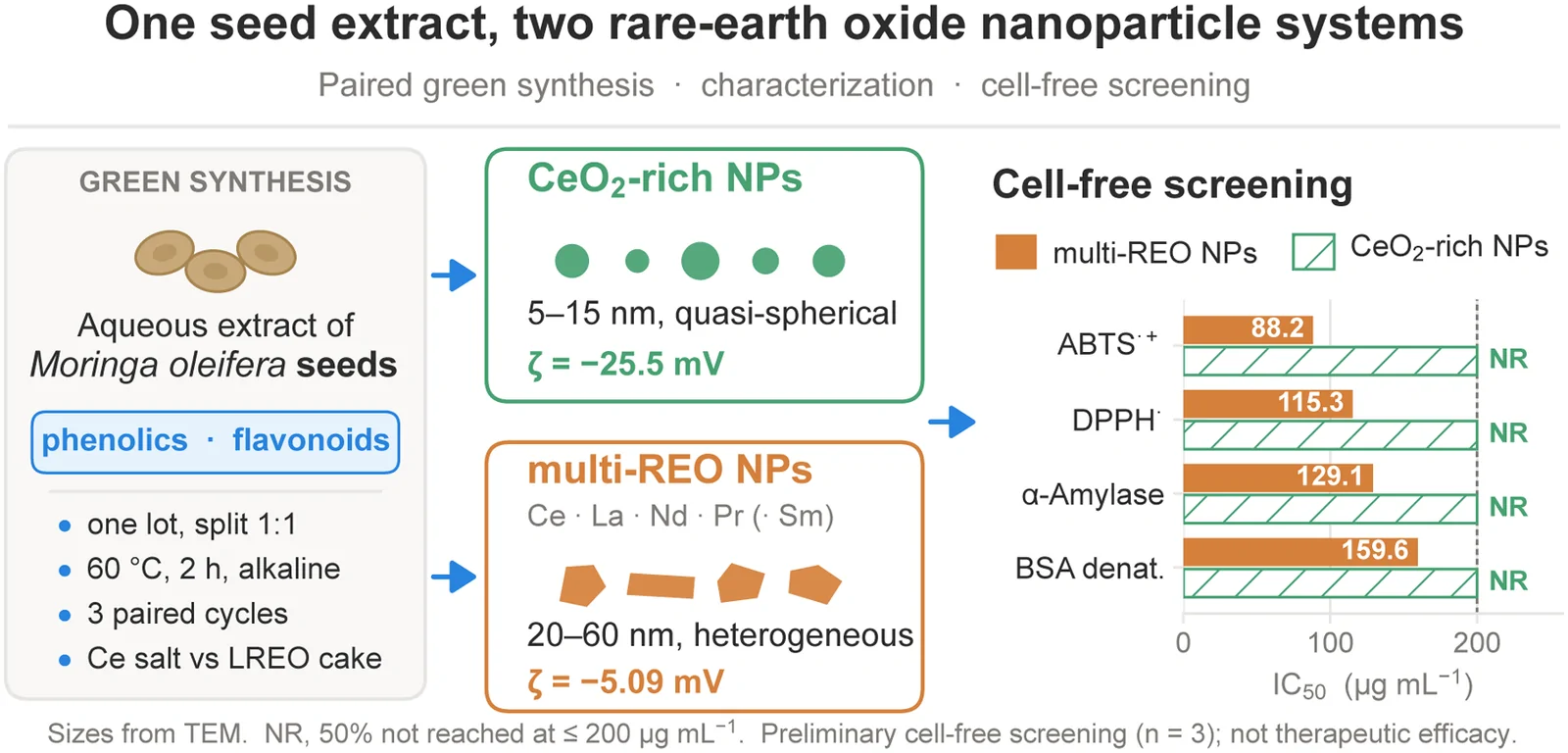

## 1. Introduction

At the nanoscale, metal oxides can exhibit properties that differ substantially from those of their bulk counterparts because of changes in the surface-area-to-volume ratio, crystallographic ordering, defect-related features, surface chemistry, and interfacial reactivity. Consequently, particle dimensions, morphology, aggregation state, phase composition, and surface functionalization may strongly influence their optical, catalytic, colloidal, and interfacial responses.^1^ Rare-earth oxide nanomaterials are of particular interest because the characteristic electronic structures and coordination chemistry of rare-earth elements can support distinctive optical, magnetic, sensing, catalytic, and redox-related properties.^2^ However, conventional preparation methods may require harsh reaction conditions, energy-intensive processing, and potentially hazardous reagents, motivating the development of more environmentally compatible synthesis routes.^3–5^

Rare-earth elements conventionally comprise the fifteen lanthanides together with scandium and yttrium. Although the trivalent oxidation state predominates for many rare-earth elements, some elements, particularly Ce and Pr, can adopt additional oxidation states in specific oxide environments.^6,7^ At the nanoscale, rare-earth oxides provide high surface-area-to-volume ratios and chemically active interfaces at which surface hydroxylation, Lewis acid–base behavior, local coordination environments, defect-related features, and variable oxidation states may influence material properties.^8,9^

Plant-mediated synthesis provides a comparatively accessible route for preparing metal-oxide nanoparticles under mild aqueous conditions. Plant extracts contain naturally derived constituents that may participate in metal-ion coordination, regulation of hydrolysis and nucleation, particle-growth control, and surface functionalization. In rare-earth oxide synthesis, these roles are more appropriately described in terms of coordination- and hydrolysis-mediated processes than conventional reduction of metal ions to zero-valent states.^10,11^ Plant-mediated routes also avoid the controlled culture conditions required for microbial synthesis, although variability in extract composition and preparation may affect nanoparticle reproducibility.^12,13^

Plant-based routes may therefore reduce operational complexity while allowing reaction variables to be optimized to improve nanoparticle formation, dispersion, and size consistency.^3^

Extract-derived constituents may remain associated with nanoparticle surfaces and influence surface functionality, aggregation, electrokinetic behavior, and interactions with reactive species or proteins. Previous studies of phytogenic metal oxides have similarly shown that the biological source, surface-associated constituents, and post-synthesis processing can influence nanoparticle structure, surface chemistry, and preliminary cell-free assay responses.^14–16^ However, such responses should not be attributed exclusively to the inorganic nanoparticle cores because residual or surface-associated phytochemicals may also contribute.

Comparative studies of monometallic and compositionally complex multi-rare-earth-containing oxide systems remain limited. Most phytogenic studies examine individual rare-earth oxides, whereas the coexistence of several rare-earth constituents may be associated with differences in local coordination environments, phase complexity, morphology, surface chemistry, optical behavior, and colloidal properties. These differences may also influence interactions with reactive species and biomolecules, although such effects cannot be attributed to elemental composition alone. A controlled comparative framework using the same biological medium and harmonized processing conditions can reduce synthesis-related variability and enable composition-associated differences to be examined more directly.^11^

Among rare-earth oxides, CeO_2_ has been studied extensively because Ce-containing surface environments can exhibit redox behavior associated with Ce^3+^/Ce^4+^ interconversion and oxygen storage or release. These characteristics, together with surface hydroxylation and defect-related features, have motivated the investigation of nanoceria in catalytic, environmental, and biomedical-interface applications.^17,18^ The coexistence of additional rare-earth constituents in compositionally complex oxide-rich materials may alter local coordination environments, phase complexity, morphology, surface chemistry, optical-edge behavior, and colloidal properties.^19,20^ However, oxidation-state distributions, oxygen-vacancy concentrations, improved colloidal stability, biocompatibility, and reduced toxicity cannot be inferred solely from elemental composition and require direct experimental validation.^11,21,22^

*Moringa oleifera* seeds provide a chemically complex biogenic medium containing proteins, amino acids, phenolic compounds, flavonoids, alkaloids, terpenoid-related compounds, and saponin- or steroid-related constituents.^23–25^ In the present study, targeted HPLC-DAD profiling identified selected phenolic and flavonoid constituents in the aqueous seed extract, complementing the qualitative phytochemical and FTIR findings. However, the identities and abundances of specific extract-derived molecules associated with the final nanoparticle surfaces were not determined directly.

Despite the growing literature on biogenic rare-earth oxide nanomaterials, a methodological gap remains in controlled comparative studies. Published comparisons often involve differences in biological source, precursor chemistry, reaction conditions, purification procedures, and characterization methods, making it difficult to distinguish composition-associated effects from synthesis- and processing-related variability.^26,27^ This limitation is particularly relevant to rare-earth-containing oxide systems because changes in precursor chemistry, elemental composition, phase identity, surface functionality, and aggregation state may concurrently influence their physicochemical, colloidal, and interfacial responses.^28^

To the best of our knowledge, controlled side-by-side comparisons of monometallic CeO_2_-rich nanoparticles and compositionally complex, multi-rare-earth-containing oxide-rich nanoparticles prepared using a common plant-derived medium and harmonized processing conditions remain scarce. In the present study, three independent paired synthesis preparations were performed. For each independent preparation, a fresh aqueous extract was prepared from the same collected *M*. *oleifera* seed lot and divided between the corresponding CeO_2_ and multi-REO syntheses. This paired design reduced variability associated with extract preparation and post-synthesis processing and enabled composition-associated differences between the two nanoparticle systems to be evaluated within a common experimental framework. However, because the systems were prepared from inherently different precursor chemistries, the observed differences cannot be attributed exclusively to elemental composition.

The multi-REO material contained La, Ce, Nd, and Pr as the principal detected rare-earth elements, with Sm detected locally as a minor constituent in the elemental-mapping dataset. Complementary spectroscopic, diffraction, microscopic, elemental, and colloidal analyses were used to compare the apparent crystallographic ordering, morphology, micrometre-scale regional elemental distribution, surface functionality, optical-edge behavior, aggregation, hydrodynamic characteristics, and zeta potential of the two nanoparticle systems. Preliminary, assay-specific, cell-free screening was additionally used to compare their radical/reactive-species-scavenging, redox-related, protein-denaturation, and enzyme-inhibition responses. Overall, the study examines composition-associated physicochemical, optical, surface, colloidal, and screening-level differences within a common phytochemical synthesis environment without implying composition-only causation, confirmed rare-earth synergy, or therapeutic efficacy.

## 2. Materials and methods

### 2.1. Materials

All chemicals were of analytical grade unless otherwise specified. Light rare-earth oxide (LREO) cake concentrate (composition detailed in Anis *et al.*^11^ and results of ICP-OES are presented in SI Table S1) was used as the precursor for multi-REO NPs. Cerium(iii) chloride heptahydrate (CeCl_3_·7H_2_O, 99.9% purity) was purchased from Winlab. Sodium hydroxide (NaOH), nitric acid (HNO_3_) and ethanol were purchased from Sigma-Aldrich. Fresh *M. oleifera* seeds were collected in October 2025 from a private farm in Faqus city, Sharkia Governorate, Egypt. Deionized (DI) water was used throughout all experiments.

### 2.2. Preparation of *Moringa oleifera* seed extract

An aqueous extract of *M*. *oleifera* seeds was prepared and used as the common plant-derived medium for the synthesis of both nanoparticle systems. Briefly, the seeds were washed thoroughly, rinsed with deionized water, shade-dried, and then ground into a fine powder. Twenty grams of the seed powder were dispersed in 200 mL of deionized water and heated at 80 °C for 30 min under continuous stirring. After cooling to room temperature, the extract was filtered through Whatman No. 1 filter paper to remove insoluble residues. The resulting clear aqueous extract was kept at 4 °C and used as the biogenic complexing and stabilizing medium in the subsequent nanoparticle syntheses.

#### 2.2.1. Preliminary phytochemical screening

Preliminary phytochemical screening of the prepared *M. oleifera* seed extract was conducted following established qualitative methods^29,30^ to identify the presence of major classes of secondary metabolites, including flavonoids, tannins, saponins, and alkaloids.

#### 2.2.2. HPLC fingerprinting of *Moringa oleifera* seed extract

HPLC-DAD fingerprinting of the aqueous *M*. *oleifera* seed extract was performed using a Thermo Scientific UltiMate 3000 HPLC system equipped with a pump, an automated sample injector, and a DAD-3000 diode-array detector. Data were acquired and processed using Chromeleon 7 software. Chromatographic separation was performed on a Luna reversed-phase C18 column (150 × 4.6 mm, 5 µm) maintained at 35 °C. The mobile phase consisted of 0.05% (v/v) trifluoroacetic acid in acetonitrile and distilled water and was delivered under gradient-elution conditions. Samples, standards, and mobile phases were degassed and filtered through 0.45 µm membrane filters before analysis. Diode-array spectra were recorded over 230–400 nm, and chromatograms were extracted at 254 nm. Phenolic and related compounds were analyzed using a 10 µL injection volume at a flow rate of 0.8 mL min^−1^, whereas flavonoids were analyzed using a 20 µL injection volume at a flow rate of 1.0 mL min^−1^.

Individual analytes were assigned by comparing their retention times and UV absorption spectra with those of the corresponding authentic standards and were quantified using external calibration. The instrument-generated integration reports presented in Fig. S1 and S2 display analyte concentrations in ppm in the injected analytical solutions. As shown in Tables 2 and 3, the instrument-reported values were converted to sample-basis concentrations, expressed as µg g^−1^ of the analyzed sample, by accounting for the applicable sample-preparation and concentration factors. Consequently, the numerical values displayed in the supplementary integration reports differ from, but correspond to, the converted sample-basis concentrations reported in the main-text tables. The HPLC-DAD method was adapted from the method reported by Biswas *et al.*^31^ with minor modifications.

### 2.3. Synthesis of nanoparticles

For each independent preparation, a fresh aqueous extract was prepared from the same collected *M. oleifera* seed lot using the standardized extraction procedure described above. The freshly prepared extract was divided between the corresponding CeO_2_ and multi-REO syntheses. This paired preparation strategy maintained a common extract within each independent experimental preparation while incorporating independently prepared extracts across the three replicate synthesis batches. Accordingly, three independently prepared synthesis batches were obtained for each nanoparticle system.

The multi-REO synthesis was adapted from our previously reported *M. oleifera* leaf-extract-mediated protocol,^11^ with the seed extract used in the present study to provide a common plant-derived medium for the CeO_2_ and multi-REO systems. CeO_2_ NPs were prepared separately using CeCl_3_·7H_2_O as the monometallic cerium precursor. The corresponding CeO_2_ and multi-REO batches were prepared under harmonized reaction, purification, drying, storage, and dispersion conditions. This paired design reduced variability associated with extract preparation and post-synthesis processing and enabled composition-associated differences between the nanoparticle systems to be evaluated within a common experimental framework. However, because the two systems were prepared from inherently different precursor chemistries, the observed differences cannot be attributed exclusively to rare-earth composition. The overall preparation procedure is illustrated schematically in Fig. 1.

**Fig. 1:**
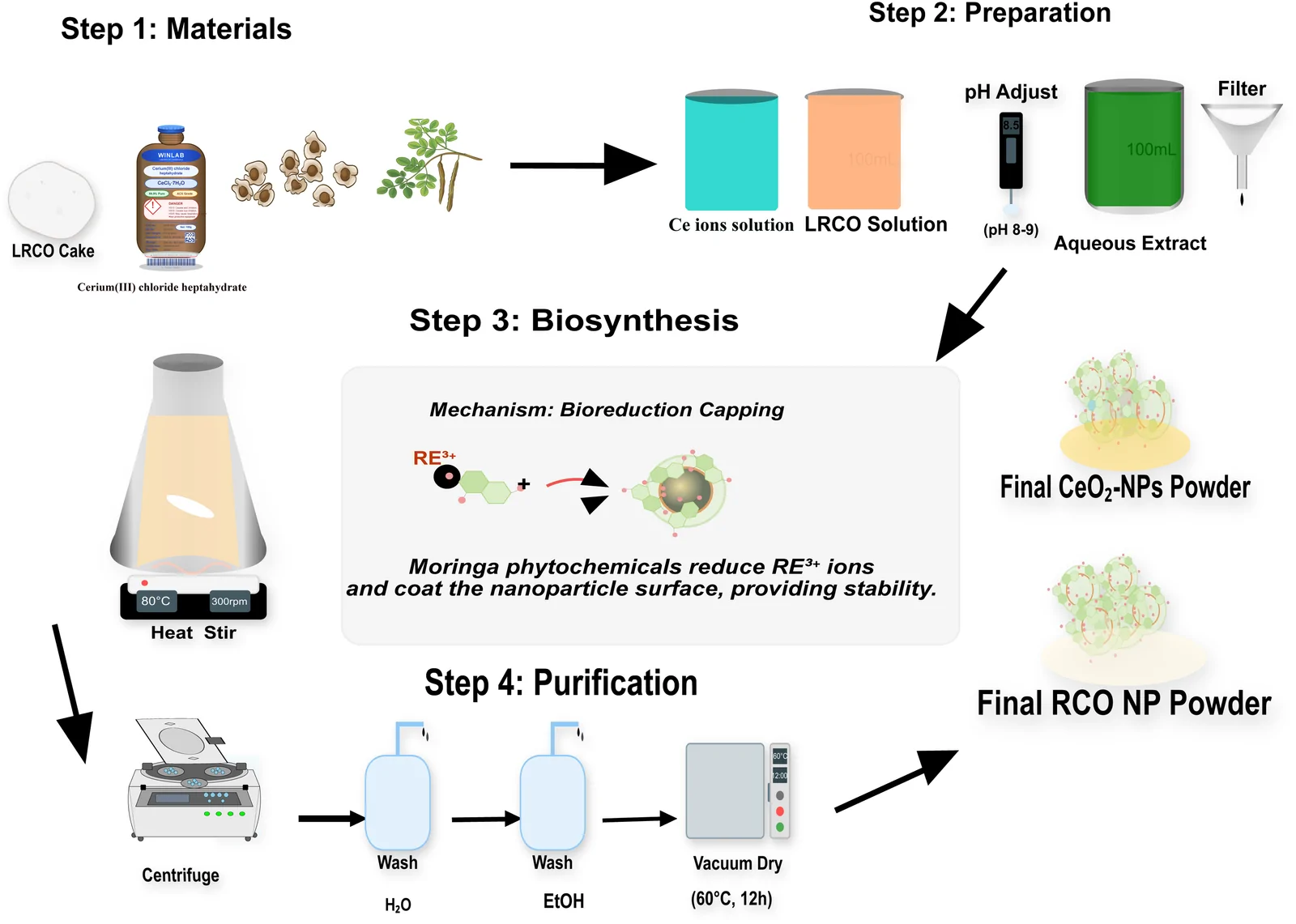
Schematic illustration of the green synthesis of CeO_2_ NPs and multi-REO NPs using *M. oleifera* seed extract. Adapted from *RSC Advances*, DOI: 10.1039/d5ra04558d.^11^

#### 2.3.1. Synthesis of multi-rare earth oxide nanoparticles (multi-REO NPs)

The multi-REO NPs were freshly prepared for this study using light rare-earth oxide (LREO) cake as the multi-element rare-earth precursor and *M. oleifera* seed extract as the biogenic complexing and stabilizing medium. The procedure was adapted from our previously optimized leaf-extract-based method; however, the leaf extract was replaced here with seed extract to maintain consistency with the CeO_2_ NP synthesis. Thus, the present method should be considered a seed-extract-adapted version of the earlier protocol rather than a direct repetition of the previously published leaf-extract synthesis.

Briefly, 1.0 g of LREO cake powder was dispersed in 100 mL of deionized water in an Erlenmeyer flask and stirred for 15 min to obtain a homogeneous suspension. The LREO suspension was mildly acidified with 1 M HNO_3_ under stirring to facilitate partial surface activation/dissolution of the oxide precursor. The pH was then adjusted to 8.0–9.0 using 1 M NaOH before addition of the seed extract. Subsequently, 50 mL of freshly prepared *M. oleifera* seed extract was added dropwise under continuous stirring. The mixture was maintained at 60 °C for 2 h with continuous stirring. During this reaction, interactions between rare-earth species and extract-derived biomolecules promoted the formation of a multi-component rare-earth-containing nanoparticulate precipitate. The obtained precipitate was collected and purified according to the common procedure described in Section 2.4.

#### 2.3.2. Synthesis of cerium oxide nanoparticles (CeO_2_ NPs)

CeO_2_ NPs were freshly synthesized for the present comparative study using CeCl_3_·7H_2_O as the monometallic rare-earth precursor and *M. oleifera* seed extract as the plant-derived complexing and stabilizing medium. A 0.1 M cerium precursor solution was prepared by dissolving 3.72 g of CeCl_3_·7H_2_O in 100 mL of deionized water under continuous magnetic stirring. Then, 50 mL of freshly prepared *M. oleifera* seed extract was added dropwise to the cerium solution while stirring was maintained, allowing gradual interaction between cerium species and extract-derived phytochemicals.

The pH of the reaction mixture was adjusted to 9.0 by the slow addition of 1 M NaOH. The alkaline medium was used to promote hydrolysis of cerium ions and the nucleation of cerium hydroxide/oxide species. The suspension was then heated at 60 °C for 2 h under vigorous stirring. A pale-yellow precipitate formed during the reaction, indicating the generation of a cerium-containing nanoparticulate product. The precipitate was collected and purified using the same washing, ethanol-rinsing, and drying procedure described in Section 2.4.

### 2.4. Purification of nanoparticles

The precipitates obtained from both syntheses were purified using the same protocol. Each precipitate was collected by centrifugation at 10 000 rpm for 15 min. The pellets were washed three times with deionized water by repeated resuspension and centrifugation, followed by a final wash with ethanol. The purified materials were dried in a hot-air oven at 80 °C for 6 h to obtain dry nanoparticle powders. The dried multi-REO NPs and CeO_2_ NPs were stored separately in desiccators until further physicochemical characterization and biological evaluation.

### 2.5. Characterization techniques

The purified powders of multi-REO NPs and CeO_2_ NPs were subjected to a series of characterization techniques to compare their optical, structural, morphological, compositional, and dispersion-related properties.

#### 2.5.1. UV-visible spectroscopy (UV-vis)

For optical analysis, each nanoparticle powder was dispersed in deionized water at 0.1 mg mL^−1^ using ultrasonication. UV-vis absorption spectra were recorded over the range of 200–800 nm using a JASCO V-530 double-beam spectrophotometer and quartz cuvettes with a 1 cm path length. Deionized water was used as the reference, and spectra were baseline-corrected before analysis. Each measurement was performed in triplicate, and the results are reported as mean ± SD. Optical band gaps were estimated from Tauc plots by extrapolating the linear region of (*αhν*)^2^*versus hν* to the energy axis, assuming a direct allowed transition. Urbach energies were calculated from the slope of ln(*α*) *versus hν* in the sub-bandgap region. OriginPro 2024 was used for Tauc and Urbach analyses.

#### 2.5.2. Fourier-transform infrared (FTIR) spectroscopy

To identify surface functional groups derived from the *Moringa* seed extract and those present on the synthesized nanoparticles, FTIR spectra were recorded on a Thermo Scientific Nicolet Apex FTIR spectrometer equipped with OMNIC Paradigm Software. Approximately 1 mg of each dried nanoparticle powder was mixed with 100–200 mg of spectroscopy-grade KBr, ground into a fine powder, and pressed into a semi-transparent pellet. Spectra were acquired in the range of 4000–400 cm^−1^.

#### 2.5.3. X-ray diffraction (XRD)

The diffraction characteristics of the powders were examined using a Malvern Panalytical Empyrean diffractometer equipped with Cu Kα radiation (*λ* = 1.5406 Å) and operated at 40 kV and 30 mA. The exported diffraction data covered a 2*θ* range of approximately 5–75°, from 5.015° to 74.975°, with a uniform data-point interval of 0.030°.

The experimental diffraction patterns were compared with reference patterns for chemically plausible crystalline phases. The expected positions of the principal reflections of cubic fluorite CeO_2_ were included as reference markers. Only reproducible features distinguishable from the local background were considered for assignment. Features that could not be resolved reliably from the local background or that lacked a unique and defensible reference match were left unindexed or unassigned. An isolated reflection was not considered sufficient for definitive phase identification.

Because the available diffraction features exhibited low signal-to-background ratios, substantial broadening, overlap, or uncertainty in phase assignment, defensible peak-width determination was not possible. Accordingly, Scherrer coherent-domain-size estimates were not retained.

#### 2.5.4. Electron microscopy (SEM/EDS and TEM)

Surface morphology and elemental composition were examined by SEM-EDS using a Thermo Scientific Prisma E scanning electron microscope operating at 30 kV. In addition to point EDS analysis, elemental mapping was performed at an accelerating voltage of 20 kV, to examine the spatial distribution of the constituent elements within the nanoparticle samples. Elemental maps of O, La, Ce, Nd, Pr, and Sm were acquired for the multi-REO nanoparticles, while Ce and O maps were obtained for the CeO_2_ nanoparticles.

For high-resolution imaging, particle size, and morphology, the samples were analyzed with a JEOL (JEM-2100 PLUS) TEM operated at an accelerating voltage of 200 kV. Samples were prepared by ultrasonicating a small amount of powder in ethanol, placing a single drop of the dilute suspension onto a carbon-coated copper grid, and allowing the solvent to evaporate.

#### 2.5.5. Dynamic light scattering (DLS) and zeta potential analysis

To evaluate hydrodynamic size distribution and dispersion-state behavior, the nanoparticle powders were re-dispersed in DI water at a concentration of 0.1 mg mL^−1^. The suspensions were analyzed using a Nicomp N3000 particle analyzer (Particle Sizing Systems, Inc., Santa Barbara, CA, USA) at a scattering angle of 90° and 25 °C. Zeta potential measurements were performed using a Zetasizer Nano instrument (Malvern Instruments Ltd., Malvern, UK) operated with Zetasizer software version 6.32. Measurements were conducted at approximately 25 °C in an aqueous medium using a clear disposable zeta cell. Hydrodynamic particle-size distributions were measured at a scattering angle of 90°, and zeta potentials were determined under the same dispersion condition.

### 2.6. Preliminary cell-free biochemical screening

A series of preliminary cell-free assays was performed to compare the biochemical responses of the biosynthesized CeO_2_ NPs and multi-REO NPs. The assessment included total antioxidant capacity, ferric-ion-reducing power, radical- and reactive-species-scavenging assays, AChE response, BSA thermal-denaturation inhibition, and α-amylase and α-glucosidase inhibition. These assays were used as assay-specific comparative screening models and were not intended to establish antioxidant, antidiabetic, anti-inflammatory, neuroprotective, or therapeutic efficacy.

For each nanoparticle system, the cell-free assay data were obtained from three independently prepared synthesis batches (*n* = 3 independent experimental units). Each synthesis batch was evaluated at 50, 100, and 200 µg mL^−1^, with batch identity maintained across the three concentrations. The corresponding reference compounds were evaluated in three independent assay runs, with each run including all three tested concentrations.

Where multiple measurements were obtained within a batch–concentration or run–concentration condition, these measurements were treated as technical replicates and averaged to obtain a single response value for the corresponding independent experimental unit. Technical replicates were not treated as independent observations and did not increase the inferential sample size.

For spectrophotometric assays involving nanoparticle suspensions, sample-specific blanks were included to correct for intrinsic absorbance, turbidity, and light-scattering effects. Reagent blanks, nanoparticle-only controls, enzyme-free or substrate-free blanks, and the corresponding positive controls were included where applicable. Blank-corrected absorbance values were used to calculate the reported assay responses.

#### 2.6.1. IC_50_ determination criteria

IC_50_ was defined operationally as the concentration associated with 50% inhibition or scavenging response under the specified assay conditions. IC_50_ estimation was based on concentration–response analysis, in which the measured responses were plotted against the logarithm of concentration and fitted using nonlinear dose–response models, as commonly recommended for IC_50_/EC_50_ determination.^32,33^ Concentration–response data were analyzed using GraphPad Prism version 10.6.1 and a constrained sigmoidal model, with the lower and upper response plateaus fixed at 0% and 100%, respectively, and the Hill slope fixed at 1. Responses measured at 50, 100, and 200 µg mL^−1^ were analyzed as described in the Statistical analysis section. IC_50_ values were reported as model-dependent estimates with profile-likelihood 95% confidence intervals.

An IC_50_ estimate and its confidence interval were reported only when the observed response reached or exceeded 50% within the tested concentration range of 50–200 µg mL^−1^ and the fitted model yielded an interpretable estimate. When 50% response was not reached within the tested range, the endpoint was designated as not reached within the tested range (NR), and no extrapolated numerical IC_50_ estimate or confidence interval was reported. Mathematical estimates generated outside the measured concentration range were excluded because they were not experimentally bracketed by the tested concentrations.

When the response lacked a meaningful monotonic concentration-dependent pattern and did not support interpretable sigmoidal fitting, the IC_50_ was reported as not determined (N.D.). This criterion was applied to the nanoparticle responses in the AChE assay. Because only three concentrations were evaluated, all fitted concentration–response curves, IC_50_ estimates, and associated confidence intervals were interpreted cautiously.

#### 2.6.2. Total antioxidant capacity and ferric-ion-reducing power

Total antioxidant capacity (TAC) was determined by the phosphomolybdenum method described by Prieto *et al.*^34^ and expressed as milligrams of gallic acid equivalent per gram of sample weight. The reducing power (ferric-ion-reducing power) was evaluated following Oyaizu,^35^ using ascorbic acid as a reference standard.

#### 2.6.3. Radical- and reactive-species-scavenging assays

DPPH radical scavenging activity was assessed according to Rahman *et al.*,^36^ using ascorbic acid as a positive control, and inhibition (%) and IC_50_ values were determined. ABTS˙^+^ radical scavenging activity was measured following Arnao *et al.*,^37^ and inhibition (%) and IC_50_ values were calculated. Nitric oxide (NO˙) scavenging activity was evaluated using the Griess-reagent method described by Marcocci *et al.*,^38^ and inhibition (%) and IC_50_ values were determined. Hydroxyl radical (HO˙) scavenging activity was determined using the deoxyribose degradation assay described by Halliwell and Gutteridge,^39^ in which HO˙-induced deoxyribose degradation was monitored colorimetrically. Hydrogen peroxide (H_2_O_2_) scavenging activity was measured according to Ruch *et al.*^40^ by monitoring the decrease in H_2_O_2_ in the presence of the sample; inhibition (%) and IC_50_ values were calculated.

#### 2.6.4. Preliminary cell-free enzyme- and protein-based assays

Selected enzyme- and protein-based assays were used to compare the cell-free responses of the biosynthesized nanoparticle systems. These included AChE response, BSA thermal-denaturation inhibition, and α-amylase and α-glucosidase inhibition. The assays were used as preliminary biochemical screening models and should not be interpreted as evidence of neuroprotective, anti-inflammatory, antidiabetic, or therapeutic efficacy.

##### 2.6.4.1 AChE inhibition assay

AChE response was evaluated using a modified Ellman method, with donepezil as the reference inhibitor.^41^ IC_50_ values were estimated only when a clear concentration-dependent response reached 50% inhibition within the tested range. For each assay, 5 µL of acetylthiocholine iodide (ATCh, 0.5 mM), 5 µL of 5,5′-dithiobis-2-nitrobenzoic acid (DTNB, 0.03 mM), and test samples at varying concentrations (50, 100 and 200 µg mL^−1^) were added to a flat-bottom 96-well microplate. The plate was pre-incubated for 10 min at 30 °C. The enzymatic reaction was initiated by the addition of 5 µL of AChE solution (0.3 U mL^−1^), and absorbance was immediately monitored at 412 nm using a microplate reader. A control experiment was performed containing all components except the test sample to account for background hydrolysis. The percentage inhibition of AChE activity was calculated using the formula: % Inhibition = [(*A*_0_ − *A*_1_)/*A*_0_] × 100, where *A*_0_ is the absorbance of the control and *A*_1_ is the absorbance in the presence of the test sample.

##### 2.6.4.2 BSA thermal-denaturation inhibition assay

The apparent inhibition of heat-induced BSA denaturation was evaluated as a preliminary cell-free protein-denaturation screening response. The assay was not intended to establish a specific protein-stabilization mechanism or anti-inflammatory or anti-arthritic efficacy.

The protein denaturation inhibition assay was performed using a modified heat-induced albumin denaturation method.^42^ A 5% (w/v) bovine serum albumin (BSA) solution was freshly prepared in distilled water. CeO_2_ NPs and multi-REO NPs were dispersed in distilled water, while diclofenac sodium was dissolved in distilled water at concentrations of 50, 100, and 200 µg mL^−1^.

For the assay, 0.45 mL of BSA solution was mixed with 0.05 mL of each test sample at the selected concentration. The control, representing maximum protein denaturation, consisted of 0.45 mL of BSA solution mixed with 0.05 mL of distilled water. Product control solutions were prepared by mixing 0.45 mL of distilled water with 0.05 mL of each test sample to correct for sample background. The pH of all reaction mixtures was adjusted to 6.3 using 1 N HCl. The tubes were incubated at 37 °C for 20 min, followed by heating at 57 °C for 3 min to induce protein denaturation. After cooling to room temperature, 2.5 mL of phosphate buffer (pH 6.4) was added to each tube, and the absorbance was measured at 416 nm using a UV-vis spectrophotometer against a phosphate buffer blank.

The percentage of protein denaturation inhibition was calculated using the following formula:Inhibition (%) = [(*A*_control_ − *A*_sample_)/*A*_control_] × 100where *A*_control_ represents the absorbance of the control and *A*_sample_ represents the absorbance in the presence of the test sample or reference inhibitor. Higher calculated percentages indicate a lower measured thermal-denaturation signal under the applied assay conditions.

##### 2.6.4.3 Carbohydrate-hydrolyzing enzyme inhibition assays

The inhibitory effects of the biosynthesized nanoparticles on carbohydrate-hydrolyzing enzymes were evaluated using α-amylase and α-glucosidase assays. α-Amylase and α-glucosidase inhibition assays were performed as preliminary cell-free models of carbohydrate-hydrolyzing enzyme inhibition. Acarbose, a clinically approved α-glucosidase inhibitor, was employed as the positive reference standard in both assays.

###### 2.6.4.3.1 α-Amylase inhibition assay

The α-amylase inhibitory activity was determined using a modified 3,5-dinitrosalicylic acid (DNSA) colorimetric method, which measures the formation of reducing sugars released during starch hydrolysis. This assay evaluates the ability of the test samples to inhibit pancreatic α-amylase activity. The assay measures the reduction in starch hydrolysis under the applied cell-free conditions.

A 0.02 M sodium phosphate buffer containing 0.006 M NaCl (pH 6.9) was prepared in distilled water. The α-amylase enzyme solution was prepared fresh by dissolving porcine pancreatic α-amylase in the same buffer at a concentration of 0.5 mg mL^−1^. The substrate solution consisted of 1% (w/v) soluble starch prepared in phosphate buffer, with gentle heating and stirring until complete dissolution. The DNSA reagent was prepared by dissolving 1 g of 3,5-dinitrosalicylic acid and 12 g of sodium potassium tartrate tetrahydrate in 20 mL of 2 M NaOH, followed by dilution with distilled water to a final volume of 40 mL. CeO_2_ NPs and multi-REO NPs were dispersed in the corresponding assay buffer, while acarbose was dissolved in the same buffer, at concentrations of 50, 100, and 200 µg mL^−1^.

For the assay, 0.5 mL of each test sample was mixed with 0.5 mL of α-amylase solution in test tubes and pre-incubated at room temperature (25 ± 2 °C) for 10 min to allow enzyme–inhibitor interaction. After pre-incubation, 200 µL of 1% starch solution was added to initiate the reaction, and the mixture was incubated at 37 °C for 30 min. The reaction was then terminated by adding 200 µL of DNSA reagent. The tubes were placed in a boiling water bath for 10 min to allow color development, and then cooled rapidly to room temperature in a cold-water bath. The reaction mixture was subsequently diluted with 5 mL of distilled water, and the absorbance was measured at 540 nm using a Shimadzu UV-2550 UV-vis spectrophotometer. A control containing the enzyme and substrate without an inhibitor was used to represent maximum enzyme activity (0% inhibition), while a blank without enzyme was used for background correction.

The percentage inhibition of α-amylase activity was calculated using the following equation:Inhibition (%) = [(*A*_control_ − *A*_sample_)/*A*_control_] × 100where *A*_control_ represents the absorbance of the control reaction corresponding to uninhibited enzyme activity, and *A*_sample_ represents the absorbance measured in the presence of the test sample or acarbose. Lower absorbance values indicate reduced starch hydrolysis due to enzyme inhibition.

###### 2.6.4.3.2 α-Glucosidase inhibition assay

The α-glucosidase inhibitory activity was evaluated using *p*-nitrophenyl-α-d-glucopyranoside (pNPG) as a chromogenic substrate, following an established colorimetric method. This assay measures the ability of the test samples to inhibit α-glucosidase activity and thereby reduce the hydrolysis of pNPG to *p*-nitrophenol, which can be quantified spectrophotometrically.

A 0.1 M potassium phosphate buffer (pH 6.8) was prepared from appropriate proportions of K_2_HPO_4_ and KH_2_PO_4_. The α-glucosidase enzyme solution was prepared in the same buffer at a concentration of 10 U mL^−1^ and stored at 4 °C until use. The substrate solution consisted of 10 mM *p*-nitrophenyl-α-d-glucopyranoside dissolved in potassium phosphate buffer. A 1 M sodium carbonate solution was prepared in distilled water and used to terminate the enzymatic reaction. CeO_2_ NPs and multi-REO NPs were dispersed in the corresponding assay buffer, while acarbose was dissolved in the same buffer, at concentrations of 50, 100, and 200 µg mL^−1^.

For the assay, 10 µL of each test sample was mixed with 5 µL of α-glucosidase solution in a 96-well microplate and pre-incubated at 37 °C for 20 min to allow enzyme–inhibitor interaction. The reaction was then initiated by adding 10 µL of pNPG substrate solution, followed by incubation at 37 °C for 30 min. During this period, α-glucosidase catalyzed the hydrolysis of pNPG, releasing *p*-nitrophenol. The reaction was terminated by adding sodium carbonate solution, and the absorbance was measured at 410 nm using a Shimadzu UV-2550 UV-vis spectrophotometer. A control containing the enzyme and substrate without an inhibitor was included to represent 0% inhibition, while a blank without enzyme was used for background correction.

The percentage inhibition of α-glucosidase activity was calculated according to the following equation:Inhibition (%) = [(*A*_control_ − *A*_sample_)/*A*_control_] × 100where *A*_control_ is the absorbance of the control reaction corresponding to maximum *p*-nitrophenol release, and *A*_sample_ is the absorbance measured in the presence of the test sample or acarbose. Lower absorbance indicates reduced substrate hydrolysis due to enzyme inhibition.

### 2.7. Statistical analysis

For each nanoparticle system, three independent synthesis batches were prepared using three freshly and independently prepared aqueous extracts obtained from the same collected *M. oleifera* seed lot (*n* = 3 independent experimental units). Within each independent preparation, the same freshly prepared extract was divided between the corresponding CeO_2_ and multi-REO syntheses. After division of the extract, the two nanoparticle systems were synthesized and processed separately, and each resulting nanoparticle synthesis batch was treated as a distinct treatment-specific experimental unit in the primary analysis. Each independent synthesis batch was evaluated at 50, 100, and 200 µg mL^−1^, with experimental-unit identity maintained across the three concentrations. The corresponding reference compounds were evaluated in three independent assay runs, with each run including all three tested concentrations. Thus, each treatment was represented by three independent experimental units (*n* = 3).

Where multiple measurements were obtained within a batch-concentration or run-concentration condition, these measurements were treated as technical replicates and averaged to obtain a single response value for the corresponding independent experimental unit. Technical replicates were not treated as independent observations and did not increase the inferential sample size. Unless otherwise stated, data are presented as mean ± SD across the three independent experimental units.

Treatment was analyzed as the between-unit factor, whereas concentration was analyzed as the within-unit repeated factor. The effects of treatment, concentration, and the treatment × concentration interaction were evaluated using two-way mixed-design repeated-measures analysis of variance (ANOVA). Sphericity was not assumed, and the Geisser–Greenhouse correction was applied to the concentration and treatment × concentration effects. Comparisons among treatments at each concentration were performed using Tukey-adjusted multiple-comparison procedures, with a separate family of comparisons defined for each concentration. Statistical significance was set at *P* < 0.05.

Concentration–response data were analyzed using GraphPad Prism version 10.6.1 and a constrained sigmoidal model, with the lower and upper response plateaus fixed at 0% and 100%, respectively, and the Hill slope fixed at 1. IC_50_ values were reported as model-dependent estimates with profile-likelihood 95% confidence intervals.

An IC_50_ estimate and its confidence interval were reported only when the observed response reached or exceeded 50% within the tested concentration range of 50–200 µg mL^−1^ and the fitted model yielded an interpretable estimate. When 50% response was not reached within the tested range, the endpoint was designated as not reached within the tested range (NR), and no extrapolated numerical IC_50_ estimate or confidence interval was reported. Although nonlinear regression could generate mathematical estimates outside the measured concentration range, such extrapolated values were excluded because they were not experimentally bracketed by the tested concentrations.

When the response lacked a meaningful monotonic concentration-dependent pattern and did not support interpretable sigmoidal fitting, the IC_50_ was reported as not determined (N.D.). This criterion was applied, for example, to the nanoparticle responses in the AChE assay. Because only three concentrations were evaluated, all fitted concentration–response curves, IC_50_ estimates, and associated confidence intervals were interpreted cautiously.

## 3. Results and discussion

The present study compared two rare-earth-containing nanoparticle systems prepared within a paired *M. oleifera* seed-extract-mediated framework: CeO_2_-rich nanoparticles derived from CeCl_3_·7H_2_O and Ce-rich, multi-rare-earth-containing oxide-rich nanoparticles derived from light rare-earth oxide cake. The comparative design enabled differences associated with precursor chemistry and rare-earth compositional complexity to be examined under standardized extraction and harmonized reaction and post-synthesis conditions. However, the design did not isolate elemental composition as the sole experimental variable because the systems were prepared from chemically distinct precursor materials.

The results are discussed according to the experimental sequence of the study: extract phytochemical characteristics, optical responses, surface chemistry, diffraction behavior, morphology and elemental composition, hydrodynamic and electrokinetic properties, and preliminary cell-free screening responses. Mechanistic interpretations are presented cautiously and are distinguished from directly observed findings.

### 3.1. Phytochemical characterization of *Moringa oleifera* seed extract

The aqueous *M. oleifera* seed extract was used as the common biogenic medium for both nanoparticle syntheses. Qualitative phytochemical screening of aqueous, methanolic, and petroleum extracts indicated the presence of several metabolite classes, including alkaloids, tannins, phenolic compounds, flavonoids, steroids/saponins, and terpenoids, with clear solvent-dependent differences in extractability (Table 1).

**Table 1:** Qualitative phytochemical profile of *M. oleifera* seed extracts prepared using different solvents^a^

Plant constituent	Aqueous extract	Methanolic extract	Petroleum ether extract
Alkaloids	+	−	−
Tannins	+	+	−
Phenols	+	+	−
Flavonoids	+	+	−
Steroids/Saponins	+	−	+

a (+) indicates detected; (−) indicates not detected under the applied qualitative screening conditions.

The screening results indicated a solvent-dependent distribution of secondary metabolites. The aqueous extract contained alkaloids, tannins, phenolic compounds, flavonoids, steroids/saponins, and terpenoids, whereas the methanolic extract showed positive responses for tannins, phenols, flavonoids, and terpenoids. In contrast, the petroleum ether extract mainly showed the presence of steroids/saponins, reflecting the preferential extraction of less polar constituents. This positive response likely reflects steroid-associated constituents rather than true saponin enrichment, because saponins are amphiphilic glycosides with hydrophilic sugar moieties linked to lipophilic aglycones,^43^ whereas plant sterols are commonly treated as lipid-associated phytochemicals.^44^

The applied phytochemical screening tests are class-specific with “qualitative” results and should not be viewed as proof of nucleation pathways. Rather, the results indicate that *M. oleifera* seed extract is a complex assemblage of biomolecules, including phenolics, flavonoids, tannins, alkaloids, saponin or steroid-like compounds, terpenoids, and sulfur-bearing glucosinolate-related compounds and their breakdown products.^45,46^ These constituents can contribute to the formation of nanoparticles through complementary functional groups for rare-earth ion complexation, hydrolysis-assisted nucleation, surface adsorption, as well as surface association and regulation of developing nanoparticle growth.^24,47,48^ The S and P signals may reflect precursor-associated species, extract-derived surface constituents, residual processing species, or support-related contributions. Their precise chemical forms and origins cannot be established by EDS or the present HPLC-DAD analysis. The precise chemical forms and origins of these signals remain unresolved and would require targeted chromatographic and/or surface-sensitive spectroscopic analyses. Overall, the constituents of the *M. oleifera* seed extract may participate in rare-earth-ion coordination, particle-growth regulation, and surface association; however, more specific mechanistic assignments require direct experimental validation.

#### 3.1.1. HPLC profiling of *M. oleifera* seed extract

The HPLC-DAD phytochemical profile of the *M. oleifera* seed extract was obtained. Targeted reversed-phase HPLC-DAD profiling identified and quantified selected phenolic and flavonoid constituents in the aqueous *M. oleifera* seed extract used as the biogenic synthesis medium (Tables 2 and 3). Ten phenolic and related compounds were detected. Pyrocatechol was the most abundant (120.29 µg g^−1^), followed by quinic acid (72.11 µg g^−1^), resorcinol (38.95 µg g^−1^), chlorogenic acid (20.82 µg g^−1^), and gallic acid (13.39 µg g^−1^); cinnamic acid (8.71 µg g^−1^), ferulic acid (6.43 µg g^−1^), and vanillic acid (2.57 µg g^−1^) were present at lower levels, whereas coumaric and ellagic acids were detected at trace calculated concentrations of 0.06 µg g^−1^ each (Fig. S1).

**Table 2:** Quantification of phenolic acids and related compounds in the *M. oleifera* seed extract by HPLC-DAD^a^

No.	Compound	Retention time (min)	Concentration (µg g^−1^)	Relative peak area (%)
1	Quinic acid	1.983	72.11	35.51
2	Gallic acid	2.643	13.39	9.03
3	Chlorogenic acid	3.937	20.82	7.17
4	Resorcinol	4.737	38.95	10.26
5	Pyrocatechol	5.243	120.29	11.87
6	Vanillic acid	5.943	2.57	4.87
7	Coumaric acid	7.850	0.06	0.11
8	Ellagic acid	9.093	0.06	0.12
9	Ferulic acid	10.903	6.43	8.22
10	Cinnamic acid	14.130	8.71	12.85

a Values are expressed as µg g^−1^ of the analyzed sample; retention times and relative peak areas are from the HPLC-DAD integration report (254 nm). Coumaric and ellagic acids were detected at trace calculated concentrations under the applied analytical conditions.

**Table 3:** Quantification of flavonoids in the *M. oleifera* seed extract by HPLC-DAD^a^

No.	Compound	Retention time (min)	Concentration (µg g^−1^)	Relative peak area (%)
1	Apigenin	2.607	6739.43	98.11
2	Diosmin	4.147	0.16	1.07
3	Rutin	4.783	1.21	0.55
4	Hesperidin	7.700	0.02	0.01
5	Kaempferol	12.043	0.03	0.10
6	Quercetin	13.440	0.65	0.17

a Values are expressed as µg g^−1^ of the analyzed sample. The peak assigned to apigenin (2.607 min) accounted for 98.11% of the integrated flavonoid peak area. Minor flavonoids were quantified against their authentic standards.

In the targeted flavonoid fraction, the chromatogram was dominated by an early-eluting peak (2.607 min) assigned to apigenin on the basis of retention-time and UV-spectral matching with the authentic standard; this peak accounted for 98.11% of the integrated flavonoid peak area and corresponded to a calculated concentration of 6739.43 µg g^−1^. The remaining flavonoids were detected at markedly lower calculated concentrations: rutin (1.21 µg g^−1^), quercetin (0.65 µg g^−1^), diosmin (0.16 µg g^−1^), kaempferol (0.03 µg g^−1^), and hesperidin (0.02 µg g^−1^) (Fig. S2). Because the assignment was not confirmed by standard addition, peak-purity analysis, or mass spectrometry, the identity and calculated concentration of this dominant peak should be interpreted cautiously.

The assigned constituents contain hydroxyl, carboxyl, and carbonyl functionalities that can participate in rare-earth-ion coordination, hydrolysis-assisted nucleation, nanoparticle growth, and surface stabilization, thereby complementing the qualitative phytochemical screening and FTIR data by confirming the chemical complexity of the aqueous synthesis medium. Nevertheless, chromatographic detection alone does not establish the individual contribution of each compound to nanoparticle nucleation, growth, capping, or the measured cell-free screening responses; the data should therefore be interpreted as a targeted phytochemical profile based on the available reference standards rather than a comprehensive characterization of the complete extract metabolome.

Similar phytochemical-assisted synthesis mechanisms have been reported for other green-synthesized oxide nanomaterials. For example, in pomegranate-husk-mediated chromium oxide nanoparticles, phenolic and flavonoid constituents were proposed to participate in metal-ion complexation, nucleation, and stabilization, supporting the hypothesis that the phytochemicals identified in *M. oleifera* extract may play analogous roles during CeO_2_ and multi-REO nanoparticle formation.^15^ The possible roles of these constituents in rare-earth-ion coordination, nanoparticle formation, and surface interactions are further discussed in Section 3.3.

### 3.2. FTIR analysis of the nanoparticles

Fourier-transform infrared spectroscopy was used to examine the surface-associated functional groups and low-wavenumber metal–oxygen-related vibrational features of the biosynthesized CeO_2_-rich and multi-REO nanoparticle samples over the range of 4000–400 cm^−1^ (Fig. 2).

**Fig. 2:**
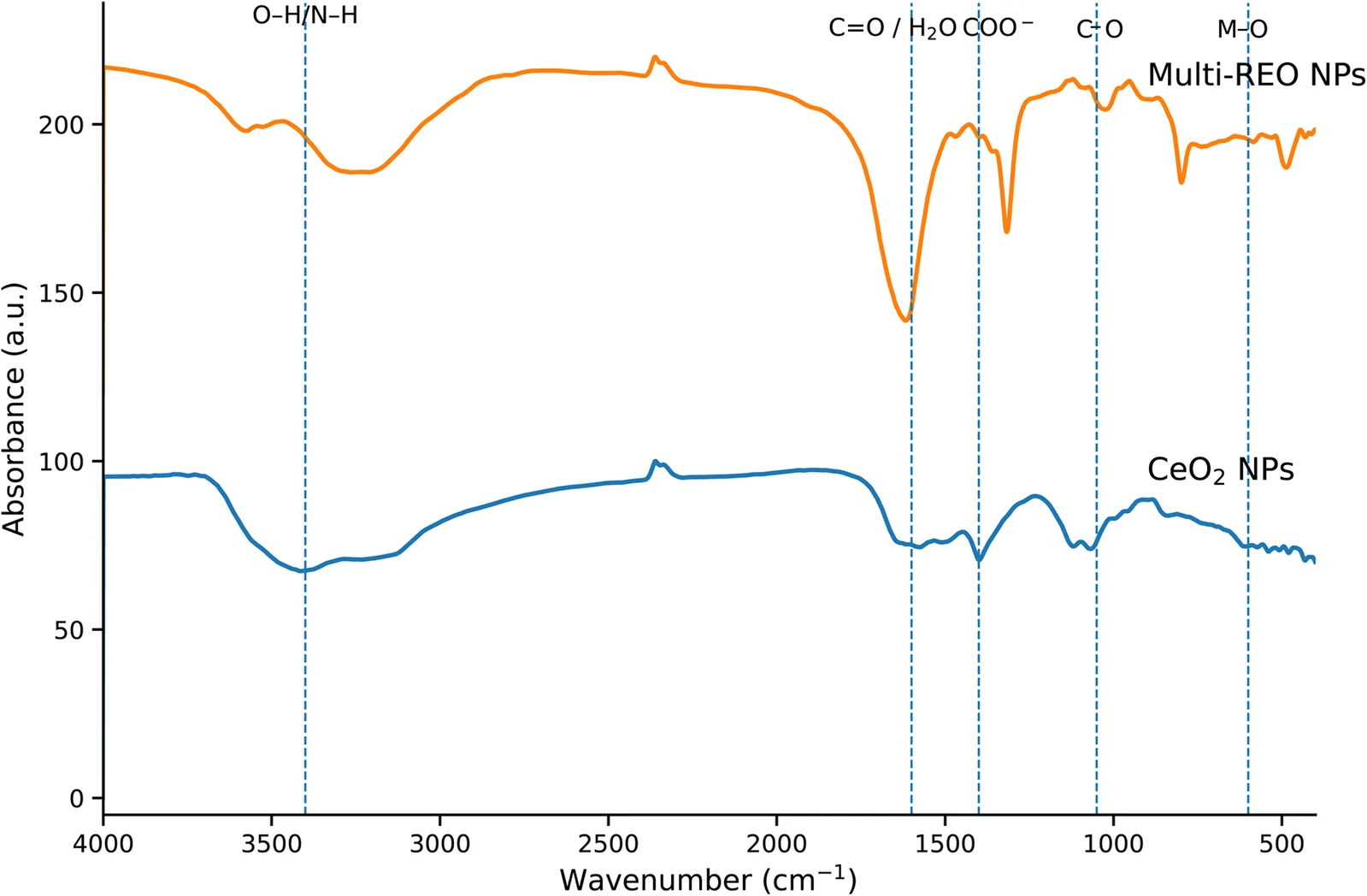
FTIR spectra of biosynthesized CeO_2_ NPs and multi-REO NPs recorded over the range of 4000–400 cm^−1^. The spectra are vertically offset for clarity. Annotated bands correspond to extract-derived surface functional groups, including O–H, C=O/amide, COO^−^, and C–O vibrations, together with low-wavenumber metal–oxygen vibrational features.

#### 3.2.1. Functional group analysis *via* FTIR spectroscopy

The spectra exhibited several shared features together with differences in the hydroxyl-, carbonyl/amide-, carboxylate-, C–O-, and low-wavenumber spectral regions. These features were interpreted qualitatively to assess the possible involvement of extract-derived functionalities in rare-earth-ion coordination and surface association.

For CeO_2_ NPs, prominent absorption bands were observed at 3413.5 cm^−1^, assigned to O–H stretching vibrations from hydroxyl groups and adsorbed water; 2277.6 cm^−1^, a weak band of uncertain assignment that may originate from surface carbonate species, an organic overtone, or extract-derived residues; 1575.6 cm^−1^, associated with C=O/amide-related vibrations; 1398.2 cm^−1^, consistent with symmetric COO^−^ stretching from carboxylate groups; and 1068.4 cm^−1^, attributed to C–O stretching from carbohydrate- or alcohol-related groups. The broad low-wavenumber absorption envelope below 600 cm^−1^ supports the presence of cerium–oxygen bonding in the CeO_2_-containing sample.

The multi-REO NPs displayed a distinct spectral profile with O–H stretching bands at 3575.5 and 3267.0 cm^−1^, suggesting different hydroxyl environments and hydrogen-bonding interactions compared with CeO_2_ NPs. Additional bands at 1618.1, 1317.2, and 1024.1 cm^−1^ are consistent with extract-derived carbonyl/carboxylate and C–O-containing surface functionalities. The low-wavenumber features observed at approximately 798.4, 586.3, 487.9, and 430.1 cm^−1^ were consistent with contributions from metal–oxygen and other surface-related vibrational environments in the compositionally complex multi-REO material. However, FTIR alone cannot distinguish individual La–O, Ce–O, Nd–O, Pr–O, or Sm–O contributions or establish the identities and proportions of the constituent phases.

The FTIR results support the presence of extract-derived surface functionalities and metal–oxygen bonding in both nanoparticle systems. Hydroxyl, carboxylate/amide, and C–O containing groups may contribute to rare-earth ion complexation, surface adsorption, and stabilization during nanoparticle formation. The broader and more complex hydroxyl- and carboxylate-related features of the multi-REO sample were consistent with differences in surface-associated functionalities and local coordination environments relative to the CeO_2_-rich material. However, contributions from adsorbed water, residual organic constituents, band overlap, and differences in sample preparation cannot be excluded. Nevertheless, these interpretations should be considered supportive rather than definitive, and phase-level structural assignment should be interpreted together with XRD, SAED, and complementary surface-sensitive analyses.

### 3.3. Proposed extract-mediated formation and surface-functionalization mechanism

The qualitative phytochemical-screening, targeted HPLC-DAD, and FTIR findings collectively indicate that the aqueous *M. oleifera* seed extract contains diverse oxygen- and nitrogen-containing constituents capable of interacting with rare-earth ions. Targeted HPLC-DAD profiling confirmed the presence of selected phenolic and flavonoid compounds, while the qualitative screening indicated additional classes of biomolecules, including tannins, alkaloids, terpenoids, and protein- or saponin/steroid-related constituents. Unlike the conventional phytogenic synthesis of noble-metal nanoparticles, the principal role of the extract in the present rare-earth systems is not interpreted as reduction of the metal ions to zero-valent states. Instead, hydroxyl-, carbonyl-, carboxylate-, amide-, and amine-containing constituents are proposed to participate mainly in metal-ion coordination, regulation of alkaline hydrolysis, nucleation, particle-growth control, and surface functionalization.

During the initial stage, rare-earth ions may coordinate with phenolic, flavonoid, protein-derived, and other extract constituents through oxygen- and nitrogen-donor sites. Under the alkaline synthesis conditions, coordinated metal ions undergo hydrolysis, producing hydroxo and oxo-hydroxo species. Further condensation through metal–O–metal linkages promotes nucleation and growth of oxygen-containing rare-earth domains. Dehydration and oxidation during the reaction and subsequent drying steps may then produce the observed oxide-rich nanoparticulate materials. Extract-derived biomolecules may remain adsorbed on the developing particle surfaces and contribute to growth regulation, surface functionalization, and partial steric stabilization.

For the CeO_2_-rich system, Ce^3+^-containing precursor species are proposed to undergo ligand-assisted hydrolysis and nucleation, followed by condensation, dehydration, and oxidative conversion toward a Ce(iv)-rich oxide phase. This pathway is chemically consistent with the formation of ceria from a Ce^3+^ precursor under alkaline and oxidative processing conditions. However, the Ce^3+^/Ce^4+^ ratio, oxygen-vacancy concentration, and detailed oxidation pathway were not directly determined in the present study; therefore, the proposed pathway should be regarded as a mechanistic interpretation rather than a definitive oxidation-state assignment.

For the multi-REO system, La-, Ce-, Nd-, and Pr-containing precursor species, together with the minor Sm constituent detected by SEM-EDS, are proposed to form phytochemical–metal-ion complexes before undergoing alkaline hydrolysis. Subsequent formation of hydroxo/oxo-hydroxo species, nucleation, oxo-bridged condensation, and dehydration may generate the observed multi-rare-earth-containing oxide-rich product. Because the available XRD and SEM-EDS results do not establish atomic-scale elemental mixing or definitive phase identity, this pathway should not be interpreted as evidence of a single-phase solid solution. The resulting material may instead comprise a multiphase oxide system, an oxide/oxyhydroxide-rich composite, and/or physically associated rare-earth-containing domains.

The proposed surface-functionalization mechanism is supported by changes in the FTIR regions associated with O–H, carbonyl/carboxylate, and amide-containing groups. The observed band shifts, broadening, and intensity changes are consistent with the involvement of hydroxyl-, carboxylate-, carbonyl-, and protein-derived functionalities in rare-earth-ion coordination and nanoparticle surface interactions. The broader and more complex hydroxyl/carboxylate spectral features of the multi-REO sample may reflect a more heterogeneous surface-coordination environment, although contributions from adsorbed water and residual organic species cannot be excluded.

Proteins, amino acids, and other extract-derived biomolecules may additionally contribute to steric capping and particle-growth regulation. Nevertheless, the surface-associated organic layer did not completely prevent aggregation, particularly in the multi-REO suspension. This interpretation is consistent with the low zeta-potential magnitude of the multi-REO NPs (−5.09 mV), compared with the more negative value measured for CeO_2_ NPs (−25.5 mV), and with the broad hydrodynamic-size distributions observed by DLS. Therefore, the extract-derived layer is interpreted as contributing to surface functionalization and partial steric stabilization rather than complete colloidal stabilization.

The proposed extract-mediated formation pathways are summarized schematically in Fig. 3. The schematic integrates the phytochemical-screening, HPLC-DAD, FTIR, SEM-EDS, DLS, and zeta-potential findings and is presented as an evidence-supported mechanistic model rather than direct molecular-level proof.

**Fig. 3:**
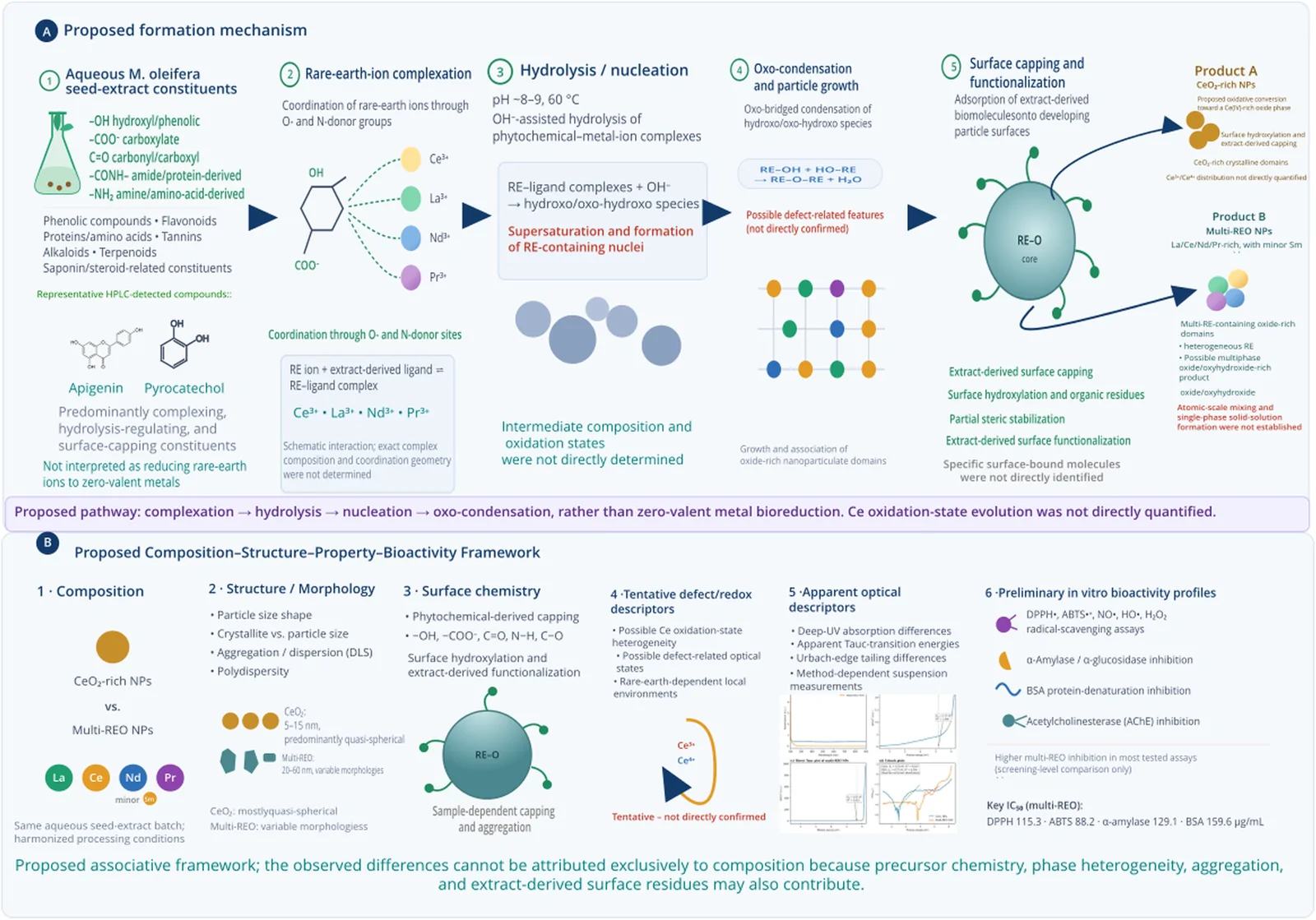
Proposed extract-mediated formation mechanism and composition–structure–property–screening-response framework for CeO_2_-rich and multi-REO nanoparticles synthesized using aqueous *M. oleifera* seed extract. (A) Extract-derived phenolic, flavonoid, protein, amino-acid, and other oxygen- and nitrogen-containing constituents are proposed to coordinate rare-earth precursor ions through O- and N-donor groups. At pH 8–9 and 60 °C, the resulting complexes undergo OH^−^-assisted hydrolysis and formation of hydroxo/oxo-hydroxo species, followed by nucleation, oxo-bridged condensation, particle growth, and extract-derived surface association. The Ce-containing pathway is proposed to yield CeO_2_-rich nanoparticles through oxidative evolution toward a Ce(iv)-rich oxide phase, whereas the multi-REO pathway yields a compositionally complex, multi-rare-earth-containing oxide-rich product comprising La, Ce, Nd, and Pr as the principal detected rare-earth elements, with Sm detected locally as a minor constituent. (B) Proposed associative framework linking compositional complexity with differences in morphology, surface and colloidal chemistry, tentative defect/redox descriptors, apparent optical characteristics, and preliminary cell-free screening-response profiles. The schematic represents an evidence-supported mechanistic and interpretive model; it does not independently establish the identities of surface-bound biomolecules, individual oxidation-state distributions, oxygen-vacancy concentrations, atomic-scale elemental mixing, single-phase solid-solution formation, or a direct causal relationship between composition and cell-free screening responses.

### 3.4. Optical properties of the biosynthesized nanoparticles

The optical responses of the biosynthesized nanoparticle systems were initially assessed by visual observation. Following synthesis, the clear precursor solutions changed to pale-yellow CeO_2_ NP dispersions and off-white multi-REO NP dispersions. These color changes were consistent with changes in the chemical and dispersion states of the precursor systems but were not considered independent evidence of nanoparticle formation. Nanoparticle formation was instead evaluated using the combined spectroscopic, structural, and microscopic findings.

The UV-vis spectra of the CeO_2_ NP and multi-REO NP suspensions exhibited strong absorption in the deep-UV region, with maxima near 200 nm and greater measured absorbance for the multi-REO suspension (Fig. 4a). The difference in absorbance may reflect combined effects of composition, particle concentration, aggregation, light scattering, and electronic absorption. Accordingly, the greater absorbance of the multi-REO suspension cannot be assigned specifically to defect-mediated transitions from the present UV-vis data alone.^49,50^

**Fig. 4:**
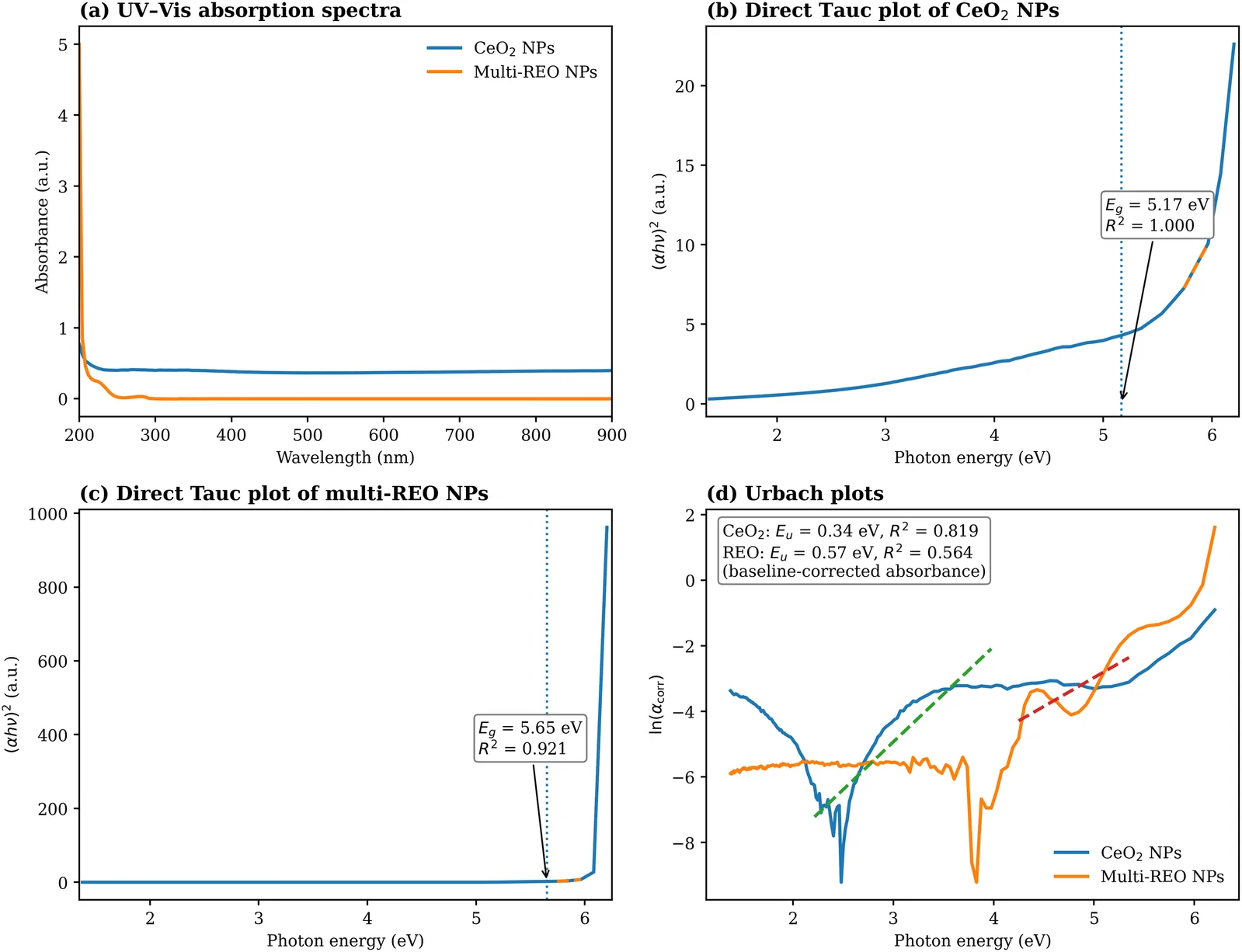
Optical characterization of the biosynthesized nanoparticle systems: (a) UV-vis spectra of the CeO_2_ NP and multi-REO NP suspensions; (b) direct-transition Tauc representation of CeO_2_ NPs, yielding an apparent transition energy of approximately 5.17 eV; (c) direct-transition Tauc representation of multi-REO NPs, yielding an apparent transition energy of approximately 5.65 eV; and (d) Urbach plots derived from the baseline-corrected optical data, yielding apparent Urbach energies of 0.34 eV for CeO_2_ NPs and 0.57 eV for multi-REO NPs. Because the measurements were performed on nanoparticle suspensions and the transition type was not independently established, the derived values are interpreted as method-dependent apparent optical descriptors.

Apparent optical-transition energies were estimated using a direct-transition Tauc representation by plotting (*αhν*)^2^ against photon energy (*hν*). Linear extrapolation yielded apparent direct-transition energies of approximately 5.17 eV for CeO_2_ NPs and 5.65 eV for multi-REO NPs (Fig. 4b and c). These values should be interpreted cautiously because the direct-transition model was applied as an operational fitting approach and the transition character was not independently established. The apparent value obtained for CeO_2_ NPs is also higher than many values commonly reported for nanoceria, which vary according to synthesis conditions, particle size, stoichiometry, measurement configuration, and fitting procedure.

The lower apparent transition energy of CeO_2_ NPs may be associated with band-tail states and defect- or oxidation-state-related features commonly reported for nanoceria. However, the Ce^3+^/Ce^4+^ distribution and oxygen-vacancy concentration were not directly determined in the present sample. The higher apparent transition energy of the multi-REO NPs may reflect the combined optical response of multiple rare-earth-containing oxide-rich phases and heterogeneous local electronic environments; however, the contribution of individual phases cannot be resolved from the present UV-vis data alone.^51,52^

Urbach analysis was used to describe absorption-edge tailing (Fig. 4d), yielding apparent Urbach energies of 0.34 eV for CeO_2_ NPs and 0.57 eV for multi-REO NPs. The higher value obtained for the multi-REO sample indicates broader absorption-edge tailing under the applied analysis and may reflect compositional complexity, heterogeneous local coordination environments, surface-associated constituents, aggregation, and defect-state distributions rather than reduced crystallinity alone. Urbach-edge behavior in CeO_2_-based materials may also be influenced by lattice strain, surface and grain-boundary features, and oxidation-state-related electronic states; however, these contributions cannot be distinguished individually using the present optical measurements.^53,54^

Because the measurements were performed on nanoparticle suspensions, the apparent Tauc-transition and Urbach energies may additionally be influenced by light scattering, particle aggregation, concentration, baseline correction, optical path length, and extract-derived surface-associated constituents. The calculated values should therefore be regarded as method-dependent apparent optical descriptors. They support differences in absorption-edge behavior between the two nanoparticle systems but do not independently establish specific defect structures, oxygen vacancies, Ce^3+^/Ce^4+^ distributions, or phase-specific electronic transitions.^55,56^

The apparent direct-transition energy obtained for the CeO_2_ NP suspension (5.17 eV) is higher than many values commonly reported or assigned to the optical absorption edge of CeO_2_-based materials. The interpretation of this edge is not straightforward because the commonly assigned value near 3.1 eV involves excitation into localized Ce 4f states rather than a conventional delocalized conduction band, whereas combined steady-state and ultrafast transient-absorption measurements have supported an optical-gap value closer to 4.0 eV. These differences illustrate the sensitivity of the derived optical parameter to the electronic-transition model, measurement configuration, sample characteristics, and analytical procedure.^52,57^ The value obtained in the present study is therefore interpreted as a suspension- and model-dependent apparent transition energy rather than an intrinsic band gap.

The still higher apparent transition energy obtained for the multi-REO suspension (5.65 eV) may reflect combined contributions from compositional and phase complexity, heterogeneous local coordination environments, morphology, aggregation, light scattering, and surface-associated constituents. However, the individual contributions of the rare-earth-containing components cannot be resolved using the present suspension UV-vis data, and no phase-specific electronic-transition assignment is made.

### 3.5. Structural and crystallographic characterization

The XRD pattern of the CeO_2_ NPs was characterized by a broad background and low-intensity, poorly resolved diffraction features, indicating limited long-range crystallographic ordering. The expected positions of the principal reflections of cubic fluorite CeO_2_, corresponding to the (111), (200), (220), and (311) planes, are included in Fig. 5 as reference markers. However, several experimental features were close to the local background and were not resolved sufficiently for reliable Miller-index assignment. Accordingly, these markers represent expected cubic CeO_2_ reference positions rather than definitive indexing of all experimental features, and weak or ambiguous features were left unindexed. Because the available CeO_2_ features did not permit defensible peak-width determination, no Scherrer coherent-domain-size estimate was reported.

**Fig. 5:**
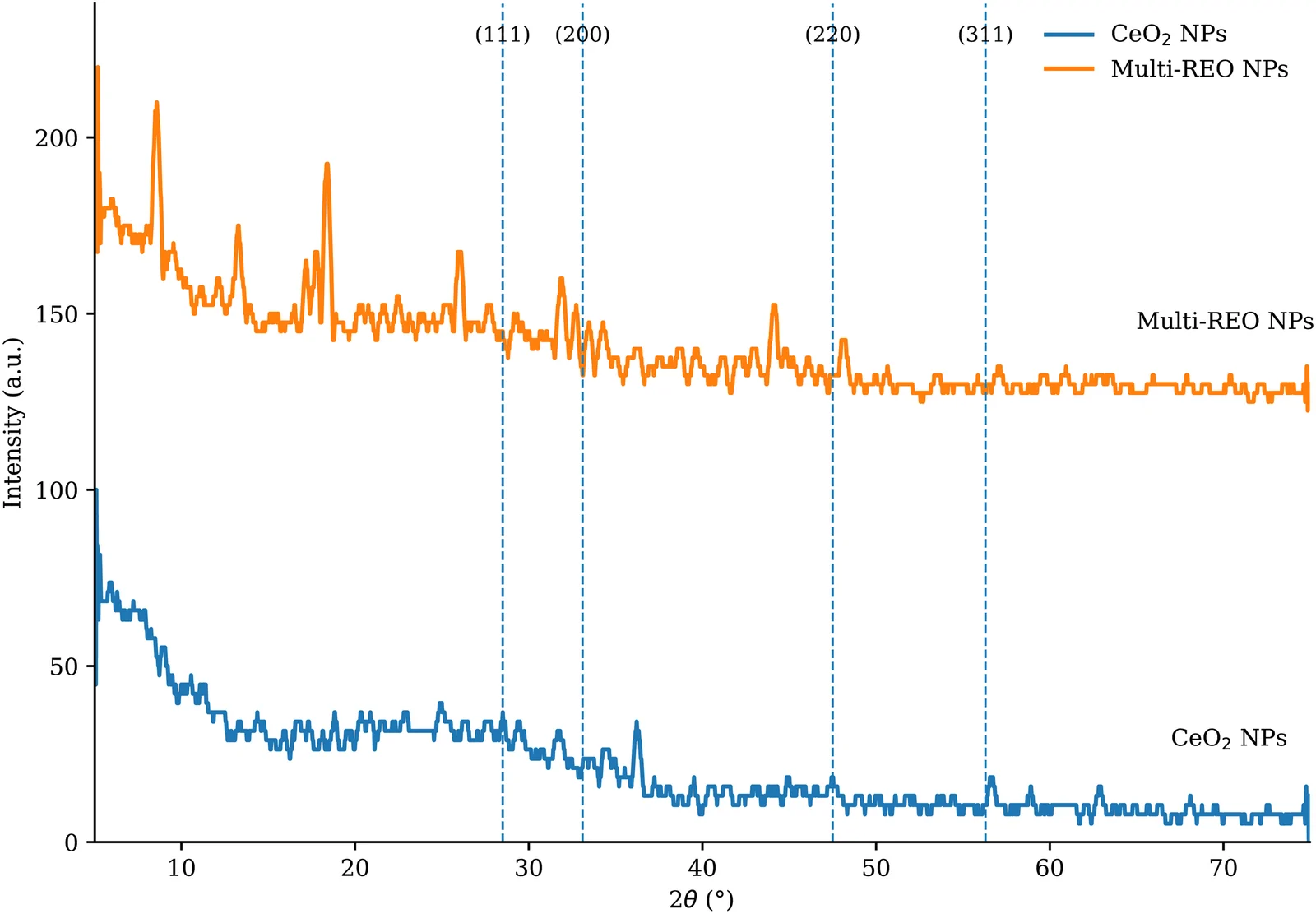
X-ray diffraction patterns of the biosynthesized CeO_2_ NPs and multi-REO NPs. The patterns are vertically offset for clarity. Dashed markers indicate the expected positions of the principal reflections of cubic fluorite CeO_2_ at 2*θ* ≈ 28.5°, 33.1°, 47.5°, and 56.3°, corresponding to the (111), (200), (220), and (311) planes, respectively (JCPDS/PDF card no. 34-0394). These markers are included for reference and do not indicate that all corresponding reflections were experimentally resolved or definitively indexed. The CeO_2_ NP pattern exhibited broad, low intensity features consistent with limited long-range crystallographic ordering and a poorly crystalline or nanocrystalline character. The multi-REO material exhibited more numerous and comparatively better-resolved features, indicating greater apparent crystallographic ordering in the compositionally complex, multi-rare-earth-containing oxide-rich material. The available laboratory XRD patterns do not establish single-phase solid-solution formation or quantitative phase composition.

In contrast, the multi-REO NP pattern exhibited more numerous and comparatively better-resolved diffraction features, particularly in the low- and intermediate-angle regions, indicating greater apparent crystallographic ordering than the CeO_2_ NP pattern. The additional reflections were not consistent with cubic fluorite CeO_2_ alone and indicated contributions from other crystalline components. However, several reflections were broad, overlapping, or non-unique, and the candidate phases could not be distinguished reliably using the available laboratory XRD data. Features lacking a unique and defensible reference match were therefore retained as unassigned rather than being forced into a single-phase indexing scheme.

To document the basis for peak assignment, each observed reflection was classified as reference-matched, tentative, overlapping, or unassigned relative to the cubic fluorite CeO_2_ reference positions (PDF 34-0394; Table S6). For CeO_2_ NPs, no reflection could be defensibly matched to a fluorite reference line, with only weak, near-background features near the expected (111), (220), and (311) positions. For multi-REO NPs, several additional low-angle reflections (*e.g.*, 2*θ* ≈ 8.6, 13.3, 18.4, and 26.0°) did not correspond to fluorite CeO_2_ and were classified as tentative or unassigned, consistent with contributions from additional, non-single-phase rare-earth-containing components.

The available laboratory XRD data did not establish formation of a single-phase multi-rare-earth solid solution, homogeneous incorporation of all detected rare-earth elements into one crystallographic lattice, or quantitative phase composition. The multi-REO pattern was therefore interpreted as evidence of a structurally complex, multi-rare-earth-containing oxide-rich material.

XRD was therefore interpreted qualitatively in terms of diffraction behavior and apparent crystallographic ordering. No Scherrer coherent-domain-size estimates were retained, and no quantitative XRD-TEM size comparison was performed.

### 3.6. Morphological and compositional analysis

Following the crystallographic evaluation, SEM-EDS and TEM analyses were performed to examine the morphology, aggregation behavior, particle-size features, and elemental composition of the biosynthesized CeO_2_ NPs and multi-REO NPs. These analyses were used to determine whether the structural differences observed by XRD were accompanied by differences in particle shape, surface texture, and rare-earth elemental distribution.

#### 3.6.1. Scanning electron microscopy and energy-dispersive X-ray spectroscopy

SEM imaging revealed clear dried-state morphological differences between the two nanoparticle systems (Fig. 6). CeO_2_ NPs appeared predominantly spherical to quasi-spherical, with apparent particle dimensions of approximately 10–50 nm in the analyzed SEM regions. Individual particles were not fully isolated, and aggregation into larger clusters was evident. This observation is qualitatively consistent with the broad hydrodynamic-size distribution subsequently observed by DLS. However, direct numerical comparison between SEM and DLS is not appropriate because SEM represents dried-state morphology, whereas DLS measures the diffusion-related hydrodynamic dimensions of particles and aggregates in suspension.

**Fig. 6:**
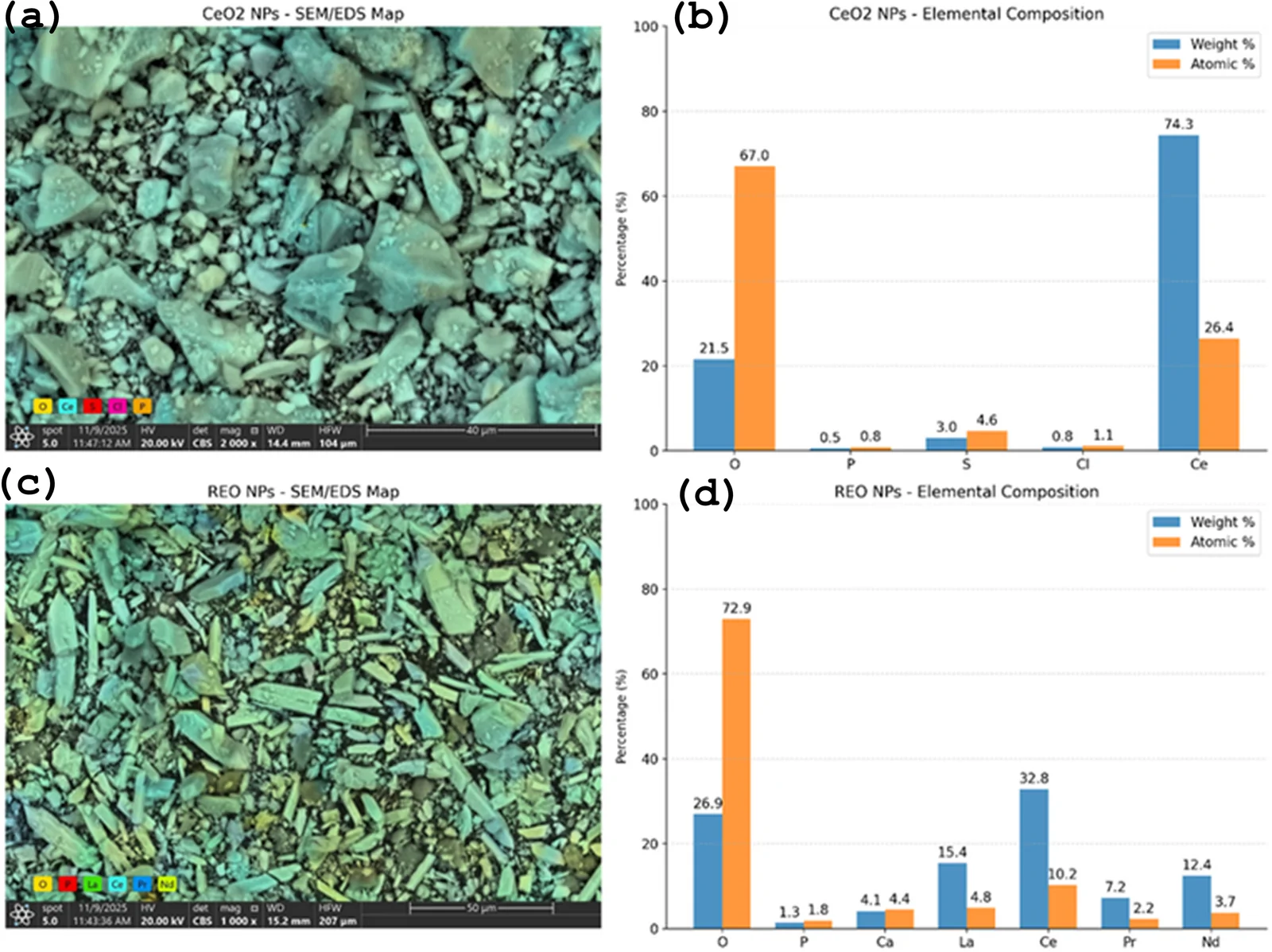
SEM morphology and regional EDS spectra of the biosynthesized CeO_2_-rich and multi-REO nanoparticle samples prepared using aqueous *M. oleifera* seed extract: the (a) SEM image of CeO_2_ NPs; (b) corresponding CeO_2_ EDS spectrum; (c) SEM image of multi-REO NPs; and (d) corresponding multi-REO EDS spectrum. The SEM images show the dried-state morphology and aggregation behavior of the powders, while the EDS spectra provide semi-quantitative local evidence of the Ce-rich and multi-rare-earth elemental compositions, respectively. EDS-derived percentages represent the analyzed regions and should not be interpreted as bulk stoichiometric compositions.

The CeO_2_ NP aggregates exhibited relatively smooth surfaces with limited visible faceting. This morphology may be associated with rapid nucleation and restricted domain growth under the low-temperature, extract-mediated synthesis conditions. The observation is qualitatively consistent with the broad, low-intensity XRD features, which indicate limited long-range crystallographic ordering and structural disorder. Nevertheless, SEM morphology alone cannot establish crystallinity, phase identity, or crystallographic-domain dimensions. Nevertheless, SEM morphology alone cannot establish crystallinity or phase identity.

In contrast, the multi-REO sample exhibited greater morphological heterogeneity, including irregular, faceted, approximately spherical, and short rod-like features, with apparent dried-state dimensions mainly in the range of approximately 20–100 nm. Dense agglomeration and compact network-like assemblies were more evident than in the CeO_2_ sample. The broader morphological distribution may be associated with differences in precursor chemistry, rare-earth composition, local hydrolysis and condensation behavior, phase complexity, and aggregation during drying. The faceted features and comparatively sharper XRD reflections are consistent with greater apparent structural development than in the CeO_2_-rich sample, although they do not independently establish the formation of a homogeneous mixed oxide or a single crystalline phase.

Regional EDS analysis provided semi-quantitative local information on the elemental composition of the two samples (Tables S2 and S3). For the CeO_2_ NPs, Ce and O were the dominant detected elements, with 26.4 at% Ce and 67.0 at% O in the analyzed region. Minor S, Cl, and P signals were also observed. These signals may originate from precursor-associated species, elements associated with extract-derived surface constituents, sample-preparation materials, or local support contributions.

The apparent Ce : O atomic ratio differed from the ideal stoichiometric ratio of bulk CeO_2_. However, EDS oxygen quantification is influenced by matrix effects, surface topography, interaction volume, adsorbed water, surface hydroxylation, and surface-associated organic constituents. The EDS-derived Ce : O ratio was therefore not used to infer the Ce^3+^/Ce^4+^ distribution, oxygen-vacancy concentration, or exact oxide stoichiometry. Such assignments would require oxidation-state- and bonding-sensitive methods such as XPS, XANES, Raman spectroscopy, and/or EELS.

For the multi-REO sample, the initial regional EDS analysis detected O (72.9 at%), Ce (10.2 at%), La (4.8 at%), Nd (3.7 at%), and Pr (2.2 at%), together with minor Ca and P signals. These results support the presence of multiple rare-earth elements in the analyzed region. However, because EDS probes a limited local interaction volume and is affected by particle morphology, aggregation, substrate contributions, and matrix corrections, the derived percentages should not be interpreted as the bulk stoichiometric composition of the sample. Accurate bulk elemental quantification would require ICP-OES or ICP-MS analysis.

SEM-EDS elemental mapping of the CeO_2_-rich sample showed spatially distributed Ce-L and O-K signals across the investigated micrometre-scale powder region (Fig. 7). When interpreted together with the XRD and regional EDS findings, the co-occurrence of Ce and O signals supports the presence of a cerium oxide-rich material throughout the mapped region (Table S4). Nevertheless, the maps do not determine the Ce^3+^/Ce^4+^ ratio, oxygen-vacancy concentration, chemical-bonding environment, and exact phase purity.

**Fig. 7:**
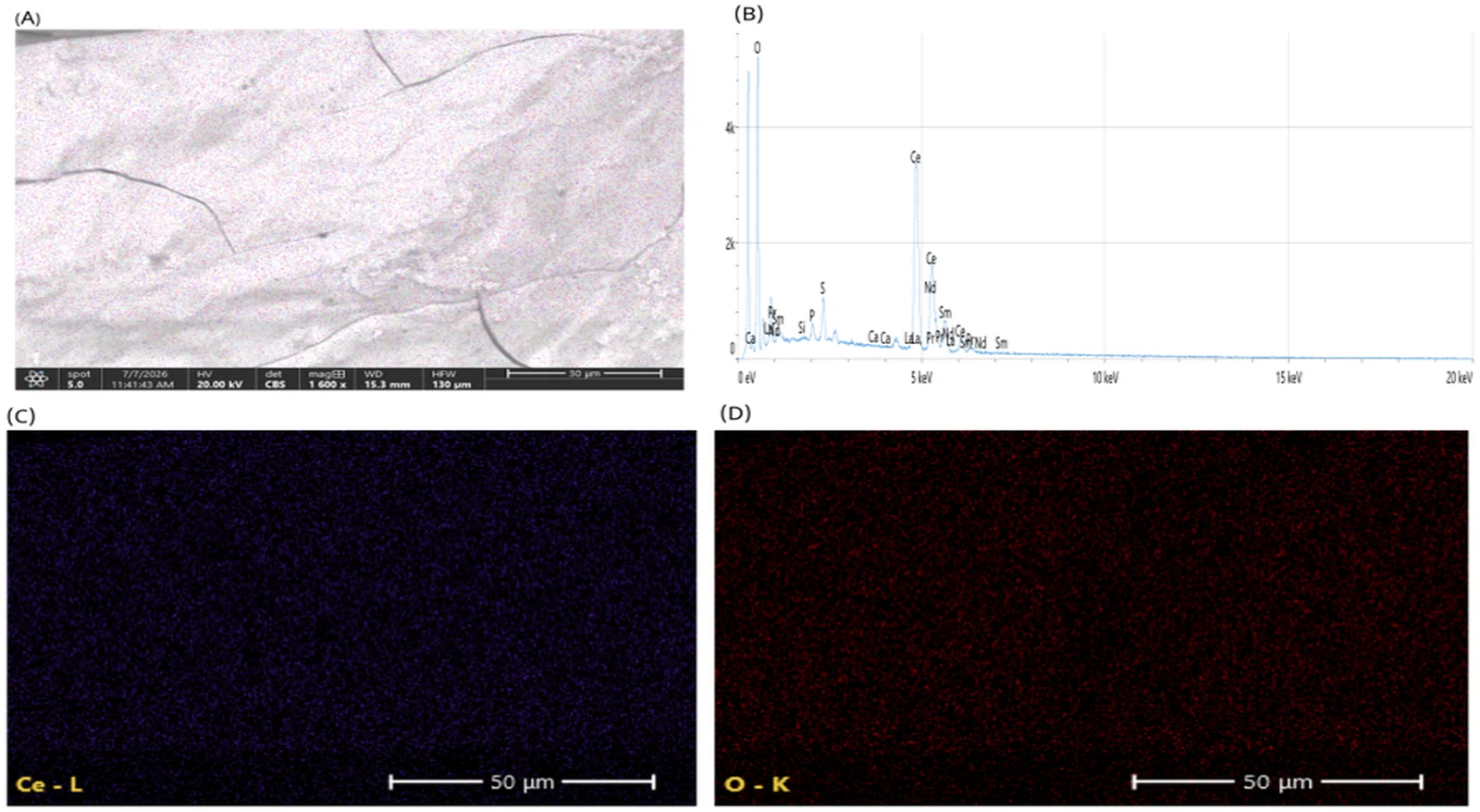
SEM-EDS characterization and elemental mapping of the CeO_2_-rich nanoparticle sample: the (A) SEM image of the mapped powder region; (B) corresponding EDS spectrum; (C) O-K elemental map; and (D) Ce-L elemental map. The spatial co-occurrence of Ce and O signals, when interpreted together with the XRD and regional EDS findings, supports the presence of a cerium oxide-rich material across the investigated micrometre-scale region. SEM-EDS mapping does not independently determine the Ce^3+^/Ce^4+^ ratio, oxygen-vacancy concentration, chemical-bonding environment, exact stoichiometry, or phase purity.

SEM-EDS elemental mapping of the multi-REO sample demonstrated the presence of O, Ce, La, Nd, Pr, and a minor Sm component across the investigated powder region (Fig. 8). The mapped region contained 65.1 at% O, 14.2 at% Ce, 6.9 at% La, 6.0 at% Nd, 2.0 at% Pr, and 0.9 at% Sm. When normalized to the total detected rare-earth atomic fraction, Ce represented approximately 47.3%, followed by La (23.0%), Nd (20.0%), Pr (6.7%), and Sm (3.0%). The analyzed region was therefore Ce-rich relative to the total detected rare-earth atomic fraction (Table S5).

**Fig. 8:**
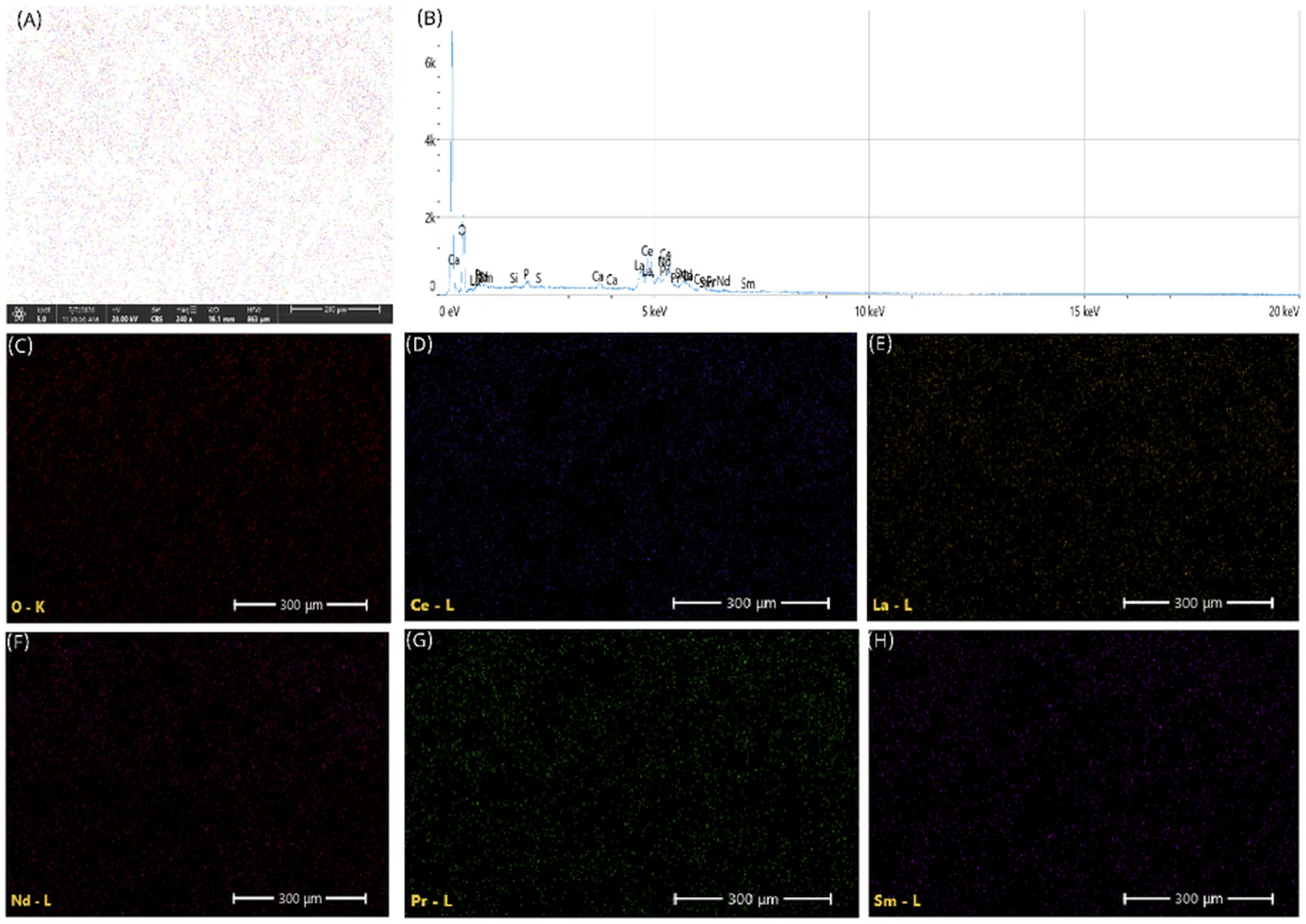
SEM-EDS characterization and elemental mapping of the multi-REO sample: the (A) SEM image of the mapped powder region; (B) corresponding EDS spectrum; and elemental maps of (C) O-K, (D) Ce-L, (E) La-L, (F) Nd-L, (G) Pr-L, and (H) Sm-L. The EDS spectrum and elemental maps demonstrate the presence and micrometre-scale regional distribution of O, Ce, La, Nd, and Pr, with Sm detected as a minor rare-earth constituent. These findings support the operational description of the product as a Ce-rich, multi-rare-earth-containing oxide-rich material; however, SEM-EDS mapping does not independently establish atomic-scale elemental mixing, specific oxidation states, or single-phase solid-solution formation.

A regional EDS analysis associated with the same Site 87 dataset yielded 65.9 at% O, 14.8 at% Ce, 6.2 at% La, 5.6 at% Nd, 2.6 at% Pr, and 0.9 at% Sm (Table S5). The broadly comparable proportions support local consistency of the detected elemental profile within the investigated site. However, the mapping and regional analyses were derived from the same site or spatially related areas and should not be interpreted as independent experimental replicates or as evidence of bulk compositional homogeneity.

The elemental maps demonstrate micrometre-scale regional co-occurrence of multiple rare-earth signals but do not establish atomic-scale mixing, homogeneous incorporation of all detected elements within individual nanoparticles, or occupation of a common crystallographic lattice. Furthermore, SEM-EDS cannot distinguish conclusively among a true mixed-oxide solid solution, a multiphase rare-earth oxide assemblage, physically associated rare-earth-containing domains, an oxide/oxyhydroxide-rich composite, or a poorly crystalline/amorphous rare-earth-containing precipitate. The product is therefore described operationally as a Ce-rich, multi-rare-earth-containing oxide-rich nanoparticulate material rather than as a confirmed single-phase solid solution.

Minor P, S, Ca, and Si signals may originate from precursor-associated impurities, elements associated with extract-derived surface constituents, residual processing species, or sample-support contributions. Their chemical forms, bonding environments, and precise origins cannot be resolved using EDS alone. Definitive bulk-composition, phase, oxidation-state, and nanoscale-distribution assignments would require complementary ICP-OES or ICP-MS, XPS, Raman spectroscopy, high-resolution STEM-EDS, and higher-resolution diffraction with full-pattern refinement using defensible candidate structural models.

#### 3.6.2. Transmission electron microscopy (TEM)

TEM analysis provided further insight into the nanoscale morphology and aggregation behavior of the synthesized nanoparticles (Fig. 9). CeO_2_ NPs appeared mainly as spherical to quasi-spherical particles with core diameters predominantly in the range of approximately 5–15 nm. This size range is consistent with the nanoscale population observed in the DLS analysis, although DLS also detected larger hydrodynamic aggregates in aqueous suspensions. The difference between TEM and DLS is expected because TEM images dried particles, whereas DLS includes hydration layers, surface-bound organic residues, and interparticle aggregates.

**Fig. 9:**
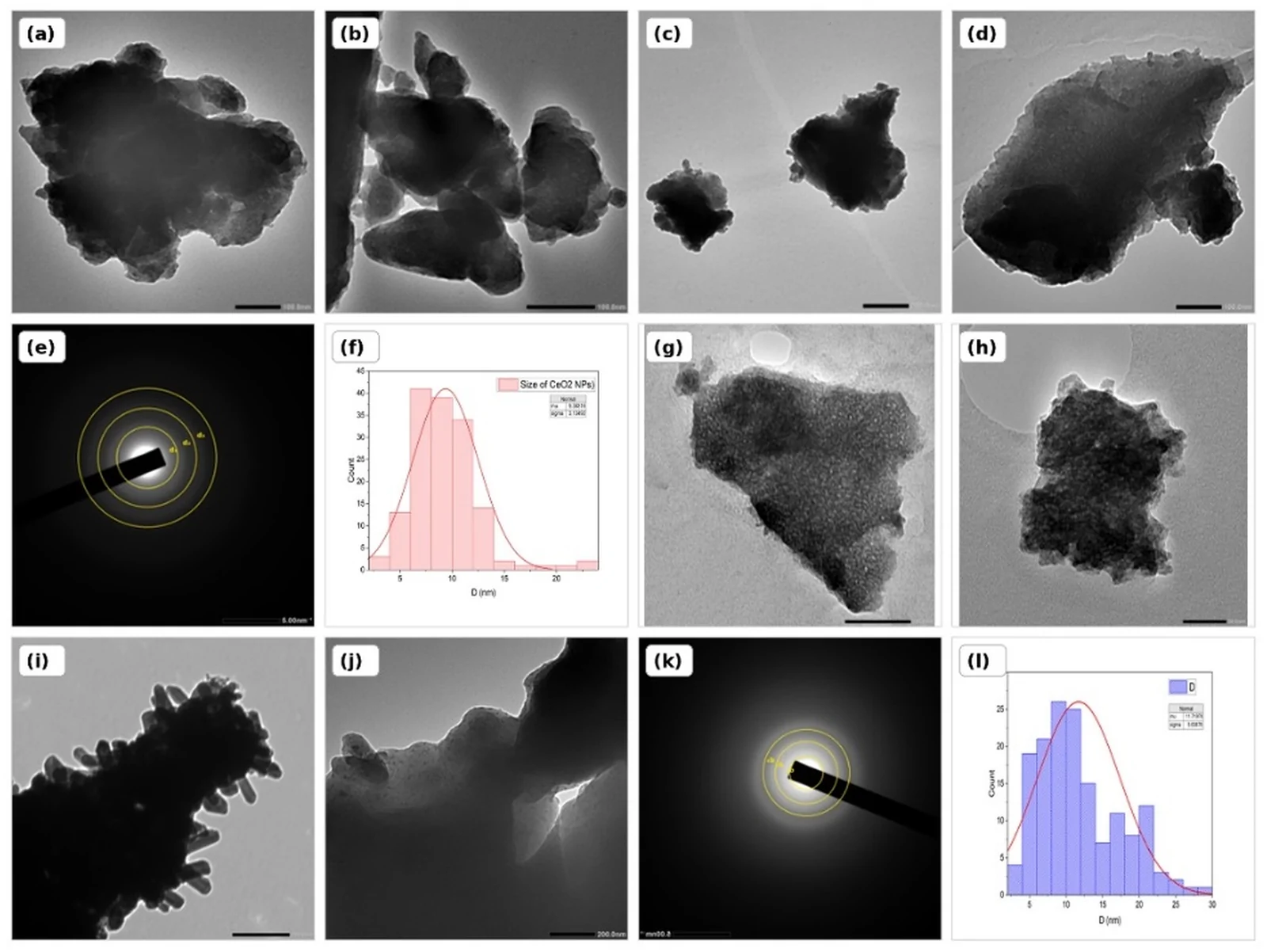
TEM and SAED characterization of biosynthesized CeO_2_ NPs and multi-REO NPs. (a–f) CeO_2_ NPs showing predominantly quasi-spherical particles, aggregation, contrast variations, diffuse ring-like SAED features, and projected particle dimensions mainly within approximately 5–15 nm. (g–l) Multi-REO NPs showing greater morphological heterogeneity, including approximately spherical, irregular, faceted, and short rod-like particles, together with aggregation, diffuse or mixed SAED features, and projected particle dimensions mainly within approximately 20–60 nm. TEM contrast may be influenced by particle thickness, mass–thickness, aggregation, local composition, orientation, and diffraction conditions and was not interpreted quantitatively as evidence of specific elemental distributions or phase identities. The SAED patterns were not indexed sufficiently for definitive phase identification. The observed morphological and diffraction differences are consistent with differences in precursor chemistry and compositional complexity but do not establish a single-phase mixed oxide, homogeneous incorporation of the detected rare-earth elements, or a confirmed core–shell architecture.

High-magnification TEM images of CeO_2_ NPs showed contrast variations between darker and lighter regions. These variations may reflect differences in particle thickness, mass–thickness, aggregation, inorganic mass density, local composition, orientation, diffraction conditions, or surface-associated organic material. The contrast variations were therefore not interpreted quantitatively as evidence of specific elemental distributions, phase identities, or a confirmed core–shell architecture. Confirmation of a discrete surface shell would require spatially resolved compositional analysis, shell-thickness measurements, or complementary STEM-EDS or EELS characterization. The SAED patterns exhibited diffuse ring-like features, consistent with limited long-range ordering and contributions from nanoscale crystalline and/or structurally disordered regions. Because the rings were not indexed reliably, the SAED patterns were not used for definitive phase identification.

The multi-REO NPs exhibited larger projected particle dimensions and greater morphological diversity than the CeO_2_-rich sample, with visible particles mainly distributed within approximately 20–60 nm. The material contained approximately spherical particles together with irregular, faceted, and short rod-like features, indicating heterogeneous particle-growth behavior. Differences in TEM contrast between and within the samples may reflect variations in particle thickness, mass–thickness, aggregation, local composition, orientation, and diffraction conditions. The contrast differences were therefore not interpreted quantitatively as evidence of specific elemental distributions or phase identities. The faceted and short rod-like features demonstrate morphological heterogeneity but do not establish their crystallographic origin without indexed SAED patterns, lattice-fringe analysis, or high-resolution STEM characterization.

Compared with CeO_2_ NPs, the multi-REO sample showed stronger aggregation, forming dense interconnected assemblies. This aggregation may arise from differences in surface chemistry, particle shape, and interparticle interactions. The coexistence of several rare-earth cations may also generate a more heterogeneous surface-energy landscape, favoring anisotropic or directionally varied growth. Nevertheless, the exact crystallographic origin of the rod-like and faceted morphologies cannot be established from TEM images alone and would require indexed SAED patterns or high-resolution lattice-fringe analysis.

Overall, SEM-EDS and TEM analyses showed that the CeO_2_-rich sample contained smaller, predominantly quasi-spherical particles, whereas the multi-REO material exhibited larger projected dimensions, greater morphological heterogeneity, and stronger dried-state aggregation. These findings were qualitatively consistent with the differences in diffraction behavior and surface-associated FTIR features. However, the microscopy results did not independently establish phase identity, crystallographic-domain size, atomic-scale elemental mixing, homogeneous rare-earth incorporation, or causal relationships between morphology and the measured cell-free assay responses.

The XRD and TEM results provided complementary but distinct structural information. XRD was used qualitatively to assess diffraction behavior and apparent long-range crystallographic ordering, whereas TEM provided two-dimensional projected measurements of visible particle dimensions and morphology. Because the available XRD features were broad, overlapping, weak, or insufficiently resolved for defensible peak-width determination and phase-specific assignment, no Scherrer coherent-diffraction-domain-size estimates or quantitative XRD–TEM size comparisons were reported for either nanoparticle system.

### 3.7. Hydrodynamic size distribution and aggregation behavior

Dynamic light scattering (DLS) analysis was used to evaluate the dispersion-state behavior of CeO_2_ NPs and multi-REO NPs in aqueous suspensions (Fig. 10). Unlike TEM and SEM, which examine dried particles, DLS measures the apparent hydrodynamic diameter of dispersed species, including the inorganic core, adsorbed surface residues, hydration layer, and any interparticle aggregates. Therefore, the DLS results were interpreted as hydrodynamic dispersion data rather than as a direct measurement of primary particle size.

**Fig. 10:**
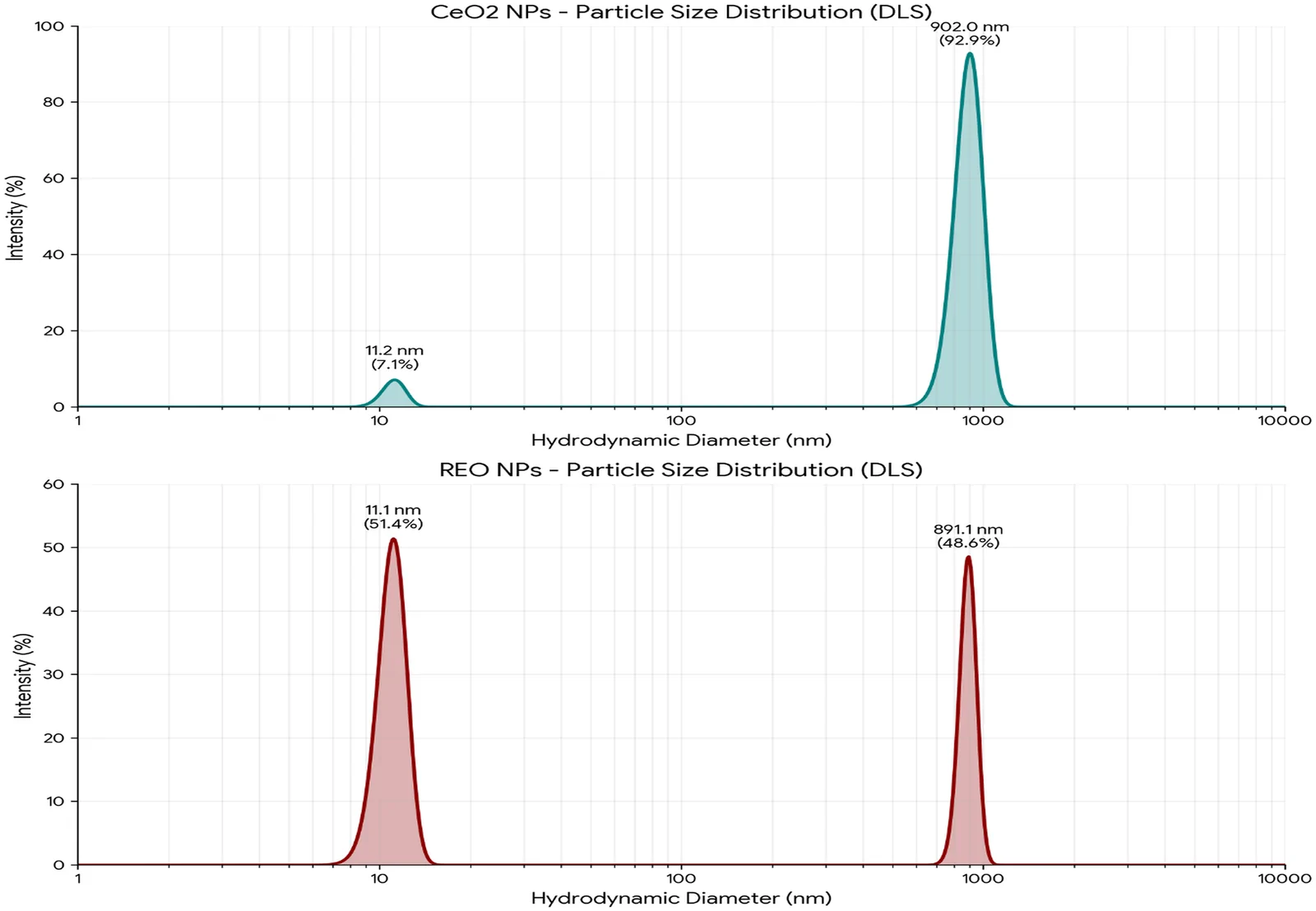
Dynamic light scattering (DLS) size distribution profiles of biosynthesized CeO_2_ NPs and multi-REO NPs in aqueous suspensions. CeO_2_ NPs showed a bimodal hydrodynamic size distribution with a nanoscale population centered at approximately 10.7–11.2 nm and a secondary aggregate population at approximately 902–914 nm. Multi-REO NPs exhibited a similar bimodal distribution with a nanoscale population at approximately 10.7–11.1 nm and an aggregate population at approximately 891–895 nm. Number- and volume-weighted distributions indicated the presence of a substantial nanoscale fraction, whereas intensity-weighted distributions were strongly influenced by larger aggregates. Both dispersions remained highly polydisperse and aggregated.

Both nanoparticle systems exhibited bimodal hydrodynamic size distributions, indicating the coexistence of a nanoscale particle fraction and a secondary population of larger aggregates. For CeO_2_ NPs, NICOMP multimodal analysis resolved a nanoscale population centered at approximately 10.7–11.2 nm and a larger aggregate population at approximately 902–914 nm. The intensity-, volume-, and number-weighted mean diameters were 276.1 ± 578.0 nm, 66.8 ± 139.8 nm, and 31.1 ± 65.2 nm, respectively. The high reported PDI value of 4.381 indicates a strongly heterogeneous and highly polydisperse aqueous dispersion.

Similarly, multi-REO NPs showed a bimodal hydrodynamic profile, with a nanoscale population at approximately 10.7–11.1 nm and an aggregate population at approximately 891–895 nm. The corresponding intensity-, volume-, and number-weighted mean diameters were 244.6 ± 445.9 nm, 50.9 ± 92.7 nm, and 27.7 ± 50.5 nm, respectively, with a reported PDI of 3.323. Although this PDI is lower than that of CeO_2_ NPs, both values indicate highly polydisperse and aggregated systems rather than monodisperse colloidal suspensions.

The intensity-weighted distributions should be interpreted with particular caution because scattered light intensity scales strongly with particle diameter, approximately following *I* ∝ *d*^6^ under Rayleigh-type scattering conditions. Consequently, even a small fraction of large aggregates can dominate the intensity signal and shift the apparent mean diameter toward larger values. In contrast, the number- and volume-weighted distributions suggest the presence of a substantial nanoscale fraction in both samples. For these highly polydisperse systems, the resolved modal populations provide more meaningful information than broad average diameters alone.

The DLS-derived hydrodynamic dimensions were substantially larger than the TEM-observed core dimensions, consistent with hydration, surface-associated organic layers, and aggregation in aqueous suspensions. No comparison with an XRD-derived size was made because Scherrer estimates were not retained.

Zeta-potential analysis (Table 4) showed that both nanoparticle systems exhibited negative electrokinetic potentials under the applied aqueous measurement conditions. CeO_2_ NPs exhibited a more negative zeta potential (−25.5 mV) than multi-REO NPs (−5.09 mV), indicating stronger electrostatic repulsion and comparatively greater electrostatic stabilization of the CeO_2_ suspension. In contrast, the low zeta-potential magnitude of the multi-REO suspension indicates weak electrostatic stabilization and a greater tendency toward aggregation. This interpretation is consistent with the broad hydrodynamic-size distribution observed by DLS. However, zeta potential reflects electrostatic contributions under the specific measurement conditions and does not independently establish long-term colloidal stability. Possible steric contributions from phytochemical- or protein-derived surface association and partial steric stabilization also cannot be evaluated from zeta-potential measurements alone. The observed difference may therefore arise from variations in surface chemistry, surface-associated organic groups, aggregation state, and residual ionic species in the respective dispersions.

**Table 4:** Zeta-potential characteristics of CeO_2_ and multi-REO nanoparticle suspensions in water^a^

Sample	Instrument-reported zeta potential (mV)	Instrument-reported zeta-distribution deviation (mV)	Conductivity (mS cm^−1^)	Temperature (°C)
CeO_2_ NPs	−25.5	7.22	0.0432	25.0
Multi-REO NPs	−5.09	4.48	0.0629	24.9

a The zeta-deviation values represent the distribution deviations reported by the instrument and should not be interpreted as standard deviations from independent nanoparticle preparations.

Comparison between DLS- and TEM-derived dimensions should be made cautiously because the two techniques characterize different aspects of the nanoparticle systems. TEM provides two-dimensional projected measurements of visible particle dimensions under dry, high-vacuum imaging conditions, whereas DLS measures the hydrodynamic dimensions of dispersed species in aqueous suspensions, including contributions from surface-associated layers, hydration, and aggregation. For CeO_2_ NPs, the smaller DLS population was broadly consistent with the nanoscale dimensions observed by TEM, whereas the larger hydrodynamic population indicated the presence of aggregates in aqueous suspensions. For multi-REO NPs, differences between the DLS and TEM dimensions were attributed to the multimodal size distribution, heterogeneous and non-spherical particle morphology, surface-associated layers, hydration, and aggregation. No quantitative comparison with an XRD-derived size was made because Scherrer coherent-domain-size estimates were not retained.

Overall, DLS analysis indicated that both CeO_2_ NPs and multi-REO NPs contained nanoscale particle populations but also exhibited substantial aggregation in aqueous suspensions. The lower PDI and smaller number- and volume-weighted mean diameters of multi-REO NPs suggest comparatively lower dispersion heterogeneity than CeO_2_ NPs under the applied measurement conditions. However, the zeta-potential values indicated distinct electrostatic stabilization profiles: CeO_2_ NPs exhibited a moderately negative zeta potential of −25.5 mV, consistent with comparatively stronger electrostatic repulsion, whereas multi-REO NPs exhibited a value of −5.09 mV, indicating weak electrostatic stabilization and a greater tendency toward aggregation. Thus, the smaller hydrodynamic size metrics and lower PDI of multi-REO NPs should not be interpreted as evidence of superior colloidal stability. Collectively, the DLS and zeta-potential findings indicate broad, aggregation-prone dispersions in both systems, with CeO_2_ NPs showing comparatively greater electrostatic stabilization under the applied aqueous measurement conditions. Because zeta potential and hydrodynamic size are sensitive to the pH, ionic strength, particle concentration, dispersion protocol, and measurement medium, these findings describe the suspensions only under the specific conditions employed and do not establish long-term stability or behavior in physiological media.

### 3.8. Integrated composition–structure–property interpretation

Taken together, the results indicate that the CeO_2_-rich and multi-REO nanoparticle systems differ not only in rare-earth composition but also in apparent crystallographic ordering, morphology, surface functionality, aggregation behavior, zeta potential, and optical-edge characteristics. The CeO_2_-rich sample exhibited broad, low-intensity XRD features, a predominantly quasi-spherical morphology, and a more negative zeta potential than the multi-REO sample. In contrast, the multi-REO material showed comparatively sharper and more complex XRD reflections, greater morphological heterogeneity, a lower-magnitude zeta potential, and broader apparent Urbach-edge tailing. These findings demonstrate that the harmonized synthesis procedures produced structurally, optically, and colloidally distinct materials.

Ce^3+^/Ce^4+^-related surface redox behavior and oxygen-defect chemistry are frequently associated with the properties of CeO_2_-based nanomaterials. However, the Ce^3+^/Ce^4+^ distribution and oxygen-vacancy concentration were not directly quantified in the present samples. These features are therefore considered plausible, literature-supported contributors rather than experimentally established characteristics. Similarly, the coexistence of Ce, La, Nd, and Pr, with Sm detected locally at a low level, in the multi-REO material may generate compositionally complex local coordination environments that influence particle growth, surface hydroxylation, aggregation, and optical-edge behavior. Nevertheless, the available data do not establish whether these effects arise from a common mixed lattice, multiple oxide or oxyhydroxide-rich phases, physically associated domains, or a poorly crystalline rare-earth-containing precipitate.

The HPLC-DAD and FTIR findings further suggest that extract-derived constituents may contribute to rare-earth-ion coordination, particle-growth regulation, surface functionalization, aggregation, and interactions with reactive species or proteins. However, the identities and abundances of specific surface-bound molecules were not directly determined. The generally higher responses of the multi-REO sample in several preliminary cell-free assays raise the possibility of collective composition-associated effects, but true rare-earth synergy was not demonstrated. Precursor chemistry, phase complexity, particle-size distribution, aggregation, surface charge, and extract-derived surface-associated constituents may also contribute. The proposed composition–structure–property–screening-response relationship should therefore be interpreted as an evidence-supported associative framework rather than a definitive causal mechanism.

The possible implications of these physicochemical differences for preliminary cell-free screening responses are considered in the following sections.

### 3.9. Preliminary cell-free biochemical screening

The biosynthesized CeO_2_ NPs and multi-REO NPs were evaluated over 50–200 µg mL^−1^ using preliminary, assay-specific, cell-free models of radical/reactive-species scavenging, total antioxidant capacity, reducing power, AChE response, BSA thermal-denaturation inhibition, and α-amylase and α-glucosidase inhibition. These assays provide comparative biochemical screening profiles and should not be interpreted as direct evidence of antioxidant, antidiabetic, anti-inflammatory, neuroprotective, or therapeutic efficacy.

#### 3.9.1. Radical- and reactive-species-scavenging responses

Oxidative stress is associated with the excessive formation of reactive oxygen and nitrogen species, including hydroxyl radicals (HO˙), nitric oxide (NO˙), and hydrogen peroxide (H_2_O_2_). To compare the cell-free redox-related responses of the two nanoparticle systems, a panel of complementary scavenging assays was performed, including DPPH˙, ABTS˙^+^, NO˙, HO˙, and H_2_O_2_ assays (Fig. 11A–E). DPPH˙ and ABTS˙^+^ were used as stable radical models, whereas NO˙, HO˙, and H_2_O_2_ provided additional reactive-species screening models.

**Fig. 11:**
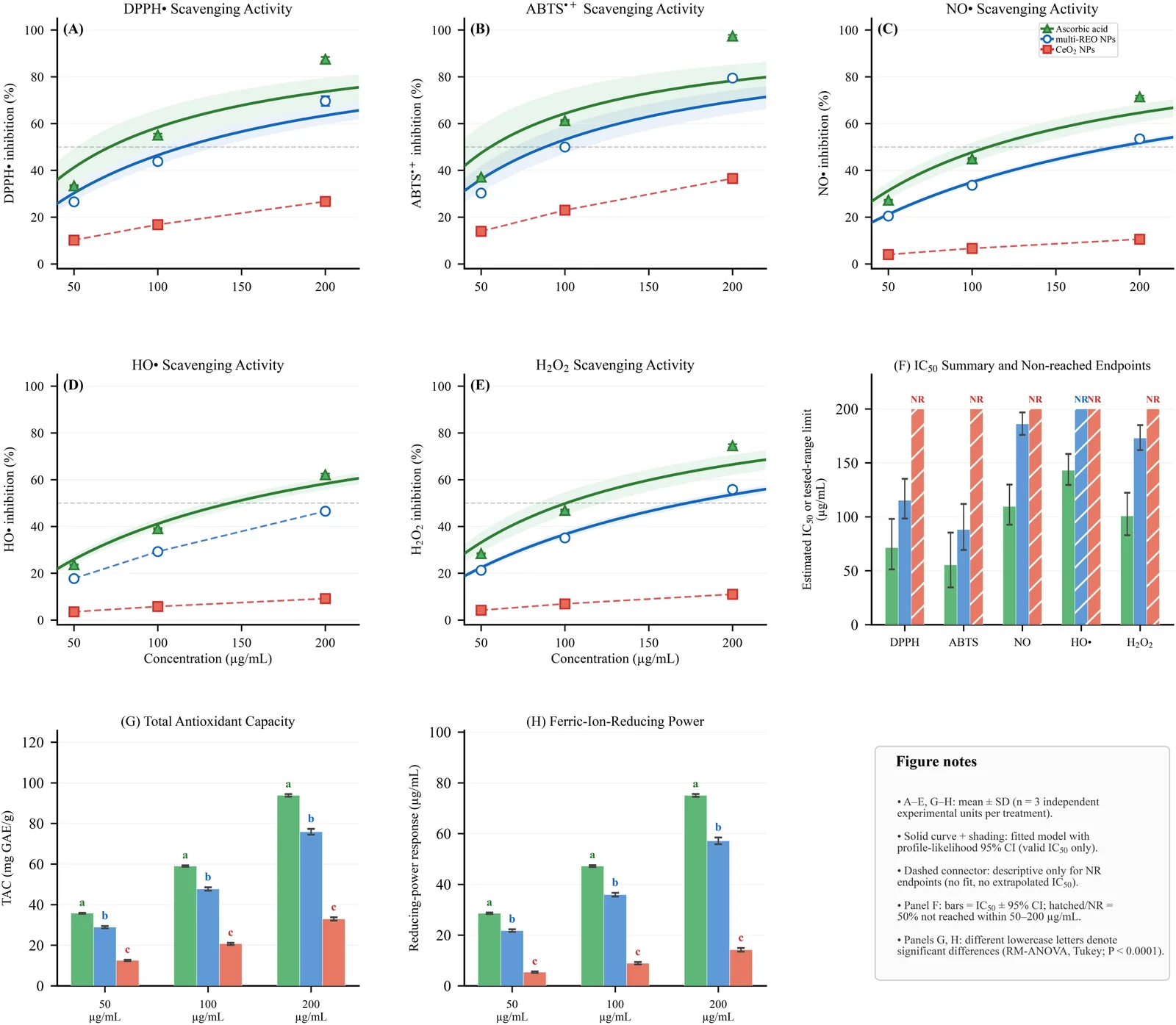
Preliminary cell-free redox-related responses of biosynthesized CeO_2_ NPs and multi-REO NPs compared with ascorbic acid as the reference compound. (A–E) Concentration-dependent responses in the DPPH˙, ABTS˙^+^, NO˙, HO˙, and H_2_O_2_ scavenging assays over 50–200 µg mL^−1^. Data are presented as mean ± SD across three independently prepared synthesis batches for each nanoparticle system and three independent assay runs for ascorbic acid (*n* = 3 independent experimental units per treatment). The horizontal dashed gray line denotes 50% inhibition. For endpoints that reached or exceeded 50% inhibition within the tested range, solid lines represent constrained concentration–response fits, and the shaded regions illustrate model-based uncertainty derived from the profile-likelihood 95% confidence limits of the corresponding IC_50_ estimates. For endpoints that did not reach 50% inhibition within the tested range (NR), dashed lines connect the observed means for descriptive purposes only and do not represent fitted concentration–response models or extrapolated IC_50_ estimates. Error bars smaller than the plotted symbols may not be visible. (F) Summary of estimable IC_50_ values and endpoints at which 50% inhibition was not reached. Error bars represent profile-likelihood 95% confidence intervals for the estimable IC_50_ values. Hatched bars marked NR and terminating at 200 µg mL^−1^ indicate the upper tested concentration and do not represent estimated IC_50_ values of 200 µg mL^−1^. (G) Total antioxidant capacity, expressed as milligrams of gallic acid equivalents per gram of sample (mg GAE g^−1^). (H) Ferric-ion-reducing power. For panels (A–E), (G), and (H), the effects of treatment, concentration, and the treatment × concentration interaction were evaluated using two-way mixed-design repeated-measures ANOVA, with treatment analyzed as the between-unit factor and concentration as the within-unit repeated factor. Sphericity was not assumed, and the Geisser–Greenhouse correction was applied to the concentration and interaction effects. Tukey-adjusted multiple comparisons were performed among treatments at each concentration. Different lowercase letters indicate significant differences among treatments within the same concentration (*P* < 0.05). Because only three concentrations were evaluated, the fitted curves, IC_50_ estimates, and associated confidence intervals should be interpreted cautiously. NR, 50% response not reached within the tested concentration range.

Across the tested concentration range, both CeO_2_ NPs and multi-REO NPs generally exhibited concentration-dependent increases in scavenging response. Multi-REO NPs consistently produced greater apparent inhibition than CeO_2_ NPs at equivalent concentrations. For endpoints at which 50% response was not reached within the tested concentration range of 50–200 µg mL^−1^, the endpoint was designated as not reached within the tested range (NR), and no extrapolated numerical IC_50_ estimate or confidence interval was reported. Such endpoints were excluded from IC_50_ ratio-based comparisons.

The measured scavenging responses may reflect combined contributions from nanoparticle surface chemistry, aggregation, surface-associated extract constituents, and interactions at hydroxylated or defect-related surface sites. However, the respective contributions of the inorganic components and residual or surface-associated phytochemicals could not be separated quantitatively because an extract-only control was not included.

The observed scavenging responses may arise from the combined contribution of nanoparticle surface chemistry and residual phytochemical capping agents. Comparable behavior has been reported for green-synthesized ZnO nanoparticles produced using pomegranate husk extract, where strong DPPH and ABTS radical-scavenging activity was attributed to combined contributions of the metal-oxide surface and plant-derived constituents.^16^

##### 3.9.1.1. DPPH˙ scavenging (Fig. 11A)

In the DPPH˙ assay, all treatments exhibited concentration-dependent increases in radical-scavenging response, with multi-REO NPs consistently producing greater inhibition than CeO_2_ NPs at equivalent concentrations. At 200 µg mL^−1^, multi-REO NPs achieved 69.63 ± 2.10% inhibition, compared with 26.68 ± 1.29% for CeO_2_ NPs and 87.55 ± 0.96% for ascorbic acid. Data are presented as mean ± SD across three independent experimental units (*n* = 3).

Constrained nonlinear regression, with the lower and upper response plateaus fixed at 0% and 100%, respectively, and the Hill slope fixed at 1, yielded an estimated IC_50_ of 115.3 µg mL^−1^ for multi-REO NPs (95% CI: 98.4–135.3 µg mL^−1^; *R*^2^ = 0.933) and 71.4 µg mL^−1^ for ascorbic acid (95% CI: 51.3–98.1 µg mL^−1^; *R*^2^ = 0.822). CeO_2_ NPs did not reach 50% inhibition within the tested concentration range of 50–200 µg mL^−1^; therefore, the corresponding IC_50_ endpoint was designated as not reached within the tested range (NR), and no extrapolated numerical IC_50_ estimate or confidence interval was reported.

Two-way mixed-design repeated-measures ANOVA, with concentration treated as the within-unit repeated factor and using the Geisser–Greenhouse correction, revealed significant effects of treatment [*F*(2,6) = 1261.97, *P* < 0.0001] and concentration [*F*(1.000,6.000) = 14 571.78, *P* < 0.0001], together with a significant treatment × concentration interaction [*F*(2.000,6.000) = 1265.42, *P* < 0.0001]. The significant interaction indicated that the magnitude of the concentration-dependent increase differed among treatments. Tukey-adjusted multiple comparisons identified significant differences among treatments at each tested concentration (*P* < 0.05), as indicated by different lowercase letters in Fig. 11A.

Overall, multi-REO NPs exhibited a stronger apparent DPPH˙-scavenging response than CeO_2_ NPs under the applied cell-free assay conditions but remained less active than ascorbic acid. The estimated reference-to-multi-REO IC_50_ ratio was 0.62, indicating that multi-REO NPs required a higher concentration than ascorbic acid to achieve 50% DPPH˙ scavenging. This ratio is an assay-specific comparative descriptor and should not be interpreted as evidence of therapeutic potency.

##### 3.9.1.2. ABTS˙^+^ scavenging (Fig. 11B)

In the ABTS˙^+^ radical-cation decolorization assay, all treatments exhibited concentration-dependent increases in scavenging response, with multi-REO NPs consistently producing greater inhibition than CeO_2_ NPs at equivalent concentrations. At 200 µg mL^−1^, multi-REO NPs achieved 79.47 ± 1.21% inhibition, compared with 36.52 ± 0.74% for CeO_2_ NPs and 97.39 ± 0.55% for ascorbic acid. Data are presented as mean ± SD across three independent experimental units (*n* = 3).

Constrained nonlinear regression, with the lower and upper response plateaus fixed at 0% and 100%, respectively, and the Hill slope fixed at 1, yielded an estimated IC_50_ of 88.2 µg mL^−1^ for multi-REO NPs (95% CI: 69.3–112.0 µg mL^−1^; *R*^2^ = 0.880) and 55.5 µg mL^−1^ for ascorbic acid (95% CI: 34.6–85.4 µg mL^−1^; *R*^2^ = 0.739). CeO_2_ NPs did not reach 50% inhibition within the tested concentration range of 50–200 µg mL^−1^; therefore, the corresponding IC_50_ endpoint was designated as not reached within the tested range (NR), and no extrapolated numerical IC_50_ estimate or confidence interval was reported.

Two-way mixed-design repeated-measures ANOVA, with concentration treated as the within-unit repeated factor and using the Geisser–Greenhouse correction, revealed significant effects of treatment [*F*(2,6) = 3694.18, *P* < 0.0001]and concentration [*F*(1.002,6.011) = 63 452.30, *P* < 0.0001], together with a significant treatment × concentration interaction [*F*(2.004,6.011) = 4091.13, *P* < 0.0001]. The significant interaction indicated that the magnitude of the concentration-dependent increase differed among treatments. Tukey-adjusted multiple comparisons identified significant differences among treatments at each tested concentration (*P* < 0.05), as indicated by different lowercase letters in Fig. 11B.

Overall, multi-REO NPs exhibited a stronger apparent ABTS˙^+^-scavenging response than CeO_2_ NPs under the applied cell-free assay conditions but remained less active than ascorbic acid. The estimated reference-to-multi-REO IC_50_ ratio was 0.63, indicating that multi-REO NPs required a higher concentration than ascorbic acid to achieve 50% ABTS˙^+^ scavenging. This ratio is an assay-specific comparative descriptor and should not be interpreted as evidence of therapeutic potency.

##### 3.9.1.3. NO˙ scavenging (Fig. 11C)

In the nitric oxide radical-scavenging assay, all treatments exhibited concentration-dependent increases in scavenging response, with multi-REO NPs consistently producing greater inhibition than CeO_2_ NPs at equivalent concentrations. At 200 µg mL^−1^, multi-REO NPs achieved 53.49 ± 1.21% inhibition, compared with 10.55 ± 0.74% for CeO_2_ NPs and 71.42 ± 0.55% for ascorbic acid. Data are presented as mean ± SD across three independent experimental units (*n* = 3).

Constrained nonlinear regression, with the lower and upper response plateaus fixed at 0% and 100%, respectively, and the Hill slope fixed at 1, yielded an estimated IC_50_ of 186.1 µg mL^−1^ for multi-REO NPs (95% CI: 176.0–196.8 µg mL^−1^; *R*^2^ = 0.988) and 109.7 µg mL^−1^ for ascorbic acid (95% CI: 92.8–129.9 µg mL^−1^; *R*^2^ = 0.928). CeO_2_ NPs did not reach 50% inhibition within the tested concentration range of 50–200 µg mL^−1^; therefore, the corresponding IC_50_ endpoint was designated as not reached within the tested range (NR), and no extrapolated numerical IC_50_ estimate or confidence interval was reported.

Two-way mixed-design repeated-measures ANOVA, with concentration treated as the within-unit repeated factor and using the Geisser–Greenhouse correction, revealed significant effects of treatment [*F*(2,6) = 3511.33, *P* < 0.0001] and concentration [*F*(1.018,6.107) = 29 579.83, *P* < 0.0001], together with a significant treatment × concentration interaction [*F*(2.036,6.107) = 4734.22, *P* < 0.0001]. The significant interaction indicated that the magnitude of the concentration-dependent increase differed among treatments. Tukey-adjusted multiple comparisons identified significant differences among treatments at each tested concentration (*P* < 0.05), as indicated by different lowercase letters in Fig. 11C.

The estimated IC_50_ of multi-REO NPs was close to the upper limit of the tested concentration range and should therefore be interpreted cautiously. Overall, multi-REO NPs exhibited a stronger apparent NO˙-scavenging response than CeO_2_ NPs under the applied cell-free assay conditions but remained less active than ascorbic acid. The estimated reference-to-multi-REO IC_50_ ratio was 0.59, indicating that multi-REO NPs required a higher concentration than ascorbic acid to achieve 50% NO˙ scavenging. No IC_50_-based ratio was calculated for CeO_2_ NPs because 50% inhibition was not reached within the tested range. These comparisons are assay-specific descriptors and should not be interpreted as evidence of therapeutic potency.

##### 3.9.1.4. HO˙ scavenging (Fig. 11D)

In the hydroxyl-radical scavenging assay, all treatments exhibited concentration-dependent increases in scavenging response, with multi-REO NPs consistently producing greater inhibition than CeO_2_ NPs at equivalent concentrations. At 200 µg mL^−1^, multi-REO NPs achieved 46.52 ± 1.02% inhibition, compared with 9.17 ± 0.68% for CeO_2_ NPs and 62.10 ± 0.52% for ascorbic acid. Data are presented as mean ± SD across three independent experimental units (*n* = 3).

Ascorbic acid was the only treatment that reached 50% inhibition within the tested concentration range. Constrained nonlinear regression, with the lower and upper response plateaus fixed at 0% and 100%, respectively, and the Hill slope fixed at 1, yielded an estimated IC_50_ of 143.1 µg mL^−1^ for ascorbic acid (95% CI: 129.6–158.3 µg mL^−1^; *R*^2^ = 0.968). Neither multi-REO NPs nor CeO_2_ NPs reached 50% inhibition within the tested range of 50–200 µg mL^−1^. Their corresponding IC_50_ endpoints were therefore designated as not reached within the tested range (NR), and no extrapolated numerical IC_50_ estimates or confidence intervals were reported.

Two-way mixed-design repeated-measures ANOVA, with concentration treated as the within-unit repeated factor and using the Geisser–Greenhouse correction, revealed significant effects of treatment [*F*(2,6) = 3512.77, *P* < 0.0001] and concentration [*F*(1.006,6.033) = 25 952.53, *P* < 0.0001], together with a significant treatment × concentration interaction [*F*(2.011,6.033) = 4153.69, *P* < 0.0001]. The significant interaction indicated that the magnitude of the concentration-dependent increase differed among treatments. Tukey-adjusted multiple comparisons identified significant differences among treatments at each tested concentration (*P* < 0.05), as indicated by different lowercase letters in Fig. 11D.

Because neither nanoparticle system reached 50% inhibition, their responses were compared using the measured inhibition percentages rather than IC_50_-based potency ratios. Multi-REO NPs exhibited a stronger apparent HO˙-scavenging response than CeO_2_ NPs under the applied cell-free assay conditions, whereas ascorbic acid remained the most active treatment. No reference-to-multi-REO IC_50_ ratio was calculated because the multi-REO response did not reach 50% within the tested range.

##### 3.9.1.5. H_2_O_2_ scavenging (Fig. 11E)

In the hydrogen peroxide-scavenging assay, all treatments exhibited concentration-dependent increases in scavenging response, with multi-REO NPs consistently producing greater inhibition than CeO_2_ NPs at equivalent concentrations. At 200 µg mL^−1^, multi-REO NPs achieved 55.82 ± 1.22% inhibition, compared with 11.01 ± 0.80% for CeO_2_ NPs and 74.53 ± 0.67% for ascorbic acid. Data are presented as mean ± SD across three independent experimental units (*n* = 3).

Constrained nonlinear regression, with the lower and upper response plateaus fixed at 0% and 100%, respectively, and the Hill slope fixed at 1, yielded an estimated IC_50_ of 173.0 µg mL^−1^ for multi-REO NPs (95% CI: 161.9–185.1 µg mL^−1^; *R*^2^ = 0.983) and 100.8 µg mL^−1^ for ascorbic acid (95% CI: 83.0–122.3 µg mL^−1^; *R*^2^ = 0.911). CeO_2_ NPs did not reach 50% inhibition within the tested concentration range of 50–200 µg mL^−1^; therefore, the corresponding IC_50_ endpoint was designated as not reached within the tested range (NR), and no extrapolated numerical IC_50_ estimate or confidence interval was reported.

Two-way mixed-design repeated-measures ANOVA, with concentration treated as the within-unit repeated factor and using the Geisser–Greenhouse correction, revealed significant effects of treatment [*F*(2,6) = 3686.18, *P* < 0.0001]and concentration [*F*(1.003,6.019) = 23 405.96, *P* < 0.0001], together with a significant treatment × concentration interaction [*F*(2.006,6.019) = 3742.09, *P* < 0.0001]. The significant interaction indicated that the magnitude of the concentration-dependent increase differed among treatments. Tukey-adjusted multiple comparisons identified significant differences among treatments at each tested concentration (*P* < 0.05), as indicated by different lowercase letters in Fig. 11E.

The estimated IC_50_ of multi-REO NPs was located toward the upper end of the tested concentration range and should therefore be interpreted cautiously. Overall, multi-REO NPs exhibited a stronger apparent H_2_O_2_-scavenging response than CeO_2_ NPs under the applied cell-free assay conditions but remained less active than ascorbic acid. The estimated reference-to-multi-REO IC_50_ ratio was 0.58, indicating that multi-REO NPs required a higher concentration than ascorbic acid to achieve 50% H_2_O_2_ scavenging. No IC_50_-based ratio was calculated for CeO_2_ NPs because 50% inhibition was not reached within the tested range. These comparisons are assay-specific descriptors and should not be interpreted as evidence of therapeutic potency.

#### 3.9.2. Total antioxidant capacity and reducing power

The total antioxidant capacity (TAC) and iron reducing power (IRP) assays were used to further compare the overall redox-related behavior of CeO_2_ NPs and multi-REO NPs (Fig. 11G and H). Both assays showed concentration-dependent responses, with multi-REO NPs consistently producing higher values than CeO_2_ NPs at equivalent concentrations.

##### 3.9.2.1. Total antioxidant capacity (TAC) (Fig. 11G)

Total antioxidant capacity, expressed as milligrams of gallic acid equivalents per gram of sample (mg GAE g^−1^), increased with concentration in all treatments. At 50 µg mL^−1^, TAC values were 35.78 ± 0.26, 28.94 ± 0.58, and 12.57 ± 0.34 mg GAE g^−1^ for ascorbic acid, multi-REO NPs, and CeO_2_ NPs, respectively. At 100 µg mL^−1^, the corresponding values increased to 59.03 ± 0.38, 47.75 ± 0.83, and 20.74 ± 0.53 mg GAE g^−1^. At 200 µg mL^−1^, ascorbic acid produced the highest TAC response (93.85 ± 0.61 mg GAE g^−1^), followed by multi-REO NPs (75.92 ± 1.40 mg GAE g^−1^) and CeO_2_ NPs (32.98 ± 0.83 mg GAE g^−1^). Data are presented as mean ± SD across three independent experimental units (*n* = 3).

Two-way mixed-design repeated-measures ANOVA, with treatment analyzed as the between-unit factor and concentration as the within-unit repeated factor, revealed significant effects of treatment [*F*(2,6) = 2907.12, *P* < 0.0001] and concentration [*F*(1.057,6.344) = 42 337.53, *P* < 0.0001], together with a significant treatment × concentration interaction [*F*(2.115,6.344) = 3021.96, *P* < 0.0001]. Sphericity was not assumed, and the Geisser–Greenhouse correction was applied to the concentration and interaction effects. The significant interaction indicated that the magnitude of the concentration-dependent increase differed among treatments.

Tukey-adjusted multiple comparisons showed that multi-REO NPs produced significantly greater TAC values than CeO_2_ NPs at each tested concentration (all adjusted *P* < 0.0001). Ascorbic acid also produced significantly greater TAC values than multi-REO NPs at 50 µg mL^−1^ (adjusted *P* < 0.01) and at 100 and 200 µg mL^−1^ (adjusted *P* < 0.001). Overall, multi-REO NPs exhibited a substantially greater concentration-dependent TAC response than CeO_2_ NPs under the applied cell-free assay conditions, although the response remained lower than that of ascorbic acid.

##### 3.9.2.2. Iron reducing power (Fig. 11H)

Iron reducing power, used as an indicator of apparent electron-donating capacity under the applied cell-free assay conditions, increased with concentration in all treatments. At 50 µg mL^−1^, IRP values were 28.62 ± 0.29, 21.79 ± 0.52, and 5.42 ± 0.29 µg mL^−1^ for ascorbic acid, multi-REO NPs, and CeO_2_ NPs, respectively. At 100 µg mL^−1^, the corresponding values increased to 47.23 ± 0.38, 35.95 ± 0.74, and 8.94 ± 0.48 µg mL^−1^. At 200 µg mL^−1^, ascorbic acid produced the highest IRP response (75.09 ± 0.54 µg mL^−1^), followed by multi-REO NPs (57.17 ± 1.31 µg mL^−1^) and CeO_2_ NPs (14.22 ± 0.75 µg mL^−1^). Data are presented as mean ± SD across three independent experimental units (*n* = 3).

Two-way mixed-design repeated-measures ANOVA, with treatment analyzed as the between-unit factor and concentration as the within-unit repeated factor, revealed significant effects of treatment [*F*(2,6) = 3453.70, *P* < 0.0001] and concentration [*F*(1.039,6.235) = 25 726.58, *P* < 0.0001], together with a significant treatment × concentration interaction [*F*(2.078,6.235) = 3519.57, *P* < 0.0001]. Sphericity was not assumed, and the Geisser–Greenhouse correction was applied to the concentration and interaction effects. The significant interaction indicated that the magnitude of the concentration-dependent increase differed among treatments.

Tukey-adjusted multiple comparisons showed significant differences among the three treatments at each tested concentration (all adjusted *P* < 0.0001). Multi-REO NPs produced consistently greater reducing-power responses than CeO_2_ NPs, with multi-REO/CeO_2_ response ratios of approximately 4.02 at 50, 100, and 200 µg mL^−1^. However, the response of multi-REO NPs remained lower than that of ascorbic acid at all tested concentrations.

Overall, the IRP results demonstrated a greater apparent electron-donating response for multi-REO NPs than for CeO_2_ NPs under the applied assay conditions. This pattern was consistent with the relative ordering observed in the total antioxidant-capacity and radical- or reactive-species-scavenging assays. Nevertheless, the IRP assay represents an assay-specific cell-free redox response and should not be interpreted as direct evidence of intracellular antioxidant activity or therapeutic efficacy.

The measured radical- and reactive-species-scavenging responses may arise from several concurrent contributions, including adsorption of reactive species at the nanoparticle interface, electron- or hydrogen-transfer reactions involving surface-associated constituents, and reactions at hydroxylated or defect-related surface sites. Differences between the CeO_2_ and multi-REO samples may additionally reflect variations in elemental composition, local coordination environments, particle morphology, aggregation state, surface charge, and extract-derived surface functionalization. Therefore, the observed responses should not be assigned to a single mechanism or exclusively to the inorganic nanoparticle cores.

For Ce-containing nanomaterials, reversible changes between Ce^3+^- and Ce^4+^-containing surface environments are frequently proposed to contribute to ROS modulation through coupled electron-transfer and oxygen-storage/release processes. Such redox behavior may support reactions with superoxide- and peroxide-related species, although the direction and magnitude of the response depend on the Ce^3+^/Ce^4+^ ratio, oxygen-defect chemistry, surface coating, pH, and reaction medium. Because the Ce^3+^/Ce^4+^ distribution and oxygen-vacancy concentration were not directly quantified in the present samples, redox cycling is considered a plausible literature-supported contribution rather than a mechanism established directly by the current data.

Targeted HPLC-DAD profiling confirmed selected phenolic and flavonoid constituents in the aqueous *M. oleifera* seed extract, and FTIR analysis indicated extract-derived surface-associated functionalities in the synthesized materials. These constituents may contribute to radical quenching through hydrogen- or electron-transfer pathways and may modify nanoparticle–reactive-species interactions. However, because an extract-only control was not included in the antioxidant assays, the contribution of residual surface-associated phytochemicals cannot be quantitatively separated from that of the inorganic nanoparticle components. The results are therefore interpreted as preliminary scavenging profiles of the complete biogenic nanoparticle systems.

#### 3.9.3. Enzyme- and protein-based assays: preliminary cell-free screening

In addition to the redox-related assays, the biosynthesized CeO_2_ NPs and multi-REO NPs were evaluated using selected preliminary cell-free enzyme- and protein-based screening models. These included AChE inhibition, BSA thermal-denaturation inhibition, and α-amylase and α-glucosidase inhibition over the concentration range of 50–200 µg mL^−1^ (Fig. 12–14). These assays were used to provide comparative biochemical response profiles and should not be interpreted as direct evidence of neuroprotective, anti-inflammatory, antidiabetic, or therapeutic efficacy.

##### 3.9.3.1. Acetylcholinesterase (AChE) inhibition

The AChE inhibition assay was used as a preliminary cell-free screening model to compare the responses of CeO_2_ NPs and multi-REO NPs with donepezil as the reference inhibitor. Within the tested concentration range of 50–200 µg mL^−1^, donepezil exhibited a clear concentration-dependent increase in AChE inhibition. The response increased from 22.24 ± 0.17% at 50 µg mL^−1^ to 42.04 ± 0.32% at 100 µg mL^−1^ and 79.45 ± 0.59% at 200 µg mL^−1^. Data are presented as mean ± SD across three independent experimental units (*n* = 3).

Constrained nonlinear regression, with the lower and upper response plateaus fixed at 0% and 100%, respectively, and the Hill slope fixed at 1, yielded an estimated IC_50_ of 109.4 µg mL^−1^ for donepezil (95% CI: 76.8–156.5 µg mL^−1^; *R*^2^ = 0.801). Because the estimate was derived from only three tested concentrations and had a relatively broad confidence interval, it was interpreted as a model-dependent estimate of the reference response.

In contrast, both nanoparticle systems produced low AChE inhibition values within a narrow range and did not exhibit monotonic concentration-dependent responses. Multi-REO NPs produced inhibition values of 19.11 ± 0.14%, 20.45 ± 0.15%, and 19.28 ± 0.14% at 50, 100, and 200 µg mL^−1^, respectively. CeO_2_ NPs produced corresponding values of 20.11 ± 0.14%, 21.52 ± 0.15%, and 20.29 ± 0.14%. For both nanoparticle systems, inhibition increased slightly at 100 µg mL^−1^ and subsequently decreased at 200 µg mL^−1^. Thus, although small numerical differences were observed among concentrations, the patterns did not constitute consistent monotonic concentration-dependent responses.

Two-way mixed-design repeated-measures ANOVA, with treatment analyzed as the between-unit factor and concentration as the within-unit repeated factor, revealed significant effects of treatment [*F*(2,6) = 13 388.47, *P* < 0.0001] and concentration [*F*(1.003,6.017) = 53 963.88, *P* < 0.0001], together with a significant treatment × concentration interaction [*F*(2.006,6.017) = 54 347.59, *P* < 0.0001]. Sphericity was not assumed, and the Geisser–Greenhouse correction was applied to the concentration and interaction effects (*ε*_GG_ = 0.5015). The significant interaction indicated that the effect of concentration differed among treatments, primarily because donepezil exhibited a pronounced concentration-dependent increase, whereas the nanoparticle responses remained low and non-monotonic.

Tukey-adjusted multiple comparisons identified significant differences among the treatments at each tested concentration (*P* < 0.05), as indicated by different lowercase letters in Fig. 12. Donepezil produced significantly greater inhibition than both nanoparticle systems at each concentration. Although CeO_2_ NPs and multi-REO NPs also differed statistically, the absolute difference between their means was approximately one percentage point at each concentration and was therefore small in magnitude. Statistical significance in this comparison should not be interpreted as evidence of a biologically meaningful difference.

**Fig. 12:**
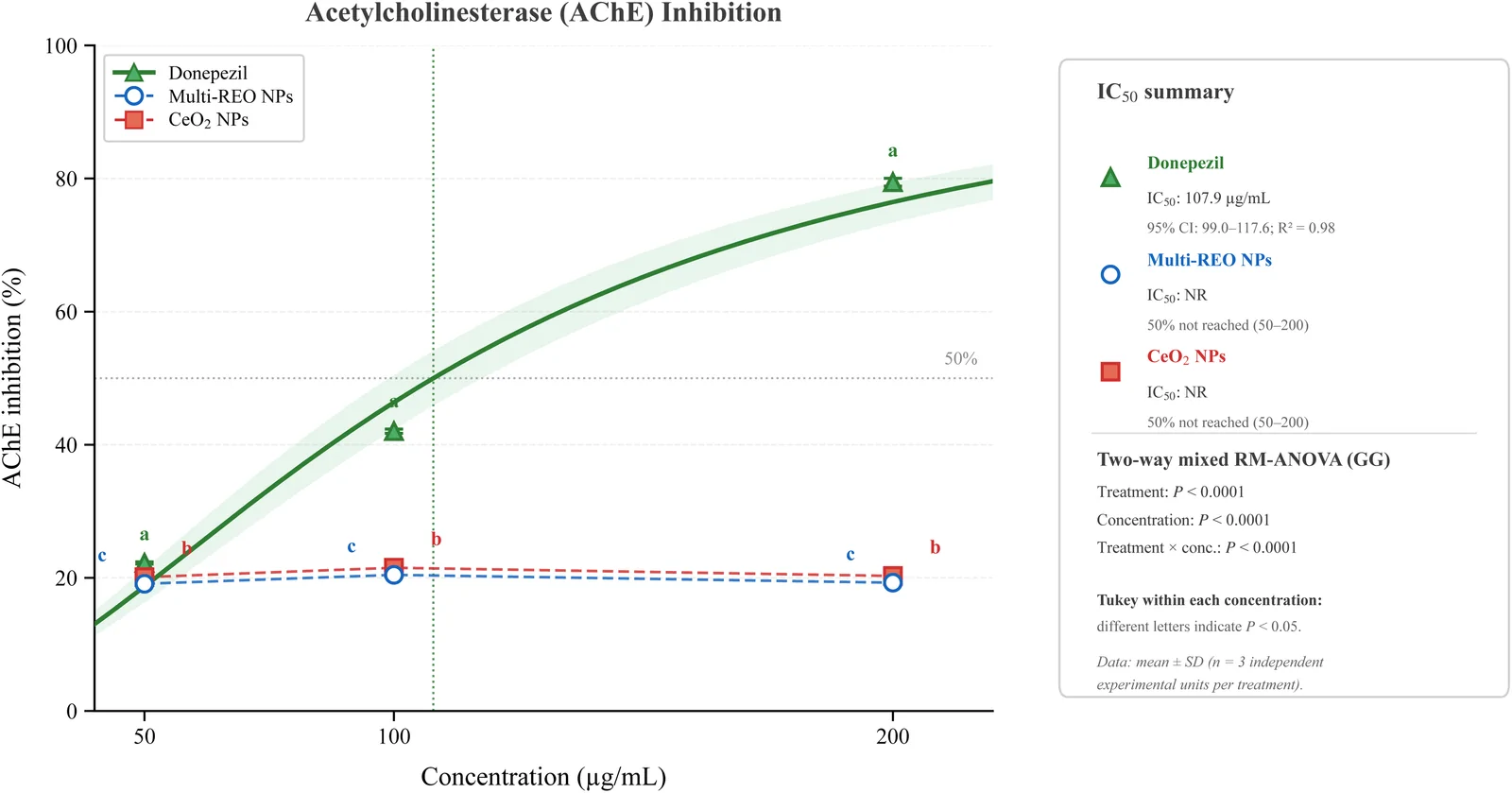
Acetylcholinesterase (AChE)-inhibitory responses of biosynthesized CeO_2_ NPs and multi-REO NPs compared with donepezil as the reference inhibitor. Donepezil exhibited a concentration-dependent increase in AChE inhibition over the tested concentration range of 50–200 µg mL^−1^. Constrained nonlinear regression yielded an estimated IC_50_ of 109.4 µg mL^−1^ for donepezil (95% CI: 76.8–156.5 µg mL^−1^; *R*^2^ = 0.801). In contrast, CeO_2_ NPs and multi-REO NPs produced low apparent inhibition values of approximately 19–22% without monotonic concentration-dependent responses; consequently, their IC_50_ values were designated as not determined (N.D.). Data are presented as mean ± SD across three independently prepared synthesis batches for each nanoparticle system and three independent assay runs for donepezil (*n* = 3 independent experimental units per treatment). Treatment, concentration, and the treatment × concentration interaction were evaluated using two-way mixed-design repeated-measures ANOVA, with concentration analyzed as the within-unit repeated factor and the Geisser–Greenhouse correction applied to the concentration and interaction effects. Different lowercase letters indicate significant differences among treatments within the same concentration according to Tukey-adjusted multiple comparisons (*P* < 0.05). N.D., not determined.

Reliable IC_50_ values could not be determined for either nanoparticle system because neither CeO_2_ NPs nor multi-REO NPs approached 50% AChE inhibition within the tested range, and their non-monotonic responses did not support interpretable sigmoidal concentration-response fitting. Their IC_50_ values were therefore designated as not determined (N.D.). Overall, the results indicated low apparent AChE inhibition by both nanoparticle systems under the applied cell-free assay conditions and did not support the assignment of conventional concentration-dependent AChE-inhibitory potency, a specific inhibition mechanism, or neuroprotective relevance.

The low and non-monotonic responses may reflect weak nanoparticle–enzyme interactions, limited accessibility of relevant enzyme-binding regions, or assay-related effects. However, no specific binding or inhibition mechanism can be established from the present endpoint assay alone. Although the relatively narrow active-site gorge of AChE could potentially influence interactions with nanoparticulate species, direct restriction of nanoparticle access to the catalytic site was not demonstrated in the present study and should therefore be considered only as a possible explanation.^58,59^

Compared with the carbohydrate-hydrolyzing enzyme assays described below, the AChE findings demonstrate that the preliminary cell-free response profile of the multi-REO material was assay- and target-dependent. Multi-REO NPs did not produce greater AChE inhibition than CeO_2_ NPs under the applied conditions, despite producing greater responses in the α-amylase and α-glucosidase assays. This difference may reflect target-specific enzyme structure, nanoparticle–protein interfacial interactions, aggregation, surface-associated constituents, or assay-related effects. However, the present endpoint data do not establish a specific explanation for the different response patterns.^60,61^

Overall, neither nanoparticle system produced a monotonic concentration-dependent AChE-inhibitory response over 50–200 µg mL^−1^. The findings should therefore be regarded as negative or inconclusive preliminary screening results rather than evidence of appreciable cholinesterase inhibition. Claims concerning AChE inhibition, specific molecular mechanisms, or neuroprotective relevance would require confirmation using additional independent synthesis batches and independent assay runs, an expanded concentration range, enzyme-kinetic analysis, additional cholinesterase targets, orthogonal activity assays, and appropriate nanoparticle-interference controls.

##### 3.9.3.2. BSA protein denaturation inhibition

The inhibition of heat-induced bovine serum albumin (BSA) denaturation was evaluated as a preliminary cell-free protein-denaturation screening model to compare the responses of CeO_2_ NPs and multi-REO NPs with diclofenac sodium as the reference compound. Although this assay is commonly used as a preliminary indicator of protein stabilization, the measured response should not be interpreted as direct evidence of anti-inflammatory or anti-arthritic efficacy.

All three treatments exhibited monotonic concentration-dependent increases in BSA thermal-denaturation inhibition over the tested concentration range of 50–200 µg mL^−1^. Diclofenac sodium produced inhibition values of 25.58 ± 0.28%, 38.37 ± 0.42%, and 72.51 ± 0.80% at 50, 100, and 200 µg mL^−1^, respectively. Multi-REO NPs produced corresponding inhibition values of 21.98 ± 0.66%, 32.97 ± 0.99%, and 62.31 ± 1.87%, whereas CeO_2_ NPs produced substantially lower values of 7.80 ± 0.38%, 11.69 ± 0.56%, and 22.10 ± 1.07%, respectively. Data are presented as mean ± SD across three independent experimental units (*n* = 3).

Constrained nonlinear regression, with the lower and upper response plateaus fixed at 0% and 100%, respectively, and the Hill slope fixed at 1, yielded an estimated IC_50_ of 159.6 µg mL^−1^ for multi-REO NPs (95% CI: 132.7–193.1 µg mL^−1^; *R*^2^ = 0.906) and 121.5 µg mL^−1^ for diclofenac sodium (95% CI: 94.3–157.1 µg mL^−1^; *R*^2^ = 0.860). CeO_2_ NPs did not reach 50% inhibition within the tested concentration range; therefore, the corresponding IC_50_ endpoint was designated as not reached within the tested range (NR), and no extrapolated numerical IC_50_ estimate or confidence interval was reported.

The estimated IC_50_ of multi-REO NPs was 38.1 µg mL^−1^ higher than that of diclofenac sodium, indicating a lower apparent BSA denaturation-inhibitory response than the reference compound. The reference-to-multi-REO IC_50_ ratio was 0.76. This ratio is an assay-specific comparative descriptor and does not establish statistical equivalence or comparable therapeutic potency. The 95% confidence intervals of the two IC_50_ estimates overlapped; therefore, the present estimates alone do not demonstrate a statistically significant difference between the fitted IC_50_ values without a direct comparison of the fitted curves or log IC_50_ parameters.

Two-way mixed-design repeated-measures ANOVA, with treatment analyzed as the between-unit factor and concentration as the within-unit repeated factor, revealed significant effects of treatment [*F*(2,6) = 1207.69, *P* < 0.0001]and concentration [*F*(1.000,6.000) = 13 956.92, *P* < 0.0001], together with a significant treatment × concentration interaction [*F*(2.000,6.000) = 1207.89, *P* < 0.0001]. Sphericity was not assumed, and the Geisser–Greenhouse correction was applied to the concentration and interaction effects (*ε*_GG_ = 0.5000). The significant interaction indicated that the magnitude of the concentration-dependent increase differed among treatments, with substantially larger increases observed for diclofenac sodium and multi-REO NPs than for CeO_2_ NPs.

Tukey-adjusted multiple comparisons identified significant differences among the three treatments at each tested concentration. Diclofenac sodium produced significantly greater inhibition than multi-REO NPs at 50, 100, and 200 µg mL^−1^, with adjusted *P*-values of 0.0095, 0.0097, and 0.0095, respectively. Multi-REO NPs produced significantly greater inhibition than CeO_2_ NPs at all three concentrations (all adjusted *P* < 0.0001), and CeO_2_ NPs produced significantly lower inhibition than diclofenac sodium at every concentration (all adjusted *P* < 0.0001). These comparisons are represented in Fig. 13 by different lowercase letters assigned to treatments within the same concentration.

**Fig. 13:**
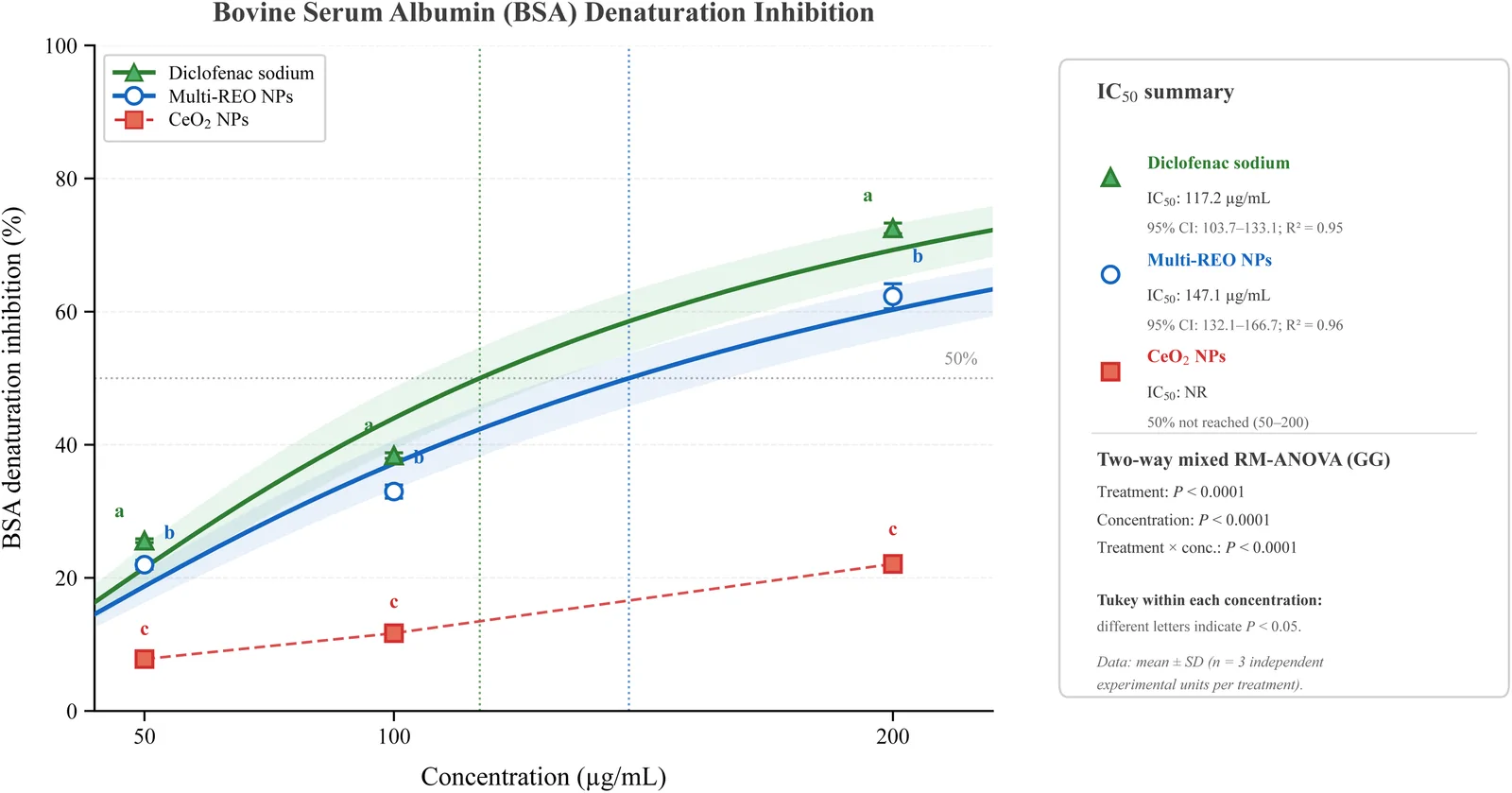
BSA thermal-denaturation-inhibitory responses of biosynthesized CeO_2_ NPs and multi-REO NPs compared with diclofenac sodium as the reference compound over the tested concentration range of 50–200 µg mL^−1^. Constrained nonlinear regression yielded estimated IC_50_ values of 159.6 µg mL^−1^ for multi-REO NPs (95% CI: 132.7–193.1 µg mL^−1^; *R*^2^ = 0.906) and 121.5 µg mL^−1^ for diclofenac sodium (95% CI: 94.3–157.1 µg mL^−1^; *R*^2^ = 0.860). CeO_2_ NPs did not reach 50% inhibition within the tested range, and the corresponding IC_50_ endpoint was therefore designated as NR. Lines connecting the CeO_2_ NP means are descriptive and do not represent a fitted concentration–response model. Data are presented as mean ± SD across three independently prepared synthesis batches for each nanoparticle treatment and three independent assay runs for diclofenac sodium (*n* = 3 independent experimental units per treatment). Treatment, concentration, and the treatment × concentration interaction were evaluated using two-way mixed-design repeated-measures ANOVA, with the Geisser–Greenhouse correction applied to the concentration and interaction effects. Different lowercase letters indicate significant differences among treatments within the same concentration according to Tukey-adjusted multiple comparisons (*P* < 0.05). The horizontal dashed line denotes 50% inhibition. NR, 50% response not reached within the tested concentration range.

Overall, multi-REO NPs exhibited a stronger apparent BSA thermal-denaturation-inhibitory response than CeO_2_ NPs, although their response remained lower than that of diclofenac sodium. The difference between the nanoparticle systems may be associated with variations in composition, phase complexity, surface chemistry, aggregation, surface-associated extract constituents, or nanoparticle–protein interfacial interactions. However, the present assay does not establish that the difference resulted specifically from rare-earth co-incorporation, oxygen vacancies, or stabilization of the native BSA conformation.

The measured endpoint may reflect one or more concurrent effects on protein unfolding, association, aggregation, turbidity, adsorption, or nanoparticle-related optical and colloidal interference. Accordingly, no specific molecular mechanism can be assigned from this assay alone. Further evaluation using additional independent synthesis batches, nanoparticle-interference controls, protein-binding measurements, orthogonal protein-structure or aggregation assays, and appropriately designed cell-based inflammatory models would be required to establish the reproducibility, mechanism, and biological relevance of the observed response.

The BSA thermal-denaturation assay is therefore interpreted as a preliminary cell-free protein-based screening model. The observed response should not be considered direct evidence of anti-inflammatory or anti-arthritic efficacy. Nanoparticle-interference controls, protein-binding measurements, zeta-potential analysis, orthogonal protein-structure measurements, and appropriately designed cell-based inflammatory assays would be required to confirm the biological relevance and underlying mechanism of the observed response.

##### 3.9.3.3. Carbohydrate-hydrolyzing enzyme inhibition assays

According to the 2021 estimates reported by the International Diabetes Federation, approximately 536.6 million adults aged 20–79 years were living with diabetes worldwide, with the number projected to reach 783.2 million by 2045.^62^ Postprandial carbohydrate digestion is partly mediated by α-amylase, which hydrolyzes starch into smaller oligosaccharides, and α-glucosidase, which catalyzes the terminal hydrolysis of carbohydrate residues to produce absorbable monosaccharides. Inhibition of these enzymes is therefore commonly employed as a preliminary cell-free screening approach related to the modulation of carbohydrate digestion.

The inhibitory responses of CeO_2_ NPs and multi-REO NPs against α-amylase and α-glucosidase were evaluated over a concentration range of 50–200 µg mL^−1^, using acarbose as the reference inhibitor. These assays were interpreted as preliminary enzyme-level cell-free screening models and not as direct evidence of antidiabetic or therapeutic efficacy.

###### 3.9.3.3.1. α-Amylase inhibition

In the α-amylase inhibition assay (Fig. 14A), all three treatments exhibited concentration-dependent increases in enzyme inhibition. Multi-REO NPs produced inhibition values of 25.28 ± 0.77%, 40.46 ± 0.00%, and 66.31 ± 2.00% at 50, 100, and 200 µg mL^−1^, respectively. CeO_2_ NPs exhibited a weaker response, producing corresponding inhibition values of 9.69 ± 0.47%, 15.32 ± 0.00%, and 25.41 ± 1.23%. Acarbose produced the greatest inhibition at each concentration, with values of 31.78 ± 0.35%, 52.45 ± 0.58%, and 83.39 ± 0.92%, respectively. Data are presented as mean ± SD across three independent experimental units (*n* = 3).

**Fig. 14:**
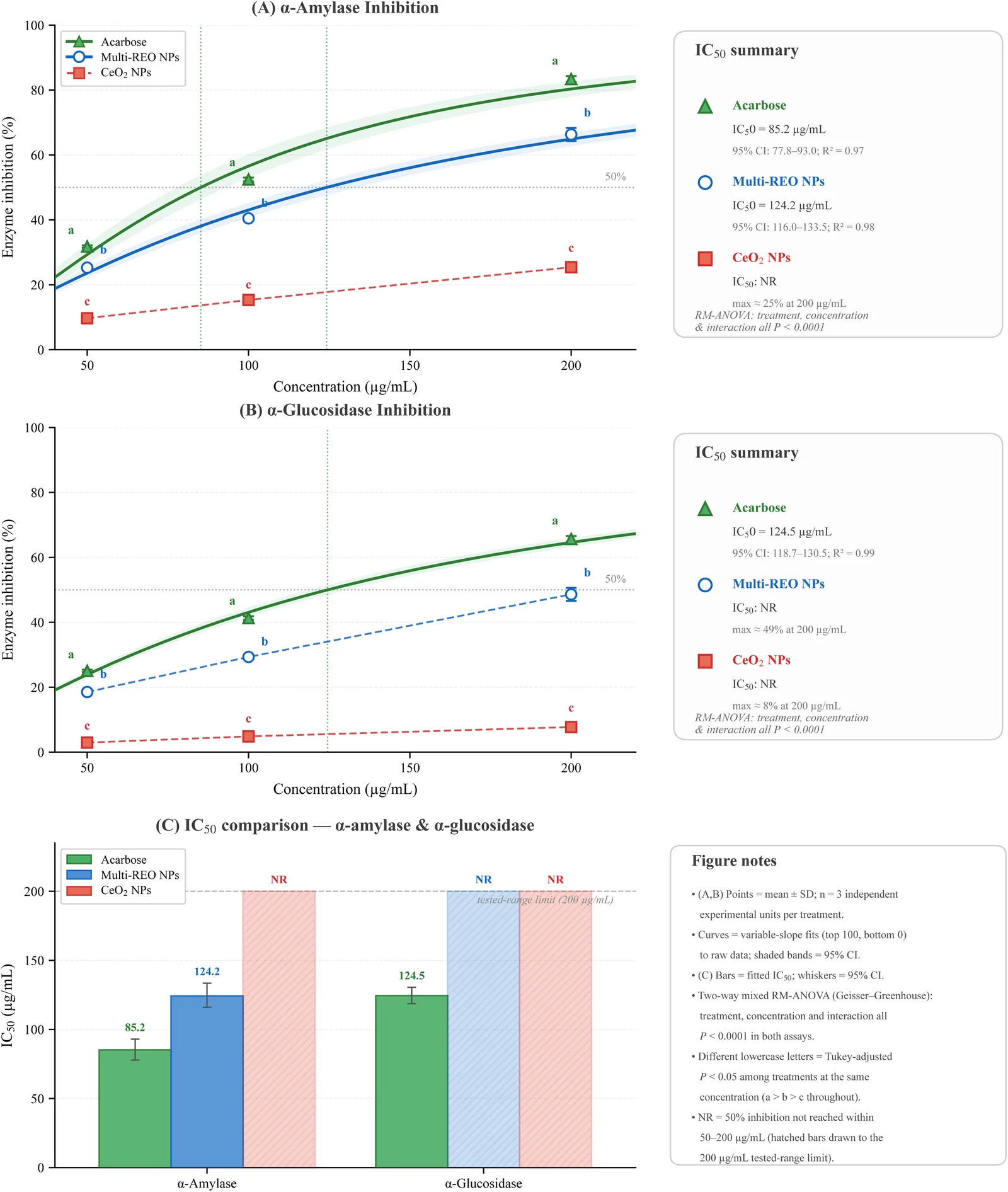
Carbohydrate-hydrolyzing enzyme-inhibitory responses of biosynthesized CeO_2_ NPs and multi-REO NPs compared with acarbose as the reference inhibitor. Concentration-dependent inhibitory responses against (A) α-amylase and (B) α-glucosidase were evaluated over 50–200 µg mL^−1^. For α-amylase, constrained nonlinear regression yielded estimated IC_50_ values of 129.1 µg mL^−1^ for multi-REO NPs (95% CI: 111.9–149.1 µg mL^−1^; *R*^2^ = 0.9411) and 79.57 µg mL^−1^ for acarbose (95% CI: 60.06–104.8 µg mL^−1^; *R*^2^ = 0.8533). CeO_2_ NPs did not reach 50% α-amylase inhibition within the tested range, and the corresponding IC_50_ endpoint was therefore designated as NR. For α-glucosidase, acarbose exhibited an estimated IC_50_ of 129.0 µg mL^−1^ (95% CI: 113.9–146.3 µg mL^−1^), whereas neither multi-REO NPs nor CeO_2_ NPs reached 50% inhibition within the tested range; their corresponding IC_50_ endpoints were therefore designated as NR. (C) Summary of the estimated IC_50_ values and endpoints at which 50% inhibition was not reached. Hatched bars marked NR and terminating at 200 µg mL^−1^ indicate the upper tested concentration and do not represent measured or estimated IC_50_ values of 200 µg mL^−1^. The horizontal dashed line indicates 50% inhibition. Solid curves represent valid constrained concentration-response fits, whereas lines connecting means for NR endpoints are descriptive only and do not represent fitted concentration-response models. Shaded regions illustrate model-based uncertainty derived from the profile-likelihood 95% confidence limits of the corresponding estimable IC_50_ values. Data are presented as mean ± SD across three independently prepared synthesis batches for each nanoparticle system and three independent assay runs for acarbose (*n* = 3 independent experimental units per treatment). Treatment, concentration, and the treatment × concentration interaction were evaluated using two-way mixed-design repeated-measures ANOVA, with treatment analyzed as the between-unit factor and concentration as the within-unit repeated factor. Sphericity was not assumed, and the Geisser–Greenhouse correction was applied to the concentration and interaction effects. Different lowercase letters indicate significant differences among treatments within the same concentration according to Tukey-adjusted multiple comparisons (*P* < 0.05). NR, 50% response not reached within the tested concentration range.

Constrained nonlinear regression, with the lower and upper response plateaus fixed at 0% and 100%, respectively, and the Hill slope fixed at 1, yielded an estimated IC_50_ of 129.1 µg mL^−1^ for multi-REO NPs (95% CI: 111.9–149.1 µg mL^−1^; *R*^2^ = 0.9411) and 79.57 µg mL^−1^ for acarbose (95% CI: 60.06–104.8 µg mL^−1^; *R*^2^ = 0.8533). CeO_2_ NPs did not reach 50% inhibition within the tested concentration range of 50–200 µg mL^−1^. The corresponding IC_50_ endpoint was therefore designated as not reached within the tested range (NR), and no extrapolated numerical IC_50_ estimate or confidence interval was reported.

The estimated IC_50_ of multi-REO NPs was higher than that of acarbose, indicating a lower apparent α-amylase-inhibitory response than the reference inhibitor. The reference-to-multi-REO IC_50_ ratio was 0.62. This ratio is an assay-specific comparative descriptor and should not be interpreted as evidence of therapeutic potency or statistical equivalence.

Two-way mixed-design repeated-measures ANOVA, with treatment analyzed as the between-unit factor and concentration as the within-unit repeated factor, revealed significant effects of treatment [*F*(2,6) = 2338.68, *P* < 0.0001] and concentration [*F*(1.057,6.343) = 6036.93, *P* < 0.0001], together with a significant treatment × concentration interaction [*F*(2.114,6.343) = 521.01, *P* < 0.0001]. Sphericity was not assumed, and the Geisser–Greenhouse correction was applied to the concentration and interaction effects. The significant interaction indicated that the magnitude of the concentration-dependent increase differed among treatments.

Tukey-adjusted multiple comparisons identified significant differences among treatments at each tested concentration (*P* < 0.05), as indicated by different lowercase letters in Fig. 14A. Overall, multi-REO NPs exhibited a stronger apparent α-amylase-inhibitory response than CeO_2_ NPs but remained less active than acarbose under the applied cell-free assay conditions. The findings should be interpreted as preliminary enzyme-level screening responses rather than evidence of antidiabetic or therapeutic efficacy.

###### 3.9.3.3.2. α-Glucosidase inhibition

In the α-glucosidase inhibition assay (Fig. 14B), all treatments exhibited concentration-dependent increases in enzyme inhibition. Multi-REO NPs produced inhibition values of 18.53 ± 0.77%, 29.33 ± 0.00%, and 48.60 ± 2.00% at 50, 100, and 200 µg mL^−1^, respectively. Although the response approached 50% at 200 µg mL^−1^, it did not reach 50% within the tested concentration range. Therefore, the corresponding IC_50_ endpoint was designated as not reached within the tested range (NR), and no extrapolated numerical IC_50_ estimate or confidence interval was reported.

CeO_2_ NPs exhibited substantially weaker α-glucosidase inhibition, producing values of 2.94 ± 0.47%, 4.84 ± 0.77%, and 7.70 ± 1.22% at 50, 100, and 200 µg mL^−1^, respectively. Because 50% inhibition was not reached within the tested range, the corresponding IC_50_ endpoint was also designated as NR.

Acarbose produced the greatest inhibition at each concentration, with values of 25.03 ± 0.35%, 41.31 ± 0.58%, and 65.68 ± 0.92% at 50, 100, and 200 µg mL^−1^, respectively. Data are presented as mean ± SD across three independent experimental units (*n* = 3).

Constrained nonlinear regression, with the lower and upper response plateaus fixed at 0% and 100%, respectively, and the Hill slope fixed at 1, yielded an estimated IC_50_ of 129.0 µg mL^−1^ for acarbose (95% CI: 113.9–146.3 µg mL^−1^). The acarbose response reached and exceeded 50% inhibition within the tested concentration range, supporting estimation of its IC_50_. Because neither nanoparticle system reached 50% inhibition, no IC_50_-based potency ratios were calculated for multi-REO NPs or CeO_2_ NPs.

Two-way mixed-design repeated-measures ANOVA, with treatment analyzed as the between-unit factor and concentration as the within-unit repeated factor, revealed significant effects of treatment [*F*(2,6) = 1876.95, *P* < 0.0001]and concentration [*F*(1.182,7.094) = 3484.53, *P* < 0.0001], together with a significant treatment × concentration interaction [*F*(2.365,7.094) = 623.52, *P* < 0.0001]. Sphericity was not assumed, and the Geisser–Greenhouse correction was applied to the concentration and interaction effects. The significant interaction indicated that the magnitude of the concentration-dependent increase differed among acarbose, multi-REO NPs, and CeO_2_ NPs.

Tukey-adjusted multiple comparisons identified significant differences among treatments at each tested concentration (*P* < 0.05), as indicated by different lowercase letters in Fig. 14B. Overall, multi-REO NPs exhibited a stronger apparent α-glucosidase-inhibitory response than CeO_2_ NPs but remained less active than acarbose under the applied cell-free assay conditions.

The observed differences may be associated with precursor chemistry, elemental composition, phase complexity, surface chemistry, aggregation, surface-associated extract constituents, or nanoparticle–enzyme interfacial interactions. However, the present endpoint assay does not distinguish among active-site inhibition, allosteric effects, enzyme or substrate adsorption, conformational changes, colloidal interactions, and optical or colorimetric interference. The findings should therefore be interpreted as preliminary enzyme-level screening responses rather than evidence of antidiabetic or therapeutic efficacy.

Previous studies have reported that plant-mediated metal oxide nanoparticles can inhibit carbohydrate-hydrolyzing enzymes. For example, pomegranate-husk-mediated ZnO nanoparticles were reported to exhibit substantial α-amylase inhibition under the conditions of that study, supporting the general possibility that phytochemical-associated nanoparticle surfaces may influence enzyme activity. Nevertheless, differences in nanoparticle composition, synthesis route, surface chemistry, particle size, dispersion state, and assay conditions preclude direct quantitative comparison with the present rare-earth oxide systems.^16^

Further investigations using enzyme-kinetic measurements at multiple substrate concentrations, substrate-competition analysis, nanoparticle-only blanks, enzyme-free and substrate-free controls, nanoparticle–enzyme binding measurements, and orthogonal activity assays would be required to establish the inhibition mode and biological relevance of the observed responses.

### 3.10. Integrated comparison of physicochemical properties and cell-free screening responses

The multi-REO and CeO_2_ nanoparticle systems differed in morphology, apparent crystallographic ordering, aggregation behavior, surface chemistry, and colloidal properties. Under the applied experimental conditions, multi-REO NPs generally produced greater apparent responses than CeO_2_ NPs in the radical- and reactive-species-scavenging assays, BSA thermal-denaturation assay, and carbohydrate-hydrolyzing enzyme-inhibition assays. However, this pattern was assay-specific and was not observed as a monotonic concentration-dependent response in the AChE assay.

Neither nanoparticle system exhibited a monotonic concentration-dependent AChE-inhibitory response. Instead, both systems produced low apparent inhibition values of approximately 19–22% over the tested concentration range, with a slight increase at 100 µg mL^−1^ followed by a decrease at 200 µg mL^−1^. Consequently, reliable AChE IC_50_ values could not be determined for either nanoparticle system and were designated as N.D.

For several endpoints, CeO_2_ NPs did not reach 50% response within the tested concentration range of 50–200 µg mL^−1^. Multi-REO NPs also did not reach 50% response in the HO˙-scavenging and α-glucosidase-inhibition assays. These endpoints were designated as not reached within the tested range (NR), and no extrapolated numerical IC_50_ estimates or confidence intervals were reported. Endpoints designated as NR were excluded from IC_50_ ratio-based comparisons.

The generally greater apparent assay responses observed for multi-REO NPs may be associated with differences in precursor chemistry, elemental composition, phase complexity, apparent crystallographic ordering, particle morphology, aggregation, surface charge, surface chemistry, and extract-derived surface-associated constituents. Nevertheless, the present findings do not establish a causal structure–activity relationship, composition-only causation, or synergistic interactions among the rare-earth constituents.

Demonstrating such relationships would require the corresponding single-rare-earth oxide controls, defined physical mixtures with matched elemental proportions, extract-only and appropriately washed-nanoparticle controls, direct nanoparticle–biomolecule interaction measurements, enzyme-kinetic studies, nanoparticle-interference controls, and relevant cellular and *in vivo* validation. The assay-specific IC_50_ estimates and applicable reference-to-multi-REO IC_50_ ratios are summarized in Table 5.

**Table 5:** Assay-specific IC_50_ estimates and reference-to-multi-REO IC_50_ ratios for multi-REO NPs, CeO_2_ NPs, and the corresponding reference compounds^a^

Assay endpoint	Multi-REO NPs IC_50_, µg mL^−1^ (95% CI)	CeO_2_ NPs IC_50_, µg mL^−1^ (95% CI)	Reference compound IC_50_, µg mL^−1^ (95% CI)	Endpoint interpretation	IC_50_ ratio (reference/multi-REO)
DPPH˙ scavenging	115.3 (98.4–135.3)	NR	71.4 (51.3–98.1), AA	50% response not reached	0.62
ABTS˙^+^ scavenging	88.2 (69.3–112.0)	NR	55.5 (34.6–85.4), AA	50% response not reached	0.63
NO˙ scavenging	186.1 (176.0–196.8)	NR	109.7 (92.8–129.9), AA	50% response not reached	0.59
HO˙ scavenging	NR	NR	143.1 (129.6–158.3), AA	Neither nanoparticle system reached 50% response	N.C.
H_2_O_2_ scavenging	173.0 (161.9–185.1)	NR	100.8 (83.0–122.3), AA	50% response not reached	0.58
α-Amylase inhibition	129.1 (111.9–149.1)	NR	79.57 (60.06–104.8), acarbose	50% response not reached	0.62
α-Glucosidase inhibition	NR	NR	129.0 (113.9–146.3), acarbose	Neither nanoparticle system reached 50% response	N.C.
BSA thermal-denaturation inhibition	159.6 (132.7–193.1)	NR	121.5 (94.3–157.1), diclofenac sodium	50% response not reached	0.76
AChE inhibition	N.D.	N.D.	109.4 (76.8–156.5), donepezil	No monotonic nanoparticle concentration–response	N.C.

a IC_50_ values are expressed in µg mL^−1^, with profile-likelihood 95% confidence intervals shown in parentheses. IC_50_ estimates and confidence intervals were reported only when the observed response reached or exceeded 50% within the tested concentration range of 50–200 µg mL^−1^ and the constrained concentration-response model yielded an interpretable estimate. NR indicates that 50% response was not reached within the tested range. NR does not represent an IC_50_ estimate of 200 µg mL^−1^ and does not establish that 50% response would necessarily occur above the tested range. N.D. indicates that an IC_50_ could not be reliably determined because the response lacked a meaningful monotonic concentration-dependent pattern or did not support an interpretable sigmoidal fit. The reference-to-multi-REO IC_50_ ratio was calculated by dividing the IC_50_ of the corresponding reference compound by the IC_50_ of multi-REO NPs, only when both estimates were interpretable and the multi-REO response reached or exceeded 50% within the tested range. Ratios below 1 indicate that multi-REO NPs required a higher concentration than the corresponding reference compound to achieve 50% response. These ratios are assay-specific comparative descriptors and should not be interpreted as therapeutic potency, statistical equivalence, or values directly comparable across assays with different reaction principles and reference compounds. For AChE, both nanoparticle systems produced low apparent inhibition values of approximately 19–22% without monotonic concentration-dependent responses; these percentages are not IC_50_ values. AA, ascorbic acid; AChE, acetylcholinesterase; BSA, bovine serum albumin; N.C., IC_50_ ratio not calculated; N.D., not determined; NR, 50% response not reached within the tested range.

### 3.11. Limitations and future perspectives

The present study provides a controlled comparison of CeO_2_-rich and multi-rare-earth-containing oxide-rich nanoparticles prepared using freshly and independently prepared aqueous extracts obtained from the same collected *Moringa oleifera* seed lot under standardized extraction and harmonized reaction and post-synthesis conditions. Nevertheless, several limitations should be considered when interpreting the compositional, physicochemical, colloidal, optical, and preliminary cell-free screening results.

First, the two nanoparticle systems were prepared from inherently different precursor chemistries: CeCl_3_·7H_2_O was used for the CeO_2_-rich system, whereas an LREO cake containing multiple rare-earth constituents was used for the multi-REO system. Although the extract batch and processing conditions were harmonized, the observed differences cannot be attributed exclusively to elemental composition. Precursor identity, rare-earth loading, accompanying ions or impurities, hydrolysis behavior, phase complexity, extract-derived surface-associated constituents, aggregation state, and specific surface area may also contribute. In addition, the test concentrations used in the cell-free assays were normalized by total nanoparticle mass rather than by particle number, specific surface area, total rare-earth content, or inorganic-core mass. Future studies should therefore determine product yield, rare-earth recovery, specific surface area, the surface-associated organic fraction, and bulk elemental composition and should consider dose normalization by total rare-earth content and/or accessible surface area.

Second, SEM-EDS provided semi-quantitative local elemental information and demonstrated the micrometre-scale regional co-occurrence of O, Ce, La, Nd, and Pr in the multi-REO sample, with Sm detected locally as a minor constituent in the elemental-mapping dataset. However, SEM–EDS does not establish bulk stoichiometry, atomic-scale elemental mixing, homogeneous incorporation of all detected elements within individual nanoparticles, or occupation of a common crystallographic lattice. The available XRD and SAED data likewise do not distinguish conclusively among a true mixed-oxide solid solution, a multiphase rare-earth oxide assemblage, physically associated rare-earth-containing domains, an oxide/oxyhydroxide-rich composite, or a poorly crystalline/amorphous rare-earth-containing precipitate. Accordingly, the product is described operationally as a Ce-rich, multi-rare-earth-containing oxide-rich nanoparticulate material rather than as a confirmed single-phase solid solution. Future studies should combine bulk elemental analysis of the final products by ICP-OES or ICP-MS with XPS, Raman spectroscopy, high-resolution STEM-EDS, and higher-resolution diffraction suitable for defensible full-pattern refinement.

Third, oxidation-state distributions and defect chemistry were not directly quantified. Interpretations involving Ce^3+^/Ce^4+^-related surface redox behavior, Pr oxidation states, oxygen vacancies, and defect-related surface sites therefore remain plausible, literature-supported hypotheses rather than experimentally established characteristics of the present materials. Oxidation-state- and defect-sensitive methods, including XPS, XANES, EPR, Raman spectroscopy, and/or EELS, would be required to clarify the oxidation states, local chemical environments, and defect characteristics and to distinguish their possible contributions to the optical and cell-free scavenging responses.

Fourth, DLS showed broad, polydisperse hydrodynamic distributions and aggregation in both nanoparticle suspensions. Zeta-potential measurements yielded −25.5 mV for CeO_2_ NPs and −5.09 mV for multi-REO NPs under the applied aqueous measurement conditions, indicating comparatively stronger electrostatic stabilization of the CeO_2_ suspension and weak electrostatic stabilization of the multi-REO suspension. However, zeta potential is medium-dependent and represents the electrokinetic potential at the slipping plane rather than the intrinsic charge of the solid surface. The present measurements do not establish long-term colloidal stability or predict behavior in physiological or serum-containing media. Future investigations should evaluate time-dependent aggregation, sedimentation, and charge evolution as functions of pH, ionic strength, particle concentration, temperature, and biomolecular composition using standardized dispersion and sonication procedures.

Fifth, the radical/reactive-species-scavenging, total antioxidant capacity, reducing power, enzyme-inhibition, and BSA thermal-denaturation assays were preliminary, assay-specific, cell-free screening models. Nanoparticle absorbance, light scattering, turbidity, sedimentation, reagent adsorption, and nonspecific nanoparticle–protein interactions may affect colorimetric endpoints, even when sample-specific and reagent blanks are applied. The observed responses should therefore not be interpreted as direct evidence of intracellular antioxidant effects, antidiabetic efficacy, anti-inflammatory efficacy, neuroprotection, or therapeutic activity. Future work should include expanded nanoparticle-interference controls, enzyme-kinetic analysis at multiple substrate concentrations, substrate-competition experiments, and direct measurements of nanoparticle–protein and nanoparticle–enzyme interactions.

Sixth, the aqueous *M. oleifera* seed extract was not evaluated as an extract-only control in the antioxidant assays. Consequently, the contribution of residual or surface-associated phytochemicals could not be quantitatively separated from that of the inorganic nanoparticle components. Targeted HPLC-DAD profiling identified and quantified selected phenolic and flavonoid constituents in the aqueous extract, and FTIR findings were consistent with extract-derived surface-associated functionalities; however, the identities and abundances of specific compounds bound to the final nanoparticle surfaces were not determined. Future studies should include extract-only, precursor-only, matrix-matched, and appropriately washed nanoparticle controls, together with direct characterization of surface-associated organic constituents.

Seventh, conventional molecular docking was not applied directly to the CeO_2_ and multi-REO nanoparticle systems because these materials are not discrete molecular ligands with uniquely defined structures. Their interactions with enzymes may depend on heterogeneous phase and surface composition, oxidation states, hydration, aggregation, surface charge, and the extract-derived interfacial layer. Docking studies of selected, structurally defined HPLC-detected constituents could generate hypotheses concerning possible ligand–enzyme interactions, but such calculations would describe the isolated organic constituents rather than the intact biogenic nanoparticles. Future computational work should therefore be combined with enzyme kinetics, direct binding analysis, extract-only controls, and appropriately characterized nanoparticle comparators.

Eighth, the cell-free assays included three independent experimental units per treatment. For each nanoparticle system, these experimental units consisted of three independently prepared synthesis batches, whereas the corresponding reference compounds were evaluated in three independent assay runs. Each independent synthesis batch or assay run was evaluated at 50, 100, and 200 µg mL^−1^, with experimental-unit identity maintained across the three concentrations. Where multiple measurements were obtained under a batch-concentration or run-concentration condition, these measurements were treated as technical replicates and averaged to obtain a single response value for the corresponding independent experimental unit. Technical replicates were not treated as independent observations and did not increase the inferential sample size. Treatment was analyzed as the between-unit factor, whereas concentration was analyzed as the within-unit repeated factor using two-way mixed-design repeated-measures ANOVA. Sphericity was not assumed, and the Geisser–Greenhouse correction was applied to the concentration and treatment × concentration effects. Nevertheless, the relatively small number of independent experimental units (*n* = 3 per treatment) limits the precision of variance estimates and the ability to assess the distributional and covariance assumptions underlying the statistical analysis.

Furthermore, only three concentrations were evaluated for concentration–response analysis. This limited concentration range restricts detailed characterization of the response curves and increases the dependence of the estimated IC_50_ values on the selected regression model and its constraints. The concentration–response data were fitted using a constrained sigmoidal model, with the lower and upper plateaus fixed at 0% and 100%, respectively, and the Hill slope fixed at 1. The reported IC_50_ values and their profile-likelihood 95% confidence intervals should therefore be regarded as model-dependent estimates and interpreted cautiously. IC_50_ estimates were reported only when the observed response reached or exceeded 50% response within the tested concentration range and the fitted model yielded an interpretable estimate. When 50% inhibition was not reached, the endpoint was designated as not reached within the tested range (NR), and no extrapolated numerical IC_50_ estimate or confidence interval was reported. When the response lacked a meaningful monotonic concentration-dependent pattern, the IC_50_ was designated as not determined (N.D.). Although independently prepared extract and nanoparticle batches were evaluated, all extracts were prepared from the same collected seed lot. Future studies should include seed lots obtained from different collection times, geographical sources, and storage periods to evaluate the reproducibility of the synthesis route across biological-source variability, evaluate additional concentrations—particularly around the expected IC_50_—and further characterize between-batch variability in composition, structure, surface properties, colloidal behavior, and cell-free screening responses.

Finally, the aqueous extract-mediated route uses water as the principal processing medium and comparatively mild reaction conditions of 60 °C for 2 h, which may be advantageous for future scale-up. However, scalability was not demonstrated in the present laboratory-scale study, and process yield, rare-earth recovery, mixing efficiency, energy consumption, waste generation, and techno-economic feasibility were not evaluated. Batch-to-batch variation in seed origin, maturity, storage, extraction yield, pH, total solids, protein content, and phytochemical composition may influence nanoparticle formation and surface functionalization. Variation in the LREO precursor composition may provide an additional source of variability. Future process-development studies should standardize the seed-to-water ratio, extraction temperature and duration, filtration, extract storage interval, pH, solid content, precursor composition, reagent-addition rate, mixing, purification, and drying. Independently prepared extract and nanoparticle batches should be compared with respect to product yield, rare-earth recovery, bulk composition, phase characteristics, particle-size distribution, surface chemistry, DLS, and zeta-potential measurements.

Overall, the present findings demonstrate clear differences between the two biogenic nanoparticle systems under the applied physicochemical and cell-free assay conditions, but they do not establish composition-only causation, true rare-earth synergy, biological safety, or therapeutic efficacy. Cellular uptake, protein-corona formation, cytotoxicity, hemocompatibility, oxidative-stress and inflammation-related cellular responses, glucose-metabolism models, metabolism, pharmacokinetics, biodistribution, and appropriately designed *in vivo* studies will be required before any biomedical or translational relevance can be established.

## 4. Conclusion

This study compared CeO_2_-rich nanoparticles and Ce-rich, multi-rare-earth-containing oxide-rich nanoparticles prepared in three independent synthesis batches using freshly and independently prepared aqueous extracts from the same collected *Moringa oleifera* seed lot under harmonized extraction, reaction, and post-synthesis conditions. The two nanoparticle systems exhibited distinct structural, morphological, optical, surface, and colloidal characteristics. XRD indicated limited long-range crystallographic ordering in the CeO_2_-rich material and more numerous, comparatively better-resolved diffraction features in the multi-REO material. TEM showed predominantly quasi-spherical CeO_2_ particles with projected dimensions of approximately 5–15 nm, whereas the multi-REO material contained more morphologically heterogeneous particles, including faceted, irregular, and short rod-like features with dimensions of approximately 20–60 nm. No Scherrer coherent-domain-size estimates were retained because the available diffraction features did not permit defensible peak-width determination.

SEM-EDS mapping demonstrated the micrometre-scale regional presence of Ce, La, Nd, and Pr in the multi-REO material, with Sm detected locally as a minor constituent in the elemental-mapping dataset. These findings support the operational description of the product as a Ce-rich, multi-rare-earth-containing oxide-rich nanoparticulate material but do not establish atomic-scale elemental mixing, homogeneous incorporation of all detected elements into a common crystallographic lattice, or single-phase solid-solution formation. FTIR findings were consistent with surface hydroxylation and extract-derived surface-associated functionalities, although the identities and abundances of individual surface-bound compounds were not determined directly. Both aqueous suspensions were polydisperse and exhibited aggregation. CeO_2_ NPs had a more negative zeta potential (−25.5 mV) than multi-REO NPs (−5.09 mV), indicating comparatively stronger electrostatic stabilization of the CeO_2_ suspension and weak electrostatic stabilization of the multi-REO suspension under the applied measurement conditions.

In the preliminary cell-free assays, multi-REO NPs generally produced greater apparent responses than CeO_2_ NPs in the radical- and reactive-species-scavenging assays, total antioxidant-capacity and reducing-power assays, α-amylase- and α-glucosidase-inhibition assays, and BSA thermal-denaturation assay. Multi-REO NPs exhibited estimated IC_50_ values of 115.3 µg mL^−1^ for DPPH˙ scavenging, 88.2 µg mL^−1^ for ABTS˙^+^ scavenging, 186.1 µg mL^−1^ for NO˙ scavenging, 173.0 µg mL^−1^ for H_2_O_2_ scavenging, 129.1 µg mL^−1^ for α-amylase inhibition, and 159.6 µg mL^−1^ for BSA thermal-denaturation inhibition. CeO_2_ NPs did not reach 50% response within the tested concentration range for these endpoints, and no extrapolated numerical IC_50_ estimates were retained. Neither nanoparticle system reached 50% response in the HO˙-scavenging or α-glucosidase-inhibition assays. In the AChE assay, both nanoparticle systems produced low apparent inhibition values of approximately 19–22% without monotonic concentration-dependent responses; consequently, their AChE IC_50_ values were not determined.

The observed differences may reflect the combined influences of precursor chemistry, rare-earth composition, phase complexity, particle morphology, aggregation, surface charge, surface chemistry, and extract-derived surface-associated constituents. However, the present findings do not establish composition-only causation, causal structure–activity relationships, or synergistic interactions among the rare-earth constituents. The data also do not establish specific oxygen-vacancy concentrations, Ce^3+^/Ce^4+^ distributions, or individual rare-earth oxidation states.

Because the biochemical assays were preliminary, assay-specific, and cell-free, the measured responses should not be interpreted as direct evidence of intracellular antioxidant effects, antidiabetic efficacy, anti-inflammatory efficacy, neuroprotection, biological safety, or therapeutic activity. Further studies should include a larger number of independently prepared extract and nanoparticle batches, bulk elemental analysis, higher-resolution phase- and oxidation-state-sensitive characterization, extract-only and appropriately washed-nanoparticle controls, expanded concentration ranges, nanoparticle-interference controls, enzyme-kinetic and direct nanoparticle–biomolecule interaction studies, and comprehensive cellular, biocompatibility, toxicity, and *in vivo* evaluations.

## Conflicts of interest

There are no conflicts to declare.

## Supplementary Material

NA-008-D6NA00394J-s001

## Data Availability

The data supporting the findings of this study are available within the article and its supplementary information (SI). Additional raw data, including numerical values used for figure generation and IC_50_ fitting, are available from the corresponding author upon reasonable request. Supplementary information: precursor-composition data, regional and mapping-derived SEM–EDS datasets, a descriptive comparison of the observed XRD features with reference positions, and HPLC-DAD chromatograms and integration reports for selected phenolic, phenolic-related, and flavonoid constituents. See DOI: https://doi.org/10.1039/d6na00394j.
