# Supplementary material for: Comparative evaluation of green-synthesized CeO2-rich and multi-rare-earth-containing oxide-rich nanoparticles prepared using Moringa oleifera seed extract: physicochemical characterization and preliminary cell-free screening
Source: Nanoscale Adv. 2026 Sep 25;8(21):6287–322. doi: 10.1039/d6na00394j (PMC13613366; doi:10.1039/d6na00394j)
Supplement: Supplementary file 1 [file NA-008-D6NA00394J-s001.pdf]

Supplementary Information (SI) for Nanoscale Advances.
This journal is © The Royal Society of Chemistry

Supplementary Information

Comparative Evaluation of Green-Synthesized CeO₂-Rich and Multi-Rare-Earth-Containing Oxide-Rich Nanoparticles Prepared Using *Moringa oleifera* Seed Extract: Physicochemical Characterization and Preliminary Cell-Free Screening

Nader Anis, Nada Emam, Mona M. Khalifa, and A. I. L. Abd El Fatah

Table S1. Reported elemental composition of the light rare-earth oxide cake (LREO cake) precursor.

Elements	Concentration (ppm)	Reported composition (%)
La (Lanthanum)	8,694	19.62
Ce (Cerium)	17,812	40.18
Pr (Praseodymium)	2,182	4.92
Nd (Neodymium)	12,222	27.56
**Total**	**40,910**	**92.28**

Note: The ppm values and reported composition percentages were reproduced from the available precursor-characterization dataset and follow the reporting basis used in that dataset. The listed constituents account for 92.28% of the reported composition; the remaining 7.72% was not assigned to individual constituents in the available precursor table. These values characterize the precursor and should not be interpreted as the bulk composition of the final multi-REO nanoparticle product.

Table S2. Regional semi-quantitative SEM–EDS spectrum-derived composition of the CeO_2-rich nanoparticulate material.

Element	Atomic %	Atomic % Error	Weight %	Weight % Error
O	67.0	0.6	21.5	0.2
P	0.8	0.1	0.5	0.1
S	4.6	0.2	3.0	0.1
Cl	1.1	0.1	0.8	0.1
Ce	26.4	0.1	74.3	0.3
**Total**	**100.0**	—	**100.1**	—

Note: Values represent a local, spectrum-derived SEM–EDS acquisition and should not be interpreted as the bulk stoichiometric composition of the sample. This regional dataset was obtained from an analyzed region and acquisition mode different from those used for the elemental-mapping dataset reported in Table S4 and Fig. 7. Instrument-reported error values represent analytical uncertainties rather than standard deviations from independently prepared nanoparticle batches. Totals may differ slightly from 100% because of rounding.

Table S3. Regional semi-quantitative SEM–EDS spectrum-derived composition of the multi-rare-earth-containing oxide-rich nanoparticulate material.

Element	Atomic %	Atomic % Error	Weight %	Weight % Error
O	72.9	0.8	26.9	0.3
P	1.8	0.1	1.3	0.1
Ca	4.4	0.1	4.1	0.1
La	4.8	0.1	15.4	0.4
Ce	10.2	0.1	32.8	0.3
Pr	2.2	0.1	7.2	0.3
Nd	3.7	0.1	12.4	0.3
**Total**	**100.0**	—	**100.1**	—

Note: Values represent a local, spectrum-derived SEM–EDS acquisition and should not be interpreted as the bulk stoichiometric composition of the sample. This regional dataset was obtained from an analyzed region and acquisition mode different from those used for the elemental-mapping dataset reported in Table S5 and Fig. 8. The instrument-reported error values represent analytical uncertainties rather than standard deviations from independently prepared nanoparticle batches. Totals may differ slightly from 100% because of rounding.

Table S4. Semi-quantitative SEM–EDS composition of the mapped CeO□-rich powder region (Site 90, Map 1).

Element	Atomic %	Atomic % Error	Weight %	Weight % Error
O	69.4	0.3	24.0	0.1
Si	0.3	0.0	0.2	0.0
P	1.8	0.0	1.2	0.0
S	5.0	0.0	3.5	0.0
Ca	0.0	---	0.0	---
La	0.2	0.0	0.6	0.0
Ce	23.1	0.0	69.9	0.1
Pr	0.2	0.0	0.6	0.0
Nd	0.0	---	0.0	---
Sm	0.0	---	0.0	---
**Total**	**100.0**	**—**	**100.0**	**—**

Note: Values represent the instrument-reported semi-quantitative SEM–EDS composition of the mapped CeO₂-rich powder region (Site 90, Map 1). The mapping acquisition was performed at 20 kV for 56 s using a map resolution of 768 × 512 pixels. Instrument-reported errors are analytical uncertainties and do not represent standard deviations from independent nanoparticle preparations. A dash (—) indicates that no error value was reported for an elemental contribution recorded as 0.0%. The dataset represents the investigated micrometre-scale region and should not be interpreted as bulk stoichiometric composition.

Table S5. Semi-quantitative SEM–EDS composition of the mapped multi-REO powder region (Site 87, Map 1).

Element	Atomic %	Atomic % Error	Weight %	Weight % Error
O	65.1	0.3	19.1	0.1
Si	0.4	0.1	0.2	0.0
P	2.5	0.1	1.4	0.1
S	0.2	0.1	0.1	0.0
Ca	1.7	0.1	1.2	0.1
La	6.9	0.1	17.7	0.1
Ce	14.2	0.2	36.6	0.4
Pr	2.0	0.0	5.2	0.1
Nd	6.0	0.2	15.9	0.6
Sm	0.9	0.1	2.5	0.2
**Total**	**99.9**	—	**99.9**	—

Note: Values represent the instrument-reported semi-quantitative SEM–EDS composition of the mapped multi-REO powder region (Site 87, Map 1). The mapping acquisition was performed at 20 kV for 76 s using a map resolution of 768 × 512 pixels. Instrument-reported errors are analytical uncertainties and do not represent standard deviations from independent nanoparticle preparations. The displayed atomic and weight percentages total 99.9% because of rounding. The dataset represents the investigated micrometre-scale region and does not establish bulk stoichiometry, nanoparticle-level co-localization, atomic-scale elemental mixing, homogeneous lattice incorporation, or single-phase solid-solution formation.

.

Table S6. Descriptive comparison of the observed XRD features with the nearest expected fluorite-CeO₂ reference positions.

Sample	Observed 2θ (°)	d-spacing (Å)	Nearest CeO_2 fluorite reference (hkl, 2θ °)	Δ 2θ (°)	Classification	Basis for classification
CeO_2 NPs	28.55	3.124	(111), 28.55	0.00	Tentative	Expected fluorite position; only a weak feature at/near background (~29.4 deg), not defensibly resolved.
CeO_2 NPs	n.r.	-	(200), 33.08	-	Unassigned	Expected fluorite position not resolved above local background.
CeO_2 NPs	47.47	1.914	(220), 47.49	0.02	Tentative	Very weak feature near expected position; near detection limit.
CeO_2 NPs	56.62	1.624	(311), 56.35	0.27	Tentative	Very weak feature near expected position; near detection limit.
CeO_2 NPs	36.27	2.475	-	-	Unassigned	No fluorite CeO_2 reference within 1.5 deg; not indexable from laboratory data.
CeO_2 NPs	8.50	10.394	-	-	Unassigned	Broad low-angle scattering (~7-12 deg); consistent with residual organic/amorphous contribution, not a CeO_2 reflection.

**Multi-REO NPs**	8.55	10.334	-	-	Unassigned	Low-angle feature not attributable uniquely to fluorite CeO₂; its phase origin could not be resolved from the available data.
**Multi-REO NPs**	13.27	6.667	-	-	Unassigned	Low-angle non-CeO₂ feature lacking a unique and defensible reference match.
**Multi-REO NPs**	18.39	4.821	-	-	Unassigned	Non-CeO2 reflection; not uniquely assignable from laboratory XRD.
**Multi-REO NPs**	26.05	3.418	-	-	Unassigned	Non-CeO2 reflection in the light-rare-earth oxide/oxycarbonate region; assignment not unique.
**Multi-REO NPs**	32.73	2.734	(200), 33.08	0.35	Overlapping	Near CeO2 (200) (dt∼0.35 deg) but offset; likely overlap of CeO2(200) with a non-CeO2 rare-earth phase.
**Multi-REO NPs**	44.13	2.051	-	-	Unassigned	No unique reference match within 1.5 deg.
**Multi-REO NPs**	48.19	1.887	(220), 47.49	0.70	Overlapping	Broad feature near CeO2 (220) (dt∼0.70 deg); overlapping/non-unique.
**Multi-REO NPs**	27.75	3.212	(1 1 1), 28.55	0.80	Unresolved/ unindexed	Weak feature offset from CeO2 (1 1 1); not defensibly resolved.

Note: The listed reference positions are provided for descriptive comparison only. Proximity to a reference position does not constitute definitive phase identification or Miller-index assignment. Weak, overlapping, broad, or non-unique features were retained as unresolved, unindexed, or unassigned. n.r., not resolved.

(A)

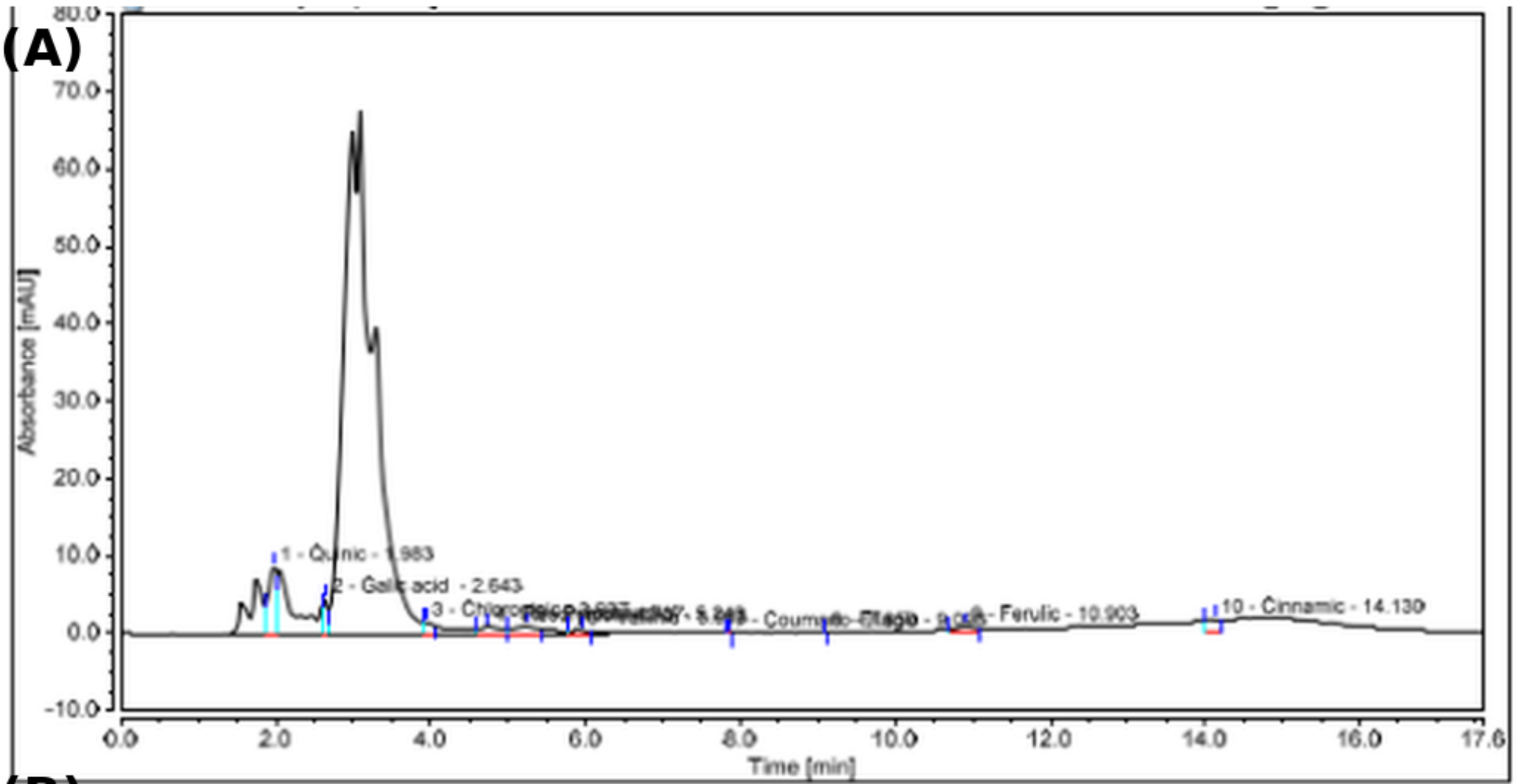

(B) Integration Results

No.	Peak Name	Retention Time min	Area mAU*min	Height mAU	Relative Area %	Relative Height %	Amount ppm
1	Quinic	1.983	1.155	8.714	35.51	42.78	12.33
2	Galic acid	2.643	0.293	4.641	9.03	22.78	2.29
3	Chloroginic	3.937	0.233	1.593	7.17	7.82	3.56
4	Resorcinol	4.737	0.334	0.988	10.26	4.85	6.66
5	Pyrocatechol	5.243	0.386	1.032	11.87	5.07	20.57
6	Vailinic	5.943	0.158	0.560	4.87	2.75	0.44
7	Coumaric	7.850	0.003	0.080	0.11	0.39	0.01
8	Ellagic	9.093	0.004	0.104	0.12	0.51	0.01
9	Ferulic	10.903	0.267	0.844	8.22	4.14	1.10
10	Cinnamic	14.130	0.418	1.815	12.85	8.91	1.49
**Total:**			**3.251**	**20.371**	**100.00**	**100.00**	

Fig. S1. HPLC-DAD profiling of phenolic and related compounds in the aqueous *Moringa oleifera* seed extract. **(A)** Representative chromatogram recorded at 254 nm, showing peaks assigned by comparison of retention times and UV absorption characteristics with authentic standards. **(B)** Corresponding chromatographic integration results, including retention time, peak area, peak height, relative area, relative height, and instrument-reported amount. Peak assignments: 1, quinic acid; 2, gallic acid; 3, chlorogenic acid; 4, resorcinol; 5, pyrocatechol; 6, vanillic acid; 7, coumaric acid; 8, ellagic acid; 9, ferulic acid; and 10, cinnamic acid. The "Amount (ppm)" values shown in

the instrument-generated integration report refer to the injected analytical solution. The corresponding values reported in Tables 2 and 3 of the main manuscript were converted to $\mu g\ g^{-1}$ of the analyzed sample using the applicable sample-preparation and concentration factors.

(A)

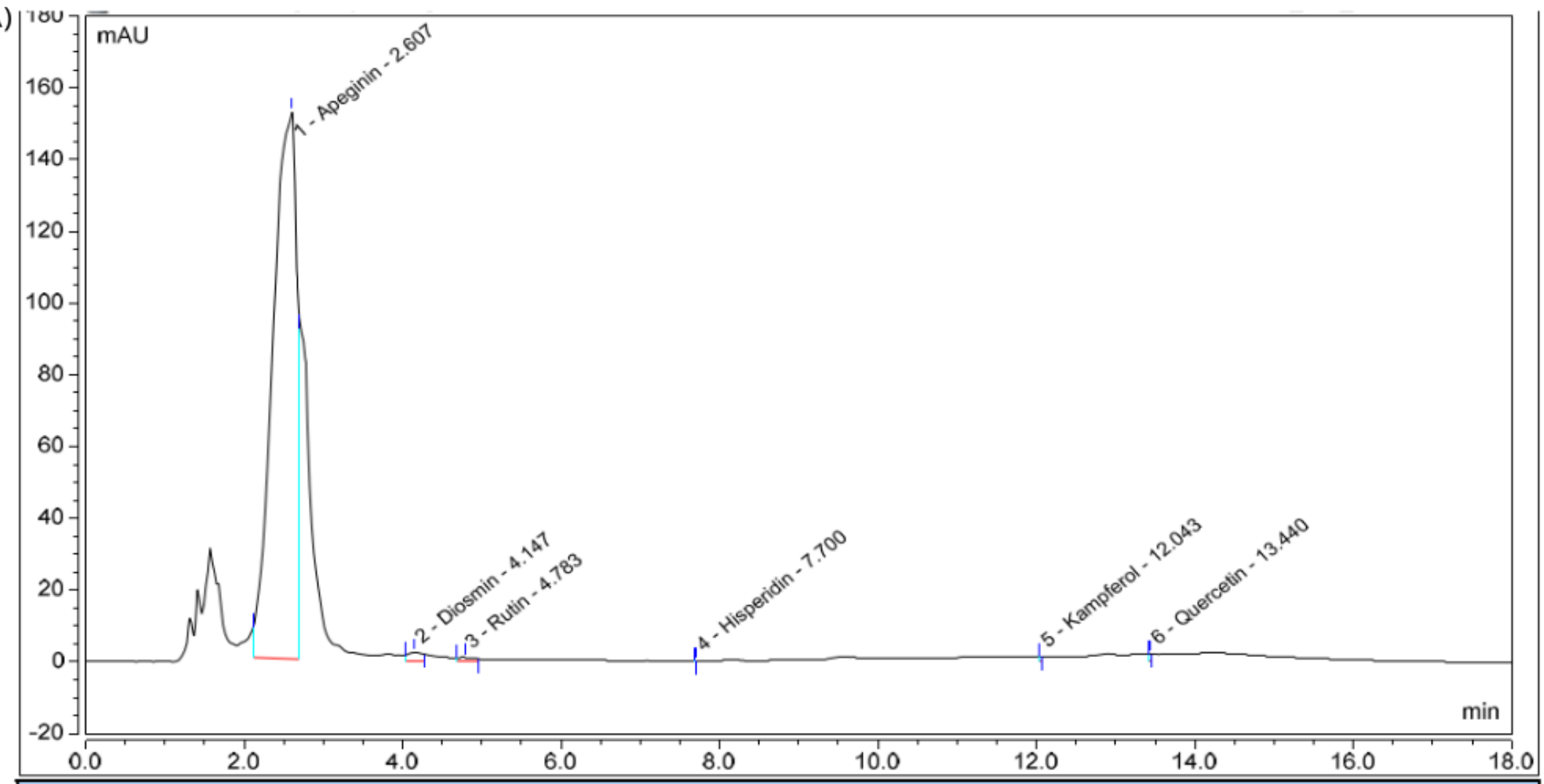

(B)

Integration Results

No.	Peak Name	Retention Time min	Area mAU*min	Height mAU	Relative Area %	Relative Height %	Amount ppm
1	Apeginin	2.607	51.689	152.401	98.11	95.40	1152.4420
2	Diosmin	4.147	0.562	2.518	1.07	1.58	0.0275
3	Rutin	4.783	0.288	1.099	0.55	0.69	0.2065
4	Hisperidin	7.700	0.007	0.266	0.01	0.17	0.0041
5	Kampferol	12.043	0.050	1.380	0.10	0.86	0.0043
6	Quercetin	13.440	0.090	2.078	0.17	1.30	0.1117
**Total:**			**52.687**	**159.742**	**100.00**	**100.00**	

Fig. S2. HPLC-DAD profiling of flavonoids in the aqueous *Moringa oleifera* seed extract. **(A)** Representative chromatogram recorded at 254 nm, showing peaks assigned by comparison of retention times and UV absorption spectra with authentic standards. **(B)** Corresponding chromatographic integration results, including retention time, peak area, peak height, relative peak area, relative peak height, and instrument-reported amount. Peak assignments: 1, apigenin (2.607 min); 2, diosmin (4.147 min); 3, rutin (4.783 min); 4, hesperidin (7.700 min); 5, kaempferol (12.043 min); and 6, quercetin (13.440 min). Apigenin was the predominant integrated flavonoid peak, accounting for 98.11% of the total integrated peak area. Identifications were based on the available authentic standards and should be interpreted as targeted assignments rather than comprehensive

characterization of the extract metabolome. Because the assignment of the dominant peak was not confirmed by standard addition, peak-purity analysis, or mass spectrometry, its identity and calculated concentration should be interpreted cautiously. The "Amount (ppm)" values shown in the instrument-generated integration report refer to the injected analytical solution. The corresponding values reported in Table 3 of the main manuscript were converted to $\mu g\ g^{-1}$ of the analyzed sample using the applicable sample-preparation and concentration factors.
